# A computational method to predict genetically encoded rare amino acids in proteins

**DOI:** 10.1186/gb-2005-6-9-r79

**Published:** 2005-08-31

**Authors:** Barnali N Chaudhuri, Todd O Yeates

**Affiliations:** 1 UCLA-DOE Institute for Genomics and Proteomics and Department of Chemistry and Biochemistry, University of California, Los Angeles, USA

## Abstract

In several natural settings, the standard genetic code is expanded to incorporate two additional amino acids with distinct functionality, selenocysteine and pyrrolysine. These rare amino acids can be overlooked inadvertently, however, as they arise by recoding at certain stop codons. We report a method for such recoding prediction from genomic data, using read-through similarity evaluation. A survey across a set of microbial genomes identifies almost all the known cases as well as a number of novel candidate proteins.

## Abstract 2

A new method for predicting recoding by rare amino acids such as selenocysteine and pyrrolysine was used to survey a set of microbial genomes.

## Background

Codon redefinitions that expand upon the standard genetic code beyond the 20 canonical amino acids are reported in all three domains of life [1,2]. Two known genetically encoded rare amino acids (RAAs) are selenocysteine and pyrrolysine, the proposed 21^st ^and the 22^nd ^amino acids, respectively [3-7]. Selenocysteine, a selenium-analog of cysteine, is a potent nucleophile [5] and has been reported in organisms as diverse as *Escherichia coli *and human beings [4,5]. Selenium plays a dual role in nature as an essential micronutrient in human health, and as an environmental hazard to humans, livestock and wildlife [8] when it is present in high amounts. Thus, selenium is a target for both molecular biology and bioremediation research [8,9]. The distribution of selenium in the form of selenocysteine residues [5,10] in specific proteins is not completely understood. Pyrrolysine is a recently discovered amino acid in the methanogenic archaeon *Methanosarcina barkeri*, where it supposedly plays a critical role in methyltransferase chemistry as an electrophile [6,7]. Traditional genomic sequence analyses tend to overlook these RAAs, leading to mis-annotation in the sequence databases. Systematic bioinformatic investigations of the genomic data offer the possibility of understanding which organisms utilize RAAs, and which proteins in particular incorporate them into their structures.

**Figure 1:**
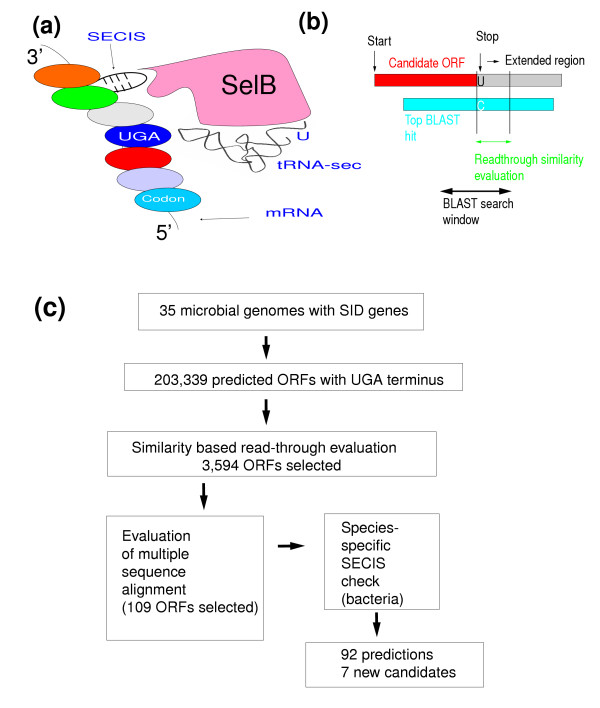
Schematic representation of the selenocysteine insertion machinery and the selenoprotein detection scheme. **(a) **A cartoon diagram of selenocysteine incorporation during protein translation inside the cell. The selenocysteine-specific elongation factor (SelB; pink) is shown interacting with the selenocysteine insertion sequence (SECIS) hairpin element in the mRNA and tRNA-sec (SelC). The anticodon of SelC tRNA interacts with and recognizes the 'UGA' codon. The ribosome and other components of the translational machinery are omitted for clarity. **(b) **Schematic representation of the 'read-through similarity analysis' approach. The top BLAST hit is shown in blue. The window lengths used for the BLAST search and read-through similarity evaluation are marked in the drawing. **(c) **A flow chart describing how the different components of the predictive scheme are combined for selenoprotein prediction. ORF, open reading frame.

Predicting which natural proteins contain the RAA selenocysteine or pyrrolysine on the basis of genomic sequence data is a difficult problem [2]. The difficulty arises from the distinction that, unlike other amino acids, RAAs are not coded for by dedicated codons. Instead, they are incorporated in special circumstances by the UGA (opal; selenocysteine) and the UAG (amber; pyrrolysine) codons [3-7], which are ordinarily interpreted as stop signals to terminate translation (Figure 1a). From a genomics point of view, the problem is how to discriminate between all the true stop signals in genomic sequence data, and those cases that signal for incorporation of a RAA. At the mRNA level, one feature referred to as the selenocysteine insertion sequence (SECIS) hairpin motif is understood to signal for selenocysteine insertion. The situation is greatly complicated, however, by the divergence of the signal between different proteins and between different organisms with respect to the sequence and position of the signaling element, situated in either the 3' or 5' untranslated region of a recoded open reading frame (ORF; archaea/eukaryotes) or downstream of the recoded UGA (bacteria). Much less is understood about the newly discovered pyrrolysine incorporation machinery. The presence of a PYLIS (SECIS-equivalent) *cis*-acting element [2], and competition between translational termination and read-through, have been anticipated [11].

A number of earlier studies by Gladyshev and coworkers [12-16] have addressed the problem of predicting selenoproteomes, producing sets of selenoproteins encoded in various genomes. Systematic selenocysteine predictions in prokaryotes have been based on two criteria: alignment of the 'UGA' codon in the mRNA sequence with cysteine in homologous proteins in a pair-wise sequence alignment (henceforth, the cysteine alignment criterion), and the detection of a consensus SECIS signal in the nucleotide sequences (henceforth, the SECIS criterion). Both methods performed very well with near-zero false negatives [13,16]. Nevertheless, certain aspects of these approaches make them less suitable for generalized applications. For example, they cannot be applied to selenoproteins that fail to fit the cysteine alignment criterion (those selenoproteins that do not have a homolog in the database with a cysteine residue taking the place of the selenocysteines). The SECIS criterion also presents some limitations. High numbers of false positives arise with the genome-wide prediction of short, local RNA folding motifs, such as the SECIS element [17]. The observation that different organisms have divergent signals for selenocysteine insertion complicates the problem further [13,16]. Other models that do not rely on the identification of specific recoding signals, such as evaluation of the coding potential of the nucleotide sequence beyond the UGA termini, have been developed for eukaryotes [14]. To overcome the various difficulties associated with the detection of rare selenoproteins from genomic data, a combination of strategies is shown to be advantageous [2,14]. A database homology search using the entire lengths of candidate genes with an in-frame UAG codon has been employed recently for analyzing the nature of pyrrolysine decoding in methanogens [11].

Here we expand upon ideas developed by Gladyshev and colleagues [12-16], and introduce a new, multi-component scheme for microbial selenocysteine and pyrrolysine prediction. Several criteria are combined in series, including a new predictive element, 'read-through similarity analysis' (RSA; Figure 1b). The RSA criterion is applied in the early stage of the procedure to evaluate the read-through potential of an ORF based on an analysis of sequence similarity involving the hypothetical amino acid sequence translated beyond the candidate stop codon. This scheme is model-free, in the sense that it does not rely on any special RNA context, read-through mechanism, or incorporation of any particular amino acid residue at the recoding site. Following the RSA analysis, subsequent criteria (for example, cysteine alignment and SECIS) can be enforced, or overridden in special cases where the other criteria provide compelling evidence for a bona-fide read-through situation. Success of this predictive approach is not, therefore, strictly contingent on the presence of a protein homolog containing a cysteine substitution in the database or on a canonical SECIS motif in the case of selenoproteins. In addition to almost all of the known cases of UGA-encoded selenocysteines (Table 1), the present method successfully identifies several proteins with UAG-encoded pyrrolysine (Table 2), including novel candidates, as well as instances of genome-wide redefinition of UGA as a particular amino acid, such as tryptophan in *Mycoplasma *spp. The generality and wide applicability of the present approach makes it well suited to the critical problem of analyzing the rapidly growing number of new genomes.

## Results and discussion

### The selenoprotein prediction scheme

Our selenoproteome prediction scheme was developed based on the expectation that a putative selenoprotein will satisfy the following, specific conditions. It should show: a significant 'read-through similarity' (see below); an alignment of the selenocysteine residue with semi-invariant cysteine residue(s) in a set of aligned homologs; and a hairpin motif (putative SECIS) near the candidate ORF, which is consistent with the hairpin motifs near the other selenoproteins found in the same organism. The components of the predictive approach are combined as shown in Figure 1c. The RSA method incorporates an analysis of the protein sequences following the presumptive stop codons in a genome (Figure 1b). Due to the recoding of UGA as a selenocysteine, the sequence following the UGA codon would be translated as the carboxy-terminal part of an extended protein. This makes it possible to identify candidate selenoproteins in situations where the putative protein sequence immediately following a UGA codon is statistically similar to the aligned region of another homologous protein in a protein sequence database. The statistical detection of sequence homology in relatively short regions following the presumptive stop codon is achieved using a modified interpretation of standard dynamic alignment methods [18,19] (see Materials and methods section).

A search for selenoproteins was restricted to those organisms that contain at least one of the genes that are required for synthesizing selenoproteins [3,4]. A set of 35 microbial genomes that have one or more of the three essential components of the selenocysteine insertion device (SID; SelA, the seryl tRNA selenium transferase; SelB, the elongation factor; and SelC, the sec-tRNA gene) were used (see Additional data file 1 for a list). The labile selenium donor selenophosphate synthetase (SelD) was not included as part of the SID because it can be a selenoprotein itself.

The RSA method was applied to all the predicted theoretical ORFs (length ≥ 90 residues) that contain an in-frame UGA stop codon. Out of a total 203,339 ORFs analyzed, 3,594 satisfied the test for likely similarity in the read-through region. These were subjected to further analysis.

Multiple sequence alignments (MSAs) were used as a subsequent step in analyzing the candidate selenoproteins, following the cysteine alignment criterion [13]. Cysteine residues often play special functional roles in proteins, such as in nucleophilic attack, or in metal coordination. A selenocysteine residue can substitute for a cysteine residue in these functional roles [10]. Functionally important residues usually form the most conserved features in a MSA. Therefore, we expect selenocysteine to align with conserved or semi-conserved residues (cysteines and selenocysteines) in homologous proteins. The MSA analysis step detected 109 candidate ORFs for further scrutiny.

As a final test, candidate selenoprotein genes were subjected to SECIS-element detection. Unlike archaea or eukaryotes, bacterial SECIS sequences are less conserved, thus complicating a search for a canonical SECIS profile [13], although a consensus bacterial SECIS model has been recently reported [16]. We used a fast, heuristic-based search [20] for a short hairpin motif common to a set of short, un-aligned mRNA segments downstream of the 'UGA' codon of the candidate selenoprotein ORFs in each bacterial organism (see Materials and methods section). The underlying assumption is that the SECIS elements in all the candidate mRNA strings within a given organism will have somewhat conserved primary (sequence) and secondary (base-paired) structures, so they can be recognized by the SID machinery in that organism. Thus, non-SECIS sequences should be distinguishable from well-aligned SECIS elements within an organism. This step was very useful in rejecting false positives when two or more *bona fide *selenoproteins were detected in an organism. In archaeal microbes, SECIS motif detection was not performed by the above method, as the SECISearch [12,13] program described earlier was sufficient.

### The predicted selenoproteins

**Figure 2:**
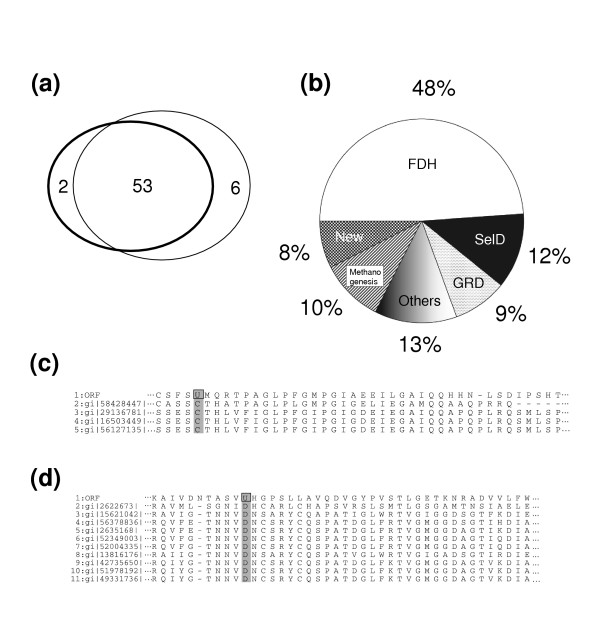
An overview of the predicted selenoproteome. **(a) **A Venn diagram representation of the overlap between the known selenoproteins in the RECODE database (bold line) and the results of our prediction method (plain line) over the same set of organisms as included in RECODE. **(b) **A pie chart illustrating the types of selenoproteins in our predicted dataset. The dataset was divided into the following groups: formate dehydrogenase (FDH) family enzymes; archaeal methanogenesis selenoproteins (excluding the FDH family); selenophosphate synthetase (SelD); other known selenoproteins (for example, thioredoxin, hesB); glycine reductase genes (GRD); and new candidate selenoproteins. **(c) **A section of the multiple sequence alignments (MSA) of the newly predicted candidate selenoprotein from *P. profundum *with its four homologs found in our database. Note the alignment of putative selenocysteine (U denotes selenocysteine) with cysteine residues in the MSA. **(d) **The MSA of a selenoprotein formylmethanofuran dehydrogenase from *M. maripaludis *in which the recoded selenocysteine aligns with a set of conserved aspartate residues rather than the cysteine residues. The MSA illustrations were prepared using ALSCRIPT [39].

The multi-step selenoprotein prediction scheme was highly successful in detecting a large number of known selenoproteins in a range of organisms (Table 1; Figure 2a). A comparison of the number of selenoproteins detected by our method versus the existing selenoprotein entries in the database of recoded proteins for those organisms (RECODE [21]) is shown in Figure 2a. About 96% (estimated sensitivity) of the RECODE entries (53 out of 55) were successfully predicted. Approximately 90% (estimated specificity) of the selenoproteins predicted here belong to previously known families. Amongst the proteins identified, it was noteworthy that a remarkably high number (approximately 48%) of selenoproteins fall within the formate dehydrogenase (FDH) protein family (Figure 2b). FDH is a member of the molybdopterin-dependant FDH/DMSO reductase superfamily of homologous enzymes in the SCOP classification [22]. Several ORFs showed the presence of -CxxC- or -CxxCxxC- motifs typical of a special subset of redox proteins in which one of the cysteines is replaced with a selenocysteine. Consistent with earlier reports [13,23], a set of selenoproteins was identified in a group of methanogenic archaea (Table 1), including *Methanococcus jannaschii*, *Methanopyrus kandleri *and *Methanococcus maripaludis*. Apart from an almost complete coverage of all the known selenoproteins, our method identifies seven additional likely selenoproteins (Table 1) for further experimental validation.

Although our method was highly successful in detecting almost all of the selenoproteins in the known database, it could not detect two known selenoproteins. The first one was a *SelD *gene in *Campylobacter jejuni *that could not be identified due to a sequence error in the genomic data [16]. The second one was the radical S-adenosylmethionine (SAM) domain protein in *Geobacter sulfurreducens*. Here, the selenocysteine residue is situated too close to the carboxyl terminus, thus causing a very low RSA Z-value (1.8). This is a true false negative and illustrates a shortcoming of relying on read-through similarity.

**Figure 3:**
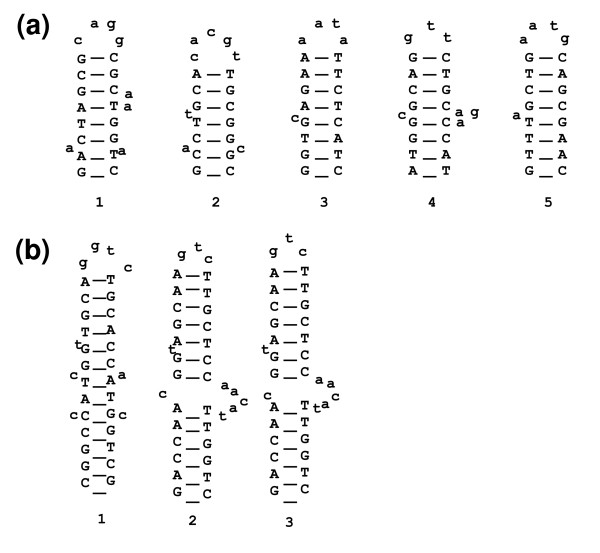
Representatives of the putative selenocysteine insertion sequence (SECIS) hairpin elements in various genomes as identified by the present study. **(a) **The SECIS elements from the genes coding for the following proteins from *P. profundum*: 1, glycine reductase GrdA; 2, glycine reductase GrdB2; 3, glycine reductase GrdA; 4, selenophosphate synthetase (SelD); 5, a hypothetical protein. **(b) **The SECIS elements from the genes coding for the following proteins from *E. coli*: 1, formate dehydrogenase; 2, formate dehydrogenase-N; 3, formate dehydrogenase-O.

One advantage of the generalized RSA approach over the existing SECIS search-based methods is its ability to detect selenoproteins with non-standard SECIS motifs. This requires overlooking the SECIS criterion, which is made possible in the present approach by the power and selectivity of the other two criteria (RSA and cysteine alignment). We were able to detect all four known selenoproteins in the piezophile *Photobacterium profundum *[24], two of which could not be detected by the SECIS criterion [16] due to the presence of a divergent SECIS element. In addition, a fifth candidate selenoprotein is identified here (Figure 2c), which had a divergent SECIS element and whose predicted selenocysteine residues line up with cysteine in all four homologous proteins identified. Putative SECIS motifs for these four selenoproteins and the additional candidate in *P. profundum *are presented in Figure 3a.

A second advantage of the RSA-based approach is the potential ability to detect selenoproteins that are not represented in the database by a homologous protein with a cysteine in the position corresponding to the presumptive stop codon. A close look at the multiple sequence alignments of certain selenoprotein homologs in the Conserved Domain database [25] indicated that nucleophilic serine, aspartate and glutamate residues sometimes replace the catalytic cysteine functionality. Unlike the previously described cysteine alignment criterion [13], the RSA-based approach does not analyze cysteine/selenocysteine alignment in an early stage. The presence of these conserved, non-cysteine residues aligned with putative selenocysteine can, therefore, be analyzed while inspecting the MSA, followed by an analysis of the SECIS feature. The protein formylmethanofuran dehydrogenase in *M. maripaludis *provides an example of a verified selenoprotein that is detected by our method without invoking the cysteine/selenocysteine alignment criterion (Figure 2d). The subject selenocysteine aligns with a set of aspartate residues in the MSA. However, glycine reductase A (GrdA), a selenoprotein whose homologs do not have cysteine in place of selenocysteine [13], could not be identified using our method on a test run. This failure resulted from a crucial lack of significant read-through similarity between GrdA and the other proteins homologous to GrdA.

A small number of ORFs (see Additional data file 2) were found with the translated UGA codon (U) aligned with strictly invariant nucleophilic residues (aspartate, glutamate or serine) in the MSA. None of these ORFS belong to previously known selenoprotein families or had a convincing SECIS motif adjacent to the UGA codon. Because of the lack of any additional evidence, it is not possible to further separate the true read-through events from the false-positives that might arise from statistical uncertainty or sequencing error. Nevertheless, some of these ORFs could be genuine read-through cases.

### Putative pyrrolysine recoding in archaea

**Figure 4:**
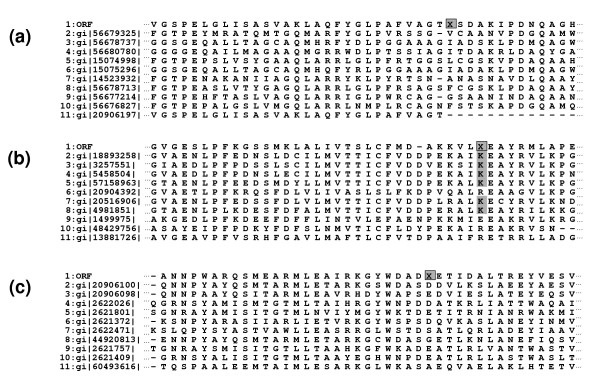
Sections of the multiple sequence alignments of the putative pyrrolysine-containing proteins. **(a) **A protein known to use UAG read-through, methylamine methyltransferase from *M. acetivorans*. **(b) **A putative methyltransferase from *M. burtonii*. **(c) **A predicted read-through ORF homologous to a cobalamin biosynthesis protein CobN (gi|20906100|gb|AAM31298.1|, Methanosarcina mazei Goe1) from *M. acetivorans*. Note the alignment of presumed pyrrolysine residues (denoted as X) with various amino acids.

The RSA method was also used to search for proteins potentially containing the pyrrolysine residue, the so-called 22^nd ^amino acid (Table 2). The pyrrolysine amino acid residue was recently discovered to be encoded by the UAG (amber) codon in the monomethylamine methyltransferase enzyme in *Methanosarcina barkeri*, where it serves as an electrophile to methylate the cobalt-corrinoid cofactor [6,7,26]. First, a search for homologs of the *PylS *gene (which codes for the pyrrolysine-specific aminoacyl tRNA synthetase [6,7]) in the available genomic data identified several methanogenic archaea as organisms likely to encode pyrrolysine containing proteins. These organisms include: *Methanosarcina barkeri fusaro, Methanosarcina acetivorans, Methanosarcina mazei *and *Methanococcoides burtonii*. Putative pyrrolysine-containing methylamine methyltransferses from methanogenesis pathways have been reported in this same set of organisms [11,26]. Within these four organisms, a total of 34 ORFs containing putative pyrrolysine residues were found to exhibit significant read-through similarity to homologous methyltransferases (Table 2). Out of 2,086 and 3,611 theoretical ORFs (see Materials and methods section) analyzed in the complete genomes of *M. mazei *and *M. acetivorans*, 87 and 97, respectively, showed significant read-through similarity. We have listed all those ORFs with an in-frame UAG codon that exhibit high RSA similarity, as well as well-aligned MSA for *M. acetivorans *and *M. mazei *(Additional data file 2). Apart from previously described transposases [11], the list contains several other candidate proteins, including a novel homolog of the cobalamin biosynthesis protein CobN (Figure 4c).

### Overall distribution of the recoded proteins

A marked tendency was noted for the selenoproteins to occur in certain pathways and functional categories (Figure 2b). The majority of detected selenoproteins in bacteria were FDHs that convert formate to carbon dioxide in anaerobic environments [27]. Other known selenoproteins include SelD, GrdA and GrdB (from the anaerobic glycine reduction pathway), HesB (associated with the nitrogen fixation genes), and several oxidoreductases (for example, thioredoxin and peroxiredoxin). In archaea, selenocysteine usage appears to be confined to a small group of enzymes in the anaerobic methanogenesis pathway [23] (such as FDH and formylmethanofuran dehydrogenase from the FDH family, and heterodisulfide reductase) that have conceivably co-evolved under similar evolutionary constraints in a number of methanogens. Pyrrolysine-encoding is found in methyltransferases [26] from a pathway that converts methylamines to methane in *Methanosarcina *sp. and in the Antarctic archaeon *M. burtonii*. A high incidence of unusual stop codon reassignments, both selenocysteines and pyrrolysines, in methanogenesis enzymes in ancient archaea is intriguing.

### Relative merit of the RSA-based approach

The selenoprotein identification scheme presented herein (an 'RSA-first, SECIS-later' approach) differs from the previously reported methods in several ways. Earlier studies (based on the SECIS search approach) provided an estimated rate of false SECIS hits to be 3 to 15 per 10 Mb [12,17] in eukaryotes, greatly surpassing the number of true selenoproteins. An improved result has been obtained by using a statistical profile computed from a training dataset of aligned known SECIS elements in metazoa [17]. A recent bacterial SECIS-search method analyzed 48,472 SECIS hits in a set of 29 organisms (representing 1.5% of all the UGA codons analyzed), out of which 28,974 (approximately 60%) were selected for further analysis of protein sequence conservation in the UGA flanking regions [16]. Still, difficulties remain for approaches that rely on detecting small RNA signal sequences as an early step in analysis, especially in situations such as new genomes, where the nature of the signal may not be understood in advance. Examining presumptive protein sequences as a prior step mitigates these difficulties. In the present study, although RSA was applied to fairly small segments of the protein sequences following the UGA codon, it was quite efficient in identifying candidates representing read-through events (Figure 1c). This ability of RSA to limit the predicted set to a relatively small, manageable number of likely candidates (3,594 out of 203,339, approximately 1.7%) facilitated further detailed calculations in genome-wide analyses. Of the small set of 109 ORFs selected by the subsequent MSA analysis, 92 (approximately 84%) were selected afterward as putative selenoproteins. Thus, an analysis of protein sequences is able to filter out most of the false-positives, without using any mRNA context information. Our combined 'RSA-first, SECIS-later' method is, therefore, applicable to cases (for example, *P. profundum*) where a divergent signal makes a SECIS-based search unsuitable [16]. In the present approach, it becomes possible to scrutinize putative non-canonical SECIS signals. In addition, our method provides a useful way to search for selenoproteins lacking homologs containing corresponding cysteine residues [13] (Figure 2d).

The RSA approach was likewise successful in predicting putative pyrrolysine-proteins in archaea. Out of the 9,515 theoretical ORFs analyzed for putative pyrrolysine residues in four methanogens, 321 ORFs (3.4%) displayed significant read-through similarity. Unlike the case for selenoproteins, a reliable benchmarking of pyrrolysine-protein predictions against a known dataset was not possible. The predicted result encompasses the previously reported methylamine methyltransferases [26], however, and includes a number of likely candidates for further experiments. Intriguingly, the putative pyrrolysine residues do not align so exclusively with a particular, conserved amino acid in homologous proteins (Figure 4a-c) [11]. The RSA method appears, therefore, to be generally useful as an initial predictor for pyrrolysine proteins. In addition, the RSA approach offers wider utility for identifying cases of genome-wide stop codon redefinition (for example, in *Mycoplasma *spp.; see Materials and methods section) or special instances of stop codon read-through (for example, UAG read-through in a pilus biosynthesis gene in *E. coli *[28] (data not shown)).

## Conclusion

To summarize, we have developed a novel computational scheme for predicting selenocysteine and pyrrolysine residues in proteins and have applied the method to microbes with complete genomes. In addition to confirming well-known examples, our method predicts new prospective candidates for further experimental validation. A worldwide web site has been developed for the interested user community [29]. The method should be a useful tool for predicting rare amino acids, as well as other read-through events, and for correcting gene annotations in the growing genomic databases.

## Materials and methods

All the complete genomes were obtained from the National Center for Biotechnology Information (NCBI) [30]. Unfinished *M. barkeri *and *M. burtonii *genomes were obtained from The Institute for Genomic Research [31]. A list of accession numbers is provided in the Additional data files. A Perl script was written to perform all the computations (available upon request). All computations were performed in a local cluster of Linux computers. tRNA genes were computationally identified using the tRNASCAN-SE program [32]. Genes encoding SelA and SelB were detected directly from annotated genomes from NCBI.

All theoretical ORFs (≥ 90 residues) that begin with a start codon (ATG, TTG or GTG) and end with a stop codon (TAA, TAG or TGA) and contain one in-frame TGA (for selenocysteine) or TAG (for pyrrolysine) codon were extracted from the genomic data for analysis. In order to detect two short SelW proteins in *G. sulfurreducens *and *C. jejuni*, a reduced (80 residue) length constraint was used.

### Read-through similarity analysis (RSA)

For each of the predicted ORFs, the BLAST program [33,34] was used to search for homologous proteins in a customized sequence database. The BLAST search space was restricted to a window of a maximum 100 residue length, pivoting at the stop codon. The BLOSUM62 matrix was used throughout and the selenocysteine residue was treated as 'any amino acid' (X). The BLAST database contained a maximum of 650,870 protein sequences from all the annotated complete microbial genomes from NCBI (dated 4 December 2005). A self-excluding BLAST database was used for the homology search in each organism. Top hits (E-value ≤ 10^-1^) that encompassed either side of the stop codon were identified. For each of the selected, truncated ORF sequences ({x_1_, x_2_,..., x_i_,..., x_u_,..., x_n_} where n = min{u + 60, u + t}; u = position of the stop codon; t = position of the subsequent stop codon) and the corresponding top hit sequence from the BLAST search ({y_1_, y_2_..., y_j_,..., y_m_}), a (n + 1) by (m + 1) dynamic alignment matrix was calculated with an affine gap penalty function [18,19]. N-terminal overhangs for both the sequences were not penalized; the 0^th ^row and the 0^th ^column were initialized with zero values.

For each cell (i,j) in the matrix:

   a(i,j) = s(i,j) + max { a(I - 1,j - 1),

         b(i-1,j-1),

         c(i-1,j-1) }

   b(i,j) = max { -(h + g) + a(i,j - 1),

            -g + b(i,j - 1),

      -(h + g) + c(i,j - 1) }

   c(i,j) = max { -(h + g) + a(I - 1,j),

      -(h + g) + b(I - 1,j),

            -g + c(I - 1,j) }

   score (i,j) = max {a(i,j), b(i,j), c(i,j)}; s(i,j) → BLOSUM62 matrix; h = 12, g = 2

   Best_score_ORF _= max{score(n,j), j = 1,..,m}     (1)

Because we were exclusively interested in the significance of the alignment at the carboxy-terminal extension region beyond the stop position, the highest score from the n^th ^column (that is the alignment of the terminal residue x_n _of the truncated ORF with the {y_1_,..., y_m_} residues) was taken as the maximal score (Best_score_ORF_) instead of the usual Smith-Waterman score. A Z-value was computed by shuffling the terminal extension region 100 times, re-computing the scores in the terminal block of the matrix ({x_stop_,..,x_n_} and {y_1_,..,y_m_}) and averaging the maximal score (<Best_score_rand_>). A test calculation with 10,000 times shuffling for one genome produced similar results. The values from randomized sequences were used to calculate a Z-value:

Z_ORF _= (Best_score_ORF _- <Best_score_rand_>)/standard_deviation     (2)

A generally weak dependence on length and amino acid composition makes the Z-values (which follow an extreme value distribution) useful for evaluating the significance of alignment scores [35]. We have used a fairly conservative Z-value cutoff (Z_c _= 8.0) [35] to decide the statistical significance of a C-terminal alignment. Selection criteria had to be relaxed for two legitimate selenoproteins, a sulfur transferase in *G. sulfurreducens *(Z-value 7.9) and a coenzyme F420-reducing hydrogenase subunit in *M. maripaludis *(Z-value 4.6).

### Multiple sequence alignment

For each of the selected candidate ORFs (Z_c _≥ 8), a sensitive, iterative PSI-BLAST search was performed using position-specific scoring matrices. The top 10 hits (E ≤ 10^-3^) were used to construct a MSA with ClustalW [36]. Amino acids lining up with the putative selenocysteine residue were examined. Selenocysteines that aligned with two or more cysteine residues were selected for further analysis.

### SECIS element analysis

In accordance with a recent analysis of bacterial SECIS elements [16], a 111 nucleotide long mRNA stretch surrounding the UGA codon position (-10 to +100) was extracted from each of the selected ORFs passing the previous tests in each bacterial organism. The extracted set of RNA sequences for each organism was used to detect a common, single hairpin motif using the rapid, heuristic-based RNAPROFILE program [20]. A test calculation predicted the known SECIS element of the gene encoding FDH from *E. coli *correctly [37] (Figure 3b). The putative SECIS hairpin motifs were manually inspected for consistency.

### Control analysis

To evaluate the performance of the RSA step, we analyzed the *Mycoplasma genitalium *organism that utilizes UGA to code for tryptophan throughout its genome. *M. genitalium *is a small genome with 470 genes [38], the majority of which have a homolog in our database, thus minimizing database effects in our calculation. We applied the RSA method to all the theoretical ORFs with one in-frame TAA (313) or TAG (137) or TGA (780) codon. A self-excluding BLAST database of microbial proteins was used. In *M. genitalium*, over 78% of the TGA cases (91% when a self-included database was used) were identified by the RSA method as recoding events with a Z-value of 8 or higher. These cases aligned overwhelmingly with tryptophan residues in homologs. In contrast, only about 2% to 3% of the ORFs contatining a TAA or TAG stop codon passed the same RSA test.

We also applied the selenoprotein detection scheme to the *Aeropyrum pernix *(BA000002) genome, which does not contain any selenocysteine insertion genes. Out of 26 of 1,288 ORFs with in-frame 'UGA' that were selected by RSA (approximately 2%), none were selected in the subsequent MSA test.

### A web-server for RSA analysis

A web-based service is available for RSA analysis of submitted DNA sequences [29]. The server was designed to analyze an ORF with one in-frame stop codon (UAA, UAG or UGA). A larger, non-redundant BLAST database (to be updated regularly) is used by the web server. The Z-value score and the MSA for the ORF are returned to the user.

### Sensitivity and specificity

Sensitivity = true positive/(true positive + false negative)

Specificity = true positive/(true positive + false positive)

Estimates of true positives, false negatives and false positives were based on predictions performed on the set of organisms whose selenoproteins have been described in the RECODE [21] database (Figure 2a). The number of true positives is taken to be the number of predictions that are already known selenoproteins in the RECODE database. False negatives are those known selenoproteins not predicted by our method. False positives are difficult to estimate. As an extreme estimate, we have taken as an upper bound all those predictions that are not in the known database. The actual false positive rate is probably considerably lower than this estimate.

## Additional data files

The following additional data are available with the online version of this paper. Additional data file 1 is a list of all the genomes analyzed together with the NCBI accession number. Additional data file 2 contains all the predicted recoded proteins from the complete genomes analyzed in this study in FASTA format.

## Supplementary Material

Additional File 1 A list of all the genomes analyzed together with the NCBI accession number Click here for file

Additional File 2 All the predicted recoded proteins from the complete genomes analyzed in this study in FASTA format Click here for file

## Figures and Tables

**Table 1:** A list of predicted selenoproteins encoded by UGA read-through

Accession ID	Organism	Computationally identified selenoproteins* annotated by their homologs
AE000657	*Aquifex aeolicus*	1. gi|12515210|gb|AAG56295.1|AE005358_3 formate dehydrogenase-N, nitrate-inducible, alpha subunit [*Escherichia coli*]
		2. gi|51589698|emb|CAH21328.1| selenide, water dikinase [*Yersinia pseudotuberculosis *IP 32953]
AE017125	*Helicobacter hepaticus*	1.gi|27362035|gb|AAO10941.1|AE016805_198 formate dehydrogenase, alpha subunit [*Vibrio vulnificus *CMCP6]
		2. gi|46914191|emb|CAG20971.1| putative selenophosphate synthase [*Photobacterium profundum*]
AE017143	*Haemophilus ducreyi 35000HP*	1. gi|26108424|gb|AAN80626.1|AE016761_201 selenide, water dikinase [*Escherichia coli *CFT073]
AE004439	*Pasteurella multocida*	1. gi|2983532|gb|AAC07107.1| formate dehydrogenase, alpha subunit [*Aquifex aeolicus *VF5]
		**2. gi|5103639|dbj|BAA79160.1| 194 amino acid long hypothetical protein [*Aeropyrum pernix *K1]**
AE005674	*Shigella flexneri 2a*	**1. gi|12515215|gb|AAG56300.1|AE005358_8 orf; unknown function [*Escherichia coli *O157:H7 EDL933]**
		**2. gi|1788928|gb|AAC75627.1| quinolinate synthetase, B protein; quinolinate synthetase, B protein, catalytic and NAD/flavoprotein subunit [*Escherichia coli *>K12]**
		3. gi|2983532|gb|AAC07107.1| formate dehydrogenase, alpha subunit [*Aquifex aeolicus *VF5]
		4. gi|2983532|gb|AAC07107.1| formate dehydrogenase, alpha subunit [*Aquifex aeolicus *VF5]
		5. gi|3868721|gb|AAD13462.1| selenopolypeptide subunit of formate dehydrogenase H; formate dehydrogenase H, selenopolypeptide subunit [*Escherichia coli *K12]
AE014073	*Shigella flexneri 2a*	1. gi|2983532|gb|AAC07107.1| formate dehydrogenase, alpha subunit [*Aquifex aeolicus *VF5]
		**2. gi|1788928|gb|AAC75627.1| quinolinate synthetase, B protein; quinolinate synthetase, B protein, catalytic and NAD/flavoprotein subunit [*Escherichia coli *K12]**
		3. gi|2983532|gb|AAC07107.1| formate dehydrogenase, alpha subunit [*Aquifex aeolicus *VF5]
		4. gi|3868721|gb|AAD13462.1| selenopolypeptide subunit of formate dehydrogenase H; formate dehydrogenase H, selenopolypeptide subunit [*Escherichia coli *K12]
AE006469	*Sinorhizobium meliloti*	1. gi|2983532|gb|AAC07107.1| formate dehydrogenase, alpha subunit [*Aquifex aeolicus *VF5]
AE008691	*Thermoanaerobacter tengcongensis*	1. gi|41816370|gb|AAS11237.1| glycine reductase complex selenoprotein GrdA [*Treponema denticola *ATCC 35405]
		2. gi|51857693|dbj|BAD41851.1| glycine reductase complex selenoprotein B [*Symbiobacterium thermophilum *IAM 14863]
		3. gi|46914191|emb|CAG20971.1| putative selenophosphate synthase [*Photobacterium profundum*]
AE014075	*Escherichia coli CFT073*	1. gi|2983532|gb|AAC07107.1| formate dehydrogenase, alpha subunit [*Aquifex aeolicus *VF5]
		2. gi|56130341|gb|AAV79847.1| formate dehydrogenase H [*Salmonella enterica *subsp. *enterica serovar Paratyphi A *str. ATCC 9150]
		3. gi|2983532|gb|AAC07107.1| formate dehydrogenase, alpha subunit [*Aquifex aeolicus *VF5]
BA000007	*Escherichia coli O157H7*	1. gi|56130341|gb|AAV79847.1| formate dehydrogenase H [*Salmonella enterica *subsp. *enterica serovar Paratyphi A *str. ATCC 9150]
		2. gi|2983532|gb|AAC07107.1| formate dehydrogenase, alpha subunit [*Aquifex aeolicus *VF5]
		3. gi|2983532|gb|AAC07107.1| formate dehydrogenase, alpha subunit [*Aquifex aeolicus *VF5]
U00096	*Escherichia coli K12*	**1. gi|5105267|dbj|BAA80580.1| 114 amino acid long hypothetical protein [*Aeropyrum pernix *K1]**
		2. gi|2983532|gb|AAC07107.1| formate dehydrogenase, alpha subunit [*Aquifex aeolicus *VF5]
		3. gi|56130341|gb|AAV79847.1| formate dehydrogenase H [*Salmonella enterica *subsp. *enterica serovar Paratyphi A *str. ATCC 9150]
		4. gi|2983532|gb|AAC07107.1| formate dehydrogenase, alpha subunit [*Aquifex aeolicus *VF5]
AE014299	*Shewanella oneidensis*	1. gi|2983532|gb|AAC07107.1| formate dehydrogenase, alpha subunit [*Aquifex aeolicus *VF5]
AE015451	*Pseudomonas putida KT2440*	1. gi|2983532|gb|AAC07107.1| formate dehydrogenase, alpha subunit [*Aquifex aeolicus *VF5]
AE004091	*Pseudomonas aeruginosa*	1. gi|2983532|gb|AAC07107.1| formate dehydrogenase, alpha subunit [*Aquifex aeolicus *VF5]
AE016958	*Mycobacterium avium paratuberculosis*	**1. gi|13880045|gb|AAK44759.1| hypothetical protein MT0536 [*Mycobacterium tuberculosis *CDC1551]**
		2. gi|2983532|gb|AAC07107.1| formate dehydrogenase, alpha subunit [*Aquifex aeolicus *VF5]
AE017042	*Yersinia pestis biovar Mediaevalis*	1. gi|2983532|gb|AAC07107.1| formate dehydrogenase, alpha subunit [*Aquifex aeolicus *VF5]
AE009952	*Yersinia pestis KIM*	1. gi|2983532|gb|AAC07107.1| formate dehydrogenase, alpha subunit [*Aquifex aeolicus *VF5]
AL590842	*Yersinia pestis CO92*	1. gi|2983532|gb|AAC07107.1| formate dehydrogenase, alpha subunit [*Aquifex aeolicus *VF5]
AE017180	*Geobacter sulfurreducens*	1. gi|19918170|gb|AAM07420.1| 4-carboxymuconolactone decarboxylase [*Methanosarcina acetivorans *str. C2A]
		2. gi|21956737|gb|AAM83670.1|AE013608_5 glutaredoxin 3 [*Yersinia pestis *KIM]
		3. gi|37201109|dbj|BAC96933.1| thiol-disulfide isomerase and thioredoxins [*Vibrio vulnificus *YJ016]
		4. gi|2983532|gb|AAC07107.1| formate dehydrogenase, alpha subunit [*Aquifex aeolicus *VF5]
		5. gi|34105000|gb|AAQ61356.1| conserved hypothetical protein [*Chromobacterium violaceum *ATCC 12472]; gi|53758707|gb|AAU92998.1| HesB/YadR/YfhF family protein [*Methylococcus capsulatus *str. Bath];
		6. gi|46914191|emb|CAG20971.1| Putative selenophosphate synthase [*Photobacterium profundum*]
		7. gi|32448022|emb|CAD77542.1| peroxiredoxin [*Pirellula *sp.]
		8. gi|29605647|dbj|BAC69712.1 hypothetical protein [*Streptomyces avermitilis *MA-4680] (SelW)
		9. gi|34482757|emb|CAE09757.1| sulfur transferase precursor [*Wolinella succinogenes*]
AE017226	*Treponema denticola ATCC 35405*	1. gi|51857694|dbj|BAD41852.1| glycine reductase complex selenoprotein A [*Symbiobacterium thermophilum *IAM 14863]
		2. gi|51857693|dbj|BAD41851.1| glycine reductase complex selenoprotein B [*Symbiobacterium thermophilum *IAM 14863]
		3. gi|56380162|dbj|BAD76070.1| glutathione peroxidase [*Geobacillus kaustophilus *HTA426]
		4. gi|51857693|dbj|BAD41851.1| glycine reductase complex selenoprotein B [*Symbiobacterium thermophilum *IAM 14863]
		5. gi|26108424|gb|AAN80626.1|AE016761_201 selenide, water dikinase [*Escherichia coli *CFT073]
		6. gi|52209545|emb|CAH35498.1| thioredoxin 1 [*Burkholderia pseudomallei *K96243]
AL111168	*Campylobacter jejuni*	1. gi|27362035|gb|AAO10941.1|AE016805_198 formate dehydrogenase, alpha subunit [*Vibrio vulnificus *CMCP6]
		2. gi|54018125|dbj|BAD59495.1| hypothetical protein [*Nocardia farcinica *IFM 10152]; (SelW)
AL513382	*Salmonella typhi*	1. gi|3868721|gb|AAD13462.1| selenopolypeptide subunit of formate dehydrogenase H; formate dehydrogenase H, selenopolypeptide subunit [*Escherichia coli *K12]
		2. gi|2983532|gb|AAC07107.1| formate dehydrogenase, alpha subunit [*Aquifex aeolicus *VF5]
AE006468	*Salmonella typhimurium LT2*	1. gi|2983532|gb|AAC07107.1| formate dehydrogenase, alpha subunit [*Aquifex aeolicus *VF5]
		2. gi|2983532|gb|AAC07107.1| formate dehydrogenase, alpha subunit [*Aquifex aeolicus *VF5]
		3. gi|3868721|gb|AAD13462.1| selenopolypeptide subunit of formate dehydrogenase H; formate dehydrogenase H, selenopolypeptide subunit [*Escherichia coli *K12]
BA000016	*Clostridium perfringens*	1. gi|28202985|gb|AAO35429.1| conserved protein [*Clostridium tetani *E88]; gi|20906561|gb|AAM31712.1| HesB protein [*Methanosarcina mazei *Goe1]
		2. gi|46914191|emb|CAG20971.1| putative selenophosphate synthase [*Photobacterium profundum*]
BX470251	*Photorhabdus luminescens*	1. gi|2983532|gb|AAC07107.1| formate dehydrogenase alpha subunit [*Aquifex aeolicus *VF5]
BX571656	*Wolinella succinogenes*	1. gi|27362035|gb|AAO10941.1|AE016805_198 formate dehydrogenase, alpha subunit [*Vibrio vulnificus *CMCP6]
L42023	*Haemophilus influenzae*	1. gi|2983532|gb|AAC07107.1| formate dehydrogenase, alpha subunit [*Aquifex aeolicus *VF5]
		2. gi|26108424|gb|AAN80626.1|AE016761_201 selenide, water dikinase [*Escherichia coli *CFT073]
CR354531	*Photobacterium profundum*	**1. gi|58428447|gb|AAW77484.1| conserved hypothetical protein [*Xanthomonas oryzae *pv. *oryzae *KACC10331]**
CR354532	*Photobacterium profundum*	1. gi|41816370|gb|AAS11237.1| glycine reductase complex selenoprotein GrdA [*Treponema denticola *ATCC 35405]
		2. gi|51589698|emb|CAH21328.1| selenide, water dikinase [*Yersinia pseudotuberculosis *IP 32953]
		3. gi|41816370|gb|AAS11237.1| glycine reductase complex selenoprotein GrdA [*Treponema denticola *ATCC 35405]
		4. gi|41818450|gb|AAS12639.1| glycine reductase complex selenoprotein GrdB2 [*Treponema denticola *ATCC 35405]
AE009439	*Methanopyrus kandleri *(archaea)	1. gi|2622673|gb|AAB86026.1| formate dehydrogenase, alpha subunit homolog [*Methanothermobacter thermautotrophicus*]; gi|2622681|gb|AAB86033.1| tungsten formylmethanofuran dehydrogenase, subunit B [*Methanothermobacter thermautotrophicus*]
		2. gi|57160335|dbj|BAD86265.1| probable formate dehydrogenase, alpha subunit [*Thermococcus kodakaraensis *KOD1]
		3. gi|33566318|emb|CAE37231.1| putative iron-sulfur binding protein [*Bordetella parapertussis*]
		4. gi|44921146|emb|CAF30381.1| heterodisulfide reductase, subunit A [*Methanococcus maripaludis*]
		5. gi|44921142|emb|CAF30377.1| coenzyme F420-non-reducing hydrogenase, subunit delta [*Methanococcus maripaludis*]; gi|2622243|gb|AAB85627.1| methyl viologen-reducing hydrogenase, delta subunit homolog FlpD [*Methanothermobacter thermautotrophicus*]; gi|20904385|gb|AAM29752.1| heterodisulfate reductase, subunit A [*Methanosarcina mazei *Goe1]
		6. gi|45047811|emb|CAF30938.1| coenzyme F420-reducing hydrogenase subunit alpha [*Methanococcus maripaludis*]
		7. gi|39576202|emb|CAE80367.1| selenide, water dikinase [*Bdellovibrio bacteriovorus *HD100]
L77117	*Methanococcus jannaschii *(archaea)	1. gi|44921146|emb|CAF30381.1| heterodisulfide reductase subunit A [*Methanococcus maripaludis*]
		2. gi|45047811|emb|CAF30938.1| coenzyme F420-reducing hydrogenase subunit alpha [*Methanococcus maripaludis*]
		3. gi|50875900|emb|CAG35740.2| methyl-viologen-reducing hydrogenase, delta subunit [*Desulfotalea psychrophila *LSv54]
		4. gi|2622240|gb|AAB85625.1| methyl viologen-reducing hydrogenase, delta subunit [*Methanothermobacter thermautotrophicus*]; gi|44921142|emb|CAF30377.1| coenzyme F420-non-reducing hydrogenase subunit delta [*Methanococcus maripaludis*]
		5. gi|2622673|gb|AAB86026.1| formate dehydrogenase, alpha subunit homolog [*Methanothermobacter thermautotrophicus*]; gi|45048129|emb|CAF31247.1| tungsten containing formylmethanofuran dehydrogenase, subunit B [*Methanococcus maripaludis*] (overlaps with #4)
		6. gi|26108424|gb|AAN80626.1|AE016761_201 selenide, water dikinase [*Escherichia coli *CFT073]
		7. gi|53758707|gb|AAU92998.1| HesB/YadR/YfhF family protein [*Methylococcus capsulatus *str. *Bath*]
		8. gi|45047727|emb|CAF30854.1| formate dehydrogenase, alpha subunit [*Methanococcus maripaludis*]
BX950229	*Methanococcus maripaludis *(archaea)	1. gi|2622673|gb|AAB86026.1| formate dehydrogenase, alpha subunit homolog [*Methanothermobacter thermautotrophicus*]; gi|19886584|gb|AAM01476.1| Formylmethanofuran dehydrogenase subunit B [*Methanopyrus kandleri *AV19]
		2. gi|2622673|gb|AAB86026.1| formate dehydrogenase, alpha subunit homolog [*Methanothermobacter thermautotrophicus*]
		3. gi|2622240|gb|AAB85625.1| methyl viologen-reducing hydrogenase, delta subunit [*Methanothermobacter thermautotrophicus*]; gi|39981962|gb|AAR33424.1| heterodisulfide reductase subunit [*Geobacter sulfurreducens *PCA]
		4. gi|2622673|gb|AAB86026.1| formate dehydrogenase, alpha subunit homolog [*Methanothermobacter thermautotrophicus*]
		5. gi|2622673|gb|AAB86026.1| formate dehydrogenase, alpha subunit homolog [*Methanothermobacter thermautotrophicus*]; gi|19918286|gb|AAM07526.1| formylmethanofuran dehydrogenase, subunit B [*Methanosarcina acetivorans *str. C2A]
		6. gi|19886593|gb|AAM01482.1| Heterodisulfide reductase, subunit A, polyferredoxin [*Methanopyrus kandleri *AV19]

Organism names, National Center for Biotechnology Information accession numbers for the genomes and the top PSI-BLAST hit(s) from our database are shown. Seven novel candidate selenoproteins are shown in bold type. *Each entry corresponds to a computationally identified read-through protein in the organism indicated to the left. FASTA files for these recoded protein sequences are provided in the Additional file 2. For each recoded protein, the GI number and the functional annotation for a homologous protein are given.

**Table 2:** Methyltransferases predicted to encode pyrrolysine by UAG read-through in a set of methanogenic archaea

Organism	Computationally identified pyrrolysine-proteins* annotated by their homologs
*Methanosarcina acetivorans *(AE010299)	1. gi|56678713|gb|AAV95379.1| trimethylamine methyltransferase family protein [*Silicibacter pomeroyi *DSS-3]
	2. gi|14247242|dbj|BAB57633.1| menaquinone biosynthesis methyltransferase [*Staphylococcus aureus *subsp. *Aureus *Mu50]
	3. gi|36785418|emb|CAE14364.1| protein methyltranferase [*Photorhabdus luminescens *subsp. *laumondii *TTO1]
	4. gi|56679325|gb|AAV95991.1| trimethylamine methyltransferase family protein [*Silicibacter pomeroyi *DSS-3]
	5. i|20904823|gb|AAM30145.1| SAM-dependent methyltransferases [*Methanosarcina mazei *Goe1]
	6. gi|56312282|emb|CAI06927.1| predicted methyltransferase [*Azoarcus *sp. EbN1]
	7. gi|45047608|emb|CAF30735.1| generic methyltransferase [*Methanococcus maripaludis*]
	8. gi|20905508|gb|AAM30766.1| methylcobalamin: Coenzyme M methyltransferase [*Methanosarcina mazei *Goe1]
	9. Predicted ORF monomethylamine methyltransferase [*Methanosarcina mazei *Goe1]^†^
	10. Predicted ORF monomethylamine methyltransferase [*Methanosarcina mazei *Goe1]^†^
	11. Predicted ORF dimethylamine methyltransferase [*Methanosarcina mazei *Goe1]^†^
	12. Predicted ORF dimethylamine methyltransferase [*Methanosarcina mazei *Goe1]^†^
	13. Predicted ORF dimethylamine methyltransferase [*Methanosarcina mazei *Goe1]^†^
*Methanosarcina mazei *(AE008384)	1. gi|19914316|gb|AAM03972.1| trimethylamine methyltransferase [*Methanosarcina acetivorans *str. C2A]
	2. gi|19914320|gb|AAM03976.1| dimethylamine methyltransferase [*Methanosarcina acetivorans *str. C2A]
	3. gi|19914753|gb|AAM04365.1| trimethylamine methyltransferase [*Methanosarcina acetivorans *str. C2A]
	4. gi|19913899|gb|AAM03597.1| monomethylamine methyltransferase [*Methanosarcina acetivorans *str. C2A]
	5. gi|19914755|gb|AAM04366.1| dimethylamine methyltransferase [*Methanosarcina acetivorans *str. C2A]
	6. gi|19914320|gb|AAM03976.1| dimethylamine methyltransferase [*Methanosarcina acetivorans *str. C2A]
	7. gi|19913899|gb|AAM03597.1| monomethylamine methyltransferase [*Methanosarcina acetivorans *str. C2A]
*Methanosarcina barkeri *(draft genome)	1. gi|19914320|gb|AAM03976.1| dimethylamine methyltransferase [*Methanosarcina acetivorans *str. C2A]
	2. gi|19913899|gb|AAM03597.1| monomethylamine methyltransferase [*Methanosarcina acetivorans *str. C2A]
	3. gi|19914316|gb|AAM03972.1| trimethylamine methyltransferase [*Methanosarcina acetivorans *str. C2A]
	4. gi|19914320|gb|AAM03976.1| dimethylamine methyltransferase [*Methanosarcina acetivorans *str. C2A]
	5. gi|19914334|gb|AAM03988.1| protein-L-isoaspartate (D-aspartate) O-methyltransferase [*Methanosarcina acetivorans *str. C2A]
	6. gi|19913899|gb|AAM03597.1| monomethylamine methyltransferase [*Methanosarcina acetivorans *str. C2A]
	7. gi|19913899|gb|AAM03597.1| monomethylamine methyltransferase [*Methanosarcina acetivorans *str. C2A]
*Methanococcoides burtonii *(draft genome)	1. gi|19914320|gb|AAM03976.1| dimethylamine methyltransferase [*Methanosarcina acetivorans *str. C2A]
	2. gi|19914753|gb|AAM04365.1| trimethylamine methyltransferase [*Methanosarcina acetivorans *str. C2A]
	3. gi|5458504|emb|CAB49992.1| methlytransferase, putative [*Pyrococcus abyssi*]
	4. gi|5458504|emb|CAB49992.1| methlytransferase, putative [*Pyrococcus abyssi*] (overlaps with #3)
	5. gi|19914320|gb|AAM03976.1| dimethylamine methyltransferase [*Methanosarcina acetivorans *str. C2A]
	6. gi|19914753|gb|AAM04365.1| trimethylamine methyltransferase [*Methanosarcina acetivorans *str. C2A]
	7. gi|19913899|gb|AAM03597.1| monomethylamine methyltransferase [*Methanosarcina acetivorans *str. C2A

*Each entry corresponds to a computationally identified read-through protein in the organism indicated to the left. FASTA files for these recoded protein sequences are provided in the Additional data files. For each recoded protein, the GI number and the functional annotation for a homologous protein are given. ^†^These open reading frames (ORFs) in *M. acitovorans *were predicted during a repeat search using a BLAST database containing putative methylamine methyltransferase ORFs in *M. mazei *as identified by our method. Although the *M. acitovorans *genome was annotated for several pyrrolysine-containing methylamine methyltranferases, this was not the case with the *M. mazei *genome. Thus, several methyltransferases that are specific to these methanosarcina species could not be detected in our original calculation due to the lack of read-through homologs. Such repeat searches were not performed for the two unfinished genomes.
