# Supplementary material for: A computational method to predict genetically encoded rare amino acids in proteins
Source: Genome Biol. 2005 Aug 31;6(9):R79. doi: 10.1186/gb-2005-6-9-r79 (PMC1242214; doi:10.1186/gb-2005-6-9-r79)
Supplement: Additional File 1 — A list of all the genomes analyzed together with the NCBI accession number [file gb-2005-6-9-r79-S1.doc]

**Supplementary material 1**

List of organisms with SID genes that are used for selenoprotein search

| 1. AE000657 | *Aquifex aeolicus* |
| --- | --- |
| 2. AE016877 | *Bacillus cereus ATCC14579* |
| 3. AL111168 | *Campylobacter jejuni* |
| 4. BA000016 | *Clostridium perfringens* |
| 5. AE014075 | *Escherichia coli CFT073* |
| 6. U00096 | *Escherichia coli K12* |
| 7. BA000007 | *Escherichia coli O157H7* |
| 8. AE005174 | *Escherichia coli O157H7 EDL* |
| 9. AE017180 | *Geobacter sulfurreducens* |
| 10. AE017143 | *Haemophilus ducreyi 35000HP* |
| 11. L42023 | *Haemophilus influenzae* |
| 12. AE017125 | *Helicobacter hepaticus* |
| 13. AE000511 | *Helicobacter pylori 26695* |
| 14. AE001439 | *Helicobacter pylori J99* |
| 15. L77117 | *Methanococcus jannaschii* |
| 16. BX950229 | *Methanococcus maripaludis* |
| 17. AE009439 | *Methanopyrus kandleri* |
| 18. AE016958 | *Mycobacterium avium paratuberculosis* |
| 19. BA000028 | *Oceanobacillus iheyensis* |
| 20. AE004439 | *Pasteurella multocida* |
| 21. BX470251 | *Photorhabdus luminescens* |
| 22. AE004091 | *Pseudomonas aeruginosa* |
| 23. AE015451 | *Pseudomonas putida KT2440* |
| 24. AL513382 | *Salmonella typhi* |
| 25. AE006468 | *Salmonella typhimurium LT2* |
| 26. AE014299 | *Shewanella oneidensis* |
| 27. AE005674 | *Shigella flexneri 2a* |
| 28. AE014073 | *Shigella flexneri 2a* |
| 29. AE006469 | *Sinorhizobium meliloti* |
| 30. AE008691 | *Thermoanaerobacter tengcongensis* |
| 31. AE017226 | *Treponema denticola ATCC 35405* |
| 32. BX571656 | *Wolinella succinogenes* |
| 33. AL590842 | *Yersinia pestis CO92* |
| 34. AE009952 | *Yersinia pestis KIM* |
| 35. AE017042 | *Yersinia pestis biovar Mediaevails* |
| 36. CR354531 | *Photobacterium profundum* |
| 37. CR354532 | *Photobacterium profundum* |
