# Supplementary material for: A computational method to predict genetically encoded rare amino acids in proteins
Source: Genome Biol. 2005 Aug 31;6(9):R79. doi: 10.1186/gb-2005-6-9-r79 (PMC1242214; doi:10.1186/gb-2005-6-9-r79)
Supplement: Additional File 2 — All the predicted recoded proteins from the complete genomes analyzed in this study in FASTA format [file gb-2005-6-9-r79-S2.htm]

Master list: 


**Master list:� Putative selenoproteins (92 ORFs).**

**Please note that the start position may not be
correct.**

 

|  |  |  |  |  |
| --- | --- | --- | --- | --- |
| **#** | **Accession ID** | ***Organism*** | Functional  Assignment | **Sequence** |
| 1 | AE000657 | *Aquifex aeolicus* | gi|12515210|gb|AAG56295.1|AE005358\_3 formate dehydrogenase-N, nitrate-inducible, alpha subunit [Escherichi | >ORF 226 and organism AE000657.seq  VNYMDISRRGFLKLSVGSVGAGILGGLGFDLTPAYARVRDLKITKAKVTKSICPYCSVSCGILAYSLSDG  AMNVKERIIHVEGNPDDPINRGTLCPKGATLRDFVNAPDRLTKPLYRPAGSTEWKEISWDEAIEKFARWV  KDTRDRTFIHKDKAGRVVNRCDSIVWAVGSPLGNEEGWLMVKIGIALGLSARETQATI**U**HAPTVASLAPT  FGRGAMTNNWVDISNSDLVFVMGGNPAENHPCGFKWAIKAREKRGAKIICIDPRFNRTAAVADIFVQIRP  GTDIAFLGGLINYVLQNEKYQKEYVRLHTTGPFIVREDFGFKDGLFTGYDPKTRSYDTTTWDYEFDPATG  YPKMDPEMKHPRCVLNILKEHYSRYTPEVVSQICGCSKEDFLRVAEEVAKCGAPNKFMTILYALGWTHHS  YGTQLIRTACMLQLLLGNIGCPGGGINALRGHSNVQGMTDLAGQNKNLPTYIKPPKPEEQTLAQHLKNRT  PRKLHPTSLNYWANYPKFFISFLKCMWGDAATPENDFAYDYLYKPEGGYNSWDKFIDDMYKGKIEGVVTA  ALNFLNNTPNAKKTVRALKNLKWMVVMDPFMIETAQFWKAEGLDPKEVKTEILVLPTAVFLEKEGSFTNS  ARWVKWKYKATDPPGDAKDEFWIFGRFFMKLKEFYEKEGGAFPEPILNLVWPYKNPYYPTAEEILTEING  YYTRDVDGHKKGERVRLFTDLRDDGSTACGGWLYCGVFPPEGNLAKRTDLSDPLGLGTYPNYAWNWPANR  RVLYNRASCDEKGRPWDPERPLLRWDPERDMWVGDIPDYPATAPPEKGIGAFIMLPEGKGRLFAAKSYVT  FKDGPLPEHYEPYESPVTNILHPNVPHNPVAKVYKSDLDLLGTPDKFPHVATTYRLTEHYHFWTKHLYGP  SLLAPVMFIEIPEELAKEKGIQNGDLVRVSTARASIEAIALVTKRIKPLKVAGKTVYTIGIPIHWGFEGL  VKGAITNFITPNVWDPNSRTPEFKGFLANIEKVKT |
| 2 |  |  | gi|51589698|emb|CAH21328.1| selenide, water dikinase [Yersinia pseudotuberculosi | >ORF 2691 and organism AE000657.seq  MVELLKLVRSSG**U**AAKVGPGDLQEILKGFNIYTDESTLVSIGDDAGVYEHNGIIWVYTVDIITPVVNDPY  LWGAISTANALSDVYAMGGIPVNALAISCFNNCELDIEIFREVIRGALDKLREAKTVLLGGHTIDDKEPK  FGLSVAGICPEGKYITQSGAQVGQLLILTKPIGTGILIKGLKEGILKEEDINEAIENMLALNDKARNLML  SLDATACTDVTGFGLLGHAWNICKNSNIGARIFFEKVPYYQLSENLVKKKIYPKGAIENLNFVKNYLKSN  LDNWKLILLSDPVTSGGLLFTINKEKLEKIDETAKELEVNYWIIGETIAENVLEVL |
| 3 | AE017125 | *Helicobacter hepaticus* | gi|27362035|gb|AAO10941.1|AE016805\_198 Formate dehydrogenase, alpha subunit [Vibrio vulnificus CMCP6] | >ORF 1250 and organism AE017125.seq  VKMSEVNDRRNARRSFLKLSALASVAGVSSALGNEGEKVLRKASEAELKEKYPQSQKIKTICTHCSVGCG  VIAEVQDGVWVRQEVAQDHPISQGGHCCKGADLIDRARSETRLRYPLQKQNGQWTRLKYDEAMDKIATQL  KQIREESGPDAVMFLGSAKCSNEQSYYIRKFAAFFGTNNIDHCARV**U**HSPTVAGVANTFGYGGMTNHLGD  MMFSKYILVIGANPAVNHPVSMVHILRAKEQGAKLVCIDPRFTKTAAKCDEFHRIRSGTDIAFAYGLLNH  IIAKKLYDEQYLKERVYGYEDIIKEAQKFSPEVAADICGIPADEIRHIAEEMAAAKPASLIWNQGLTQHT  IGTSNTRIMPILQMFLGNIGKNGGGVNILRGHDNVQGASDMNNLADSLPGYYGLGEPAWRHFCKHWGVEY  EWMLGRFKDAEMMGKTGFAHSTWKFGVLDEENAANNGGTKLRALVVIGSGMTTVSLLDLQKKAMDMLDLV  VFVDPYVNDLAIYSDRSDNLFMLPAASQMETSGSVAATNRSYQWRSKVMEPLFECRPDEEFLFGLAERLG  FLKELQWRLYDIAKSKGREQFVWPEDATTELTQSIRSIGLQGMSPERLKAHQENWHLFDKVTLEGTGEFK  GDYYGLPWPCWSDKHPGTPVMYNDTIPVMRGGMGFRVNWGVTSPDGQSMLTNRSLPNAKHIGGYAPVTAE  NAESLGISLSSEEKNAIEGSTFALGIGNNILVEKALEAGLCPYGNGKARANVWNWYDKIPLHREPLHSVR  GDLVDKYPSFPDKPNLFRANVKYISRQKEKDWVKEFPINMLSGRLVAHMGTGAETRSAKYLAEVEGEMFV  EIHPNKAAELKVKNGDLVWIYGTNGCKILVPAKLSVRVDENSIWLPQNFSGMDQGESRLDKYPEGTKPYA  IGESANMISSYGYDYNSACPETKCGLCRIEKA |
| 4 |  |  | gi|46914191|emb|CAG20971.1| Putative selenophosphate synthase [Photobacterium profundum] | >ORF 966 and organism AE017125.seq  MADYQLTKHIQCAG**U**AAKVGLSDLAQISSQLTQKPNSLLIAGFESNEDCGAMLYAPNDEYAMLSSVDFIT  PVVDDPYLYGQIAAANALSDIFAMGGEVKSALNLLMWDNTHFDSAVANAILKGGLNKITESNALLLGGHT  IKDKEQKYGLAVNGIAHKNRLWRNHTGYIGDMLVLTKPLGSGILTTAIKAQMFSQTTEVTQSMAMLNLYA  ARIAQNYEIHACTDITGFGLIGHAFEMCGGIKNQNEKSILFYTKQIPLFDKTESFSQMGIVPGGSYENKK  ALQSQVQIQCTLEDDIFYYDAQTSGGLLFALPFNQAKLFVDELHKAGIIHANVIGEIIPKTETSIVLG |
| 5 | AE017143 | *Haemophilus ducreyi 35000HP* | gi|26108424|gb|AAN80626.1|AE016761\_201 Selenide,water dikinase [Escherichia coli CFT073] | >ORF 566 and organism AE017143.seq  LLCKSNLGGKVSDNIRLTQFSHGAG**U**GCKISPKVLGTILQSELDKFVDPKLLVGNETADDAAVYDIGNGL  AIISTTDFFMPIVDDPFDFGRIAATNALSDIFAMGGKPLMAIAILGFPIDKLPAEVAQKIIEGGRFACQQ  AGIVLAGGHSIHSMEPIFGLAVTGMAAIEHIKRNASATAGCELFLTKPLGIGILTTAEKRGLLTLPHQHL  VRDLMCQLNTIGTLLAPLPEMTAMTDITGFGLLGHLSEICQASNVRAEINSHAVKVIDGVEEYVEQGMIP  GGTQRNFESYANLVSPLSDRQKAILCDQQTSGGLLIAVEPQAVEKIQQIAQQIGSMLFHIGKLFDRAPDK  ALIEVN |
| 6 | AE004439 | *Pasteurella multocida* | gi|2983532|gb|AAC07107.1| formate dehydrogenase alpha subunit [Aquifex aeolicus VF5] | >ORF 1602 and organism AE004439.seq  MQVSRRKFFKICAGGMAGTSAAMLGFAPTEALAAPRNYKLLRAKETRNTCTYCAVGCGMLLYSLGDGSKN  SKGKLFHIEGDPDHPVSRGALCPKGAGALDYVNSDRRVKYPEVREAGSKEWKRISWHEAIERIARHIKDD  RDANFVEKNDAGEPVNRWMTAGFLAGSACSNETGILTQKFVRSLGIIFTDNQASI**U**HGPTVASLAPSFGR  GAMTNHWVDIKNADLVIVMGGNAAEAHPVGFRWAIEAKKQNGAKLMVVDPRFNRTAAVADIYMPLRPGTD  IAFLSGVIRYLLKNDKIQHEYVKHYTNATFLVNENFKFEDGLFSGYDEATRKYDRSTWAYQFDENGQPKR  DMDMQDPRCVINMLREHVERYTPEMVERITGTPQKDFQIFCEEIAKTSAPDKAATFLYALGWTQHTVGSQ  NIRTMAMIQLLLGNIGVSGGGVNALRGHSNVQGITDLGLFPNRLPAYIPLPTEADKSLQSFLDRITPKTM  MNDQVNYWKNTPKFMVSMLKSFYGDKGTQDNEFGYHYLPKLPKGGTDQFRYIEDMYNGKVNGFFCQGMNP  VASYANSQKIIKALSKLKYLVIFDPLITDTSEFWKNYGEFNDVKTEEIQTEVFRLPTTCFVEEDGSIANS  GRWLQWHWKGAEPPGEAKTDGEILSELRAELIHLYKTEGGKAPLEPLEAMSWDYANPLEPKAEEVAKENN  GYALEDIKDADGNIILKKGQLLSSFAQMRDDGTTSGACWIYTGQWTEKGNQMANRDNADPSNLGNTLGWA  FAWPLNRRILYNRAGADLAGNPFNPKRQLIKWNGKNWNYVDVADYGTAPPNSPVMPFIMQPEGVSGLFVR  ERMADGPFPEHYEPMETPIGTNPLHPNVVSSPVARILASDKEDLGTSADFPYVGTTYRLTEHFHYWTKNV  LLNVIAQPEQFVEIGEALAAEKGIKHGDIVKVSSKRGYIKAVAVVTKRIRALVSDGKPIHTVGIPIHWGF  AATTGAKKGFFANNLTVRTGDANTQTPESKCMLVNIEKVGA |
| 7 |  |  | gi|5103639|dbj|BAA79160.1| 194aa long hypothetical protein [Aeropyrum pernix K1] | >ORF 1862 and organism AE004439.seq  VVACTMALANFSGCSDLKIPEPTKTPSAPNSIIKAASAGVATPPAAKFTTGNLPNALICNSNSKGALRFL  ASVMSSSWFMATTLRICALTLRI**U**RTASTILPVPGSPLVRNIEAPSPIRRNASPKSRQPHTNGTVNSHFN  R |
| 8 | AE005674 | *Shigella flexneri 2a* | gi|12515215|gb|AAG56300.1|AE005358\_8 orf; Unknown function [Escherichia coli O157:H7 EDL933] | >ORF 5415 and organism AE005674.seq  MITDLILHNHPRMKTITLNDNHIAHLNAKNTTKLEYLNLSNNNLLPTNDIDQLISSKHLWHVLVNGINND  PLAQMQYWTAVRNIIDDTNEVTIDLSGLNLTTQPPGLQNFTSINLDNNQLTHFDATNYDRLVKLSLNSNA  LESINFPQGRNVSITHISMNNNALRNIDIDRLSSVTYFSAAHNQLEFVQLESCEWLQYLNLSHNQLTDIV  AGNKNELLLLDLSHNKLTSLHNVLFPNLNTLLINNNLLSEIKIFYSNFRNVQTLNAANNQLKYINLDFLT  YLPSIKSLRLDNNKITHIDTNNTSDIGTLFPIIKQSKNLNFLNVSGKNN**U**PTMQLMLFNLFSPALKLNTG  LAILSPGAFEVHSDGIDADNELFHYTIKKAYTPYNIHTYKTEEVVNQRNIKVKNMTLG |
| 9 | � | *�* | gi|1788928|gb|AAC75627.1| quinolinate synthetase, B protein; quinolinate synthetase, B protein, catalytic and NAD/flavoprotein subunit [Escherichia coli K12] | >ORF 5885 and organism AE005674.seq  MNTLPEHSCDVLIIGSGAAGLSLALRLADQHQVIVLSKGPVTEGSTFYAQGGIAAVFDETDSIDSHVEDT  LIAGAGICDHHAVEFVASNARSCVPWLIDQGVLFDTHVQPNGEESYHLTREGGHSHRRILHAADATGREV  QSTLVSKAQNHPNIRVLERSNAVDLIVSDKIGLPGTRRVVGAWVWNRNKETVETCHAKAVVLATGGASKV  YQYTTNPDISSGDGIAMAWRAGCRVANLEFNQFHPTALYHPQARNFLLTEALRGEGAYLKRPDGTRFMPD  FDERGELAPRDIVARAIDHEMKRLGADCMFLDISHKPADFIRQHFPMIYEKLLGLGIDLTQEPVPIVPAA  HYT**U**GGVMVDDHGRTDVEGLYAIGEVSYTGLHGANRMASNSLLECLVYGWSAAEDITRRMPYAHGVSTLP  PWDESRVENPDERVVIQHNWHELRLFMWDYVGIVRTTKRLERALRRITMLQQEIDEYYAHFRVSNNLLEL  RNLVQVAELIVRCAMMRKESRGLHFTLDYPELLTHSGPSILSPGNHYINR |
| 10 |  |  | gi|2983532|gb|AAC07107.1| formate dehydrogenase alpha subunit [Aquifex aeolicus VF5] | >ORF 7173 and organism AE005674.seq  MQVSRRQFFKICAGGMAGTTAAALGFAPSVALAETRQYKLLRTRETRNTCTYCSVGCGLLMYSLGDGAKN  AKASIFHIEGDPDHPVNRGALCPKGAGLVDFIHSESRLKFPEYRAPGSDKWQQISWEEAFDRIAKLMKED  RDANYIAQNAEGVTVNRWLSTGMLCASASSNETGYLTQKFSRALGMLAVDNQARV**U**HGPTVASLAPTFGR  GAMTNHWVDIKNANLVVVMGGNAAEAHPVGFRWAMEAKIHNGAKLIVIDPRFTRTAAVADYYAPIRSGTD  IAFLSGVLLYLLNNEKFNHEYTEAYTNASLIVREDYGFEDGLFTGYDAEKRKYDKSSWTYELDENGFAKR  DTTLQHPRCVWNLLKQHVSRYTPDVVENICGTPKDAFLKVCEYIAETSAHDKTASFLYALGWTQHSVGAQ  NIRTMAMIQLLLGNMGMAGGGVNALRGHSNIQGLTDLGLLSQSLPGYMTLPSEKQTDLQTYLTANTPKPL  LEGQVNYWGNYPKFFVSMMKAFFGDKATAENSWGFDWLPKWDKGYDVLQYFEMMKEGKVNGYICQGFNPV  ASFPNKNKVIGCLSKLKFLVTIDPLNTETSNFWQNHGELNEVDSSKIQTEVFRLPSTCFAEENGSIVNSG  RWLQWHWKGADAPGIALTDGEILSGIFLRLRKMYAEQGGANPDQVLNMTWNYAIPHEPKSEEVAMESNGK  ALADITDPATGAVIVKKGQQLSSFAQLRDDGTTSCGCWIFAGSWTPEGNQMARRDNADPSGLGNTLGWAW  AWPLNRRILYNRASADPQGNPWDPKRQLLKWDGTKWTGWDIPDYSAAPPGSGVGPFIMQQEGMGRLFALD  KMAEGPFPEHYEPFETPLGTNPLHPNVISNPAARIFKDDAEALGKADKFPYVGTTYRLTEHFHYWTKHAL  LNAILQPEQFVEIGESLANKLGIAQGDTVKVSSNRGYIKAKAVVTKRIRTLKANGKDIDTIGIPIHWGYE  GVAKKGFIANTLTPFVGDANTQTPEFKSFLVNVEKV |
| 11 |  |  | gi|2983532|gb|AAC07107.1| formate dehydrogenase alpha subunit [Aquifex aeolicus VF5] | >ORF 8363 and organism AE005674.seq  MDVSRRQFFKICAGGMAGTTVAALGFAPKQALAQARNYKLLRAKEIRNTCTYCSVGCGLLMYSLGDGAKN  AREAIYHIEGDPDHPVSRGALCPKGAGLLDYVNSENRLRYPKYRAPGSDKWQRISWEEAFSRIAKLMKAD  RDANFIEKNEQGVTVNRWLSTGMLCASGASNETGMLTQKFARSLGMLAVDNQARV**U**HGPTVASLAPTFGR  GAMTNHWVDIKNANVVMVMGGNAAEAHPVGFRWAMEAKNNNDATLIVVDPRFTRTASVADIYAPIRSGTD  ITFLSGILRYLIENNKINAEYVKHYTNASLLVRDDFAFEDGLFSGYDAEKRQYDKSSWNYQFDENGYAKR  DETLTHPRCVWNLLKAHVSRYTPDVVENICGTPKADFLKVCEVLASTSAPDRTTTFLYALGWTQHTVGAQ  NIRTMAMIQLLLGNMGMAGGGVNALRGHSNIQGLTDLGLLSTSLPGYLTLPSEKQVDLQSYLEANTPKAT  LADQVNYWSNYPKFFVSLMKSFYGDAAQKENNWGYDWLPKWDQTYDVIKYFNMMDEGKVTGYFCQGFNPV  ASFPDKNKVVSCLSKLKYMVVIDPLVTETSTFWQNHGESNDVDPASIQTEVFRLPSTCFAEEDGSIANSG  RWLQWHWKGQDAPGEARNDGEILAGIYHHLRELYQAEGGKGVEPLMKMSWNYKQPHEPQSDEVAKENNGY  ALEDLYDANGVLIAKKGQLLSSFAHLRDDGTTASSCWIYTGSWTEQGNQMANRDNSDPSGLGNTLGWAWA  WPLNRRVLYNRASADINGKPWDPKRMLIQWNGSKWTGNDIPDFGNAAPGTPTGPFIMQPEGMGRLFAINK  MAEGPFPEHYEPIETPLGTNPLHPNVVSNPVVRLYEQDALRMGKKEQFPYVGTTYRLTEHFHTWTKHALL  NAIAQPEQFVEISETQAAAKGINNGDRVTVSSKRGFIRAVAVVTRRLKPLNVNGQQVETVGIPIHWGFEG  VARKGYIANTLTPNVGDANSQTPEYKAFLVNIEKA |
| 12 |  |  | gi|3868721|gb|AAD13462.1| selenopolypeptide subunit of formate dehydrogenase H; formate dehydrogenase H, selenopolypeptide subunit [Escherichia coli K12] | >ORF 9455 and organism AE005674.seq  MQKFARAVIGTNNVDCCARV**U**HGPSVAGLHQSVGNGAMSNAINEIDNTDLVFVFGYNPADSHPIVANHVI  NAKRNGAKIIVCDPRKIETARIADMHIALKNGSNIALLNAMGHVIIEENLYDKAFVASRTEGFEEYRKIV  EGYTPESVEDITGVSASEIRQAARMYAQAKSAAILWGMGVTQFYQGVETVRSLTSLAMLTSNLGKPHAGV  NPVRGQNNVQGACDMGALPDTYPGYQYVKDPANREKFAKAWGVESLPAHTGYRISELPHRAAHGEVRAAY  IMGEDPLQTDAELSAVRKAFEDLELVIVQDIFITKTASAADVILPSTSWGEHEGVFTAADRGFQRFFKAV  EPKWDLKTDWQIISEIATRMGYPMHYNNTQEIWDELRHLCPDFYGATYEKMGELGFIQWPCRDTSDADQG  TSYLFKEKFDTPNGLAQFFTCDWVAPIDKLTDEYPMVLSTVREVGHYSCRSMTGNCAALAALADEPGYAQ  INTEDAKRLGIEDEALV |
| 13 | AE014073 | *Shigella flexneri 2a* | gi|2983532|gb|AAC07107.1| formate dehydrogenase alpha subunit [Aquifex aeolicus VF5] | >ORF 10531 and organism AE014073.seq  MDVSRRQFFKICAGGMAGTTVAALGFAPKQALAQARNYKLLRAKEIRNTCTYCSVGCGLLMYSLGDGAKN  AREAIYHIEGDPDHPVSRGALCPKGAGLLDYVNSENRLRYPKYRAPGSDKWQRISWEEAFSRIAKLMKAD  RDANFIEKNEQGVTVNRWLSTGMLCASGASNETGMLTQKFARSLGMLAVDNQARV**U**HGPTVASLAPTFGR  GAMTNHWVDIKNANVVMVMGGNAAEAHPVGFRWAMEAKNNNDATLIVVDPRFTRTASVADIYAPIRSGTD  ITFLSGILRYLIENNKINAEYVKHYTNASLLVRDDFAFEDGLFSGYDAEKRQYDKSSWNYQFDENGYAKR  DETLTHPRCVWNLLKAHVSRYTPDVVENICGTPKADFLKVCEVLASTSAPDRTTTFLYALGWTQHTVGAQ  NIRTMAMIQLLLGNMGMAGGGVNALRGHSNIQGLTDLGLLSTSLPGYLTLPSEKQVDLQSYLEANTPKAT  LADQVNYWSNYPKFFVSLMKSFYGDAAQKENNWGYDWLPKWDQTYDVIKYFNMMDEGKVTGYFCQGFNPV  ASFPDKNKVVSCLSKLKYMVVIDPLVTETSTFWQNHGESNDVDPASIQTEVFRLPSTCFAEEDGSIANSG  RWLQWHWKGQDAPGEARNDGEILAGIYHHLRELYQAEGGKGVEPLMKMSWNYKQPHEPQSDEVAKENNGY  ALEDLYDANGVLIAKKGQLLSSFAHLRDDGTTASSCWIYTGSWTEQGNQMANRDNSDPSGLGNTLGWAWA  WPLNRRVLYNRASADINGKPWDPKRMLIQWNGSKWTGNDIPDFGNAAPGTPTGPFIMQPEGMGRLFAINK  MAEGPFPEHYEPIETPLGTNPLHPNVVSNPVVRLYEQDALRMGKKEQFPYVGTTYRLTEHFHTWTKHALL  NAIAQPEQFVEISETQAAAKGINNGDRVTVSSKRGFIRAVAVVTRRLKPLNVNGQQVETVGIPIHWGFEG  VARKGYIANTLTPNVGDANSQTPEYKAFLVNIEKA |
| 14 |  |  | gi|1788928|gb|AAC75627.1| quinolinate synthetase, B protein; quinolinate synthetase, B protein, catalytic and NAD/flavoprotein subunit [Escherichia coli K12] | >ORF 3592 and organism AE014073.seq  MNTLPEHSCDVLIIGSGAAGLSLALRLADQHQVIVLSKGPVTEGSTFYAQGGIAAVFDETDSIDSHVEDT  LIAGAGICDHHAVEFVASNARSCVPWLIDQGVLFDTHVQPNGEESYHLTREGGHSHRRILHAADATGREV  QSTLVSKAQNHPNIRVLERSNAVDLIVSDKIGLPGTRRVVGAWVWNRNKETVETCHAKAVVLATGGASKV  YQYTTNPDISSGDGIAMAWRAGCRVANLEFNQFHPTALYHPQARNFLLTEALRGEGAYLKRPDGTRFMPD  FDERGELAPRDIVARAIDHEMKRLGADCMFLDISHKPADFIRQHFPMIYEKLLGLGIDLTQEPVPIVPAA  HYT**U**GGVMVDDHGRTDVEGLYAIGEVSYTGLHGANRMASNSLLECLVYGWSAAEDITRRMPYAHGVSTLP  PWDESRVENPDERVVIQHNWHELRLFMWDYVGIVRTTKRLERALRRITMLQQEIDEYYAHFRVSNNLLEL  RNLVQVAELIVRCAMMRKESRGLHFTLDYPELLTHSGPSILSPGNHYINR |
| 15 |  |  | gi|2983532|gb|AAC07107.1| formate dehydrogenase alpha subunit [Aquifex aeolicus VF5] | >ORF 4093 and organism AE014073.seq  MQVSRRQFFKICAGGMAGTTAAALGFAPSVALAETRQYKLLRTRETRNTCTYCSVGCGLLMYSLGDGAKN  AKASIFHIEGDPDHPVNRGALCPKGAGLVDFIHSESRLKFPEYRAPGSDKWQQISWEEAFDRIAKLMKED  RDANYIAQNAEGVTVNRWLSTGMLCASASSNETGYLTQKFSRALGMLAVDNQARV**U**HGPTVASLAPTFGR  GAMTNHWVDIKNANLVVVMGGNAAEAHPVGFRWAMEAKIHNGAKLIVIDPRFTRTAAVADYYAPIRSGTD  IAFLSGVLLYLLNNEKFNHEYTEAYTNASLIVREDYGFEDGLFTGYDAEKRKYDKSSWTYELDENGFAKR  DTTLQHPRCVWNLLKQHVSRYTPDVVENICGTPKDAFLKVCEYIAETSAHDKTASFLYALGWTQHSVGAQ  NIRTMAMIQLLLGNMGMAGGGVNALRGHSNIQGLTDLGLLSQSLPGYMTLPSEKQTDLQTYLTANTPKPL  LEGQVNYWGNYPKFFVSMMKAFFGDKATAENSWGFDWLPKWDKGYDVLQYFEMMKEGKVNGYICQGFNPV  ASFPNKNKVIGCLSKLKFLVTIDPLNTETSNFWQNHGELNEVDSSKIQTEVFRLPSTCFAEENGSIVNSG  RWLQWHWKGADAPGIALTDGEILSGIFLRLRKMYAEQGGANPDQVLNMTWNYAIPHEPKSEEVAMESNGK  ALADITDPATGAVIVKKGQQLSSFAQLRDDGTTSCGCWIFAGSWTPEGNQMARRDNADPSGLGNTLGWAW  AWPLNRRILYNRASADPQGNPWDPKRQLLKWDGTKWTGWDIPDYSAAPPGSGVGPFIMQQEGMGRLFALD  KMAEGPFPEHYEPFETPLGTNPLHPNVISNPAARIFKDDAEALGKADKFPYVGTTYRLTEHFHYWTKHAL  LNAILQPEQFVEIGESLANKLGIAQGDTVKVSSNRGYIKAKAVVTKRIRTLKANGKDIDTIGIPIHWGYE  GVAKKGFIANTLTPFVGDANTQTPEFKSFLVNVEKV |
| 16 |  |  | gi|3868721|gb|AAD13462.1| selenopolypeptide subunit of formate dehydrogenase H; formate dehydrogenase H, selenopolypeptide subunit [Escherichia coli K12] | >ORF 7355 and organism AE014073.seq  MQKFARAVIGTNNVDCCARV**U**HGPSVAGLHQSVGNGAMSNAINEIDNTDLVFVFGYNPADSHPIVANHVI  NAKRNGAKIIVCDPRKIETARIADMHIALKNGSNIALLNAMGHVIIEENLYDKAFVASRTEGFEEYRKIV  EGYTPESVEDITGVSASEIRQAARMYAQAKSAAILWGMGVTQFYQGVETVRSLTSLAMLTSNLGKPHAGV  NPVRGQNNVQGACDMGALPDTYPGYQYVKDPANREKFAKAWGVESLPAHTGYRISELPHRAAHGEVRAAY  IMGEDPLQTDAELSAVRKAFEDLELVIVQDIFITKTASAADVILPSTSWGEHEGVFTAADRGFQRFFKAV  EPKWDLKTDWQIISEIATRMGYPMHYNNTQEIWDELRHLCPDFYGATYEKMGELGFIQWPCRDTSDADQG  TSYLFKEKFDTPNGLAQFFTCDWVAPIDKLTDEYPMVLSTVREVGHYSCRSMTGNCAALAALADEPGYAQ  INTEDAKRLGIEDEALV |
| 17 | AE006469 | *Sinorhizobium meliloti* | gi|2983532|gb|AAC07107.1| formate dehydrogenase alpha subunit [Aquifex aeolicus VF5] | >ORF 3189 and organism AE006469.seq  MEAVPMNVDLSRRSFLKLAGAGAAATSLGAMGFGEAEAAVVAHVRPHKLTTTTETRNTCPYCSVACGVII  YSKGDLRKGEAADIIHIEGDADHPTNRGTLCPKGAALKDFVKSPTRLQYPMHRKPGSDKFERISWEDAFD  RIARLMKDDRDANFIAANAAGVPVNRWTTVGMLAASATTNETAWATFKFAKALGIVGFDNQARV**U**HGPTV  SSLGPTFGRGAMTNSWTDIKNTDLVVVMGGNAAEAHPCGFKWVTEAKATRGAKLIVVDPRYTRTASVSDY  YAPIRQGTDIAFLNGVMKYCIDNDKVQWDYMKAFTNASYLVKDGFGYQDGLFTGYDAEKRDYDKSTWDYV  LGDDGFVVTDPALQHPRCVWNLLKAHLAPYTPEMVERICGTPKDKFLKVAEMISECSSPTKTMTSMYALG  WTQHSSGSQNIRAMAMLQLILGNIGVRGGGMNALRGHSNIQGLTDLGLMSHLLTGYLTMPTEKDVDFTTY  MSTRQFKPLRPGQTSYWQNYRKFMVSFQKAMWGDAARIDNDWAFNYLSKLDVPAYDVLRVFELMYAGKVN  GYICQGFNPLLAFPNRDKNTKALSNLKWLVTMDPLDTETARFWENHGDFNPVDTASIQTEVFQLPTTCFA  EEEGSLTNSGRWLQWHWAGGTPPGEAKHDTYIVAQIFLRMKEMYRNEGGAFPDPILNLSWDYADPNEPTP  EELAKEINGRALTDLMDPANPMKVQVAAGKQILNFSQLRDDGSTMCGCWIYSGNFNEQGNNMARRDNHDP  DDTGAYLGWSFAWPLNRRTLYNRASADLQGKPWDPSRKLLEWDGTKWAGYDVPDIAPTAKPDEIGPFIMN  QEGTARLFSRGLMRDGPFPAHMEPFESPVANVFNPKMRGNPVSRVFQTDVAQMGLSDEFPYAATSYRLTE  HFHYWTKHNRVNSALQPEFFVEISEELAEEKNIENGGWVRVWSKRGSVKAKAVVTKRIRPLMCDGKPVHV  VGIPLHWGFTGSAKKGLGPNSLAPFVGDANIETPEYKAFLVNIEPSTAPEEATV |
| 18 | AE008691 | *Thermoanaerobacter tengcongensis* | gi|41816370|gb|AAS11237.1| glycine reductase complex selenoprotein GrdA [Treponema denticola ATCC 35405] | >ORF 2654 and organism AE008691.seq  MLEGKKVIIVGDRDGIPGPAIEECIKTTGAEVVFTATECFV**U**TAAGAMDLEIQGRIKELAEKYGPENVVV  ILGSNEAEAASLAAETVTNGDPTYAGPLAGVPLGLRVYHILEEEIKKEIDPNVYEEQVGMMEMVLDVEAI  SKEVRSIREQYSKY |
| 19 |  |  | gi|51857693|dbj|BAD41851.1| glycine reductase complex selenoprotein B [Symbiobacterium thermophilum IAM 14863] | >ORF 3295 and organism AE008691.seq  LLRVVHYLNQFFGQIGGEDKASIPPMVKEGPVGPGMAFKGSFGDKAEIVATVICGDSYFNENIEKATQEI  LQMIERYKPDVFIAGPAFNAGRYGTACGTICKAVQDKFGIPAVTGMYPENPGVDLYKKHVYIIKTTDSAA  GMKNAVSKMASLALKLGFKEPIGAPEEEGYIPRGIRKNIFVEERASKRAVEMLIKKLKGEPFTTEYPMPD  FDRVPPAKAVNVKEALIALVTSGGIVPKGNPDHIESSSASKYGKYSIEGIDNLTSETHETAHGGYDPVYA  NEDPDRVLPVDALRELEREGKIGKLYNYYYATVGNGTSVANAKRFAQNIAKELLEAKVQAVILTST**U**GTC  TRCGATMVKELERAGIPTVHVCTIVPISKTVGANRIVPAVAIPHPLGNPSLGPKEEYALRKKLVEKALKA  LETDIEGQTVFE |
| 20 |  |  | gi|46914191|emb|CAG20971.1| Putative selenophosphate synthase [Photobacterium profundum] | >ORF 3296 and organism AE008691.seq  VIEKIKLTQFTKSAG**U**AAKIGPEALAQVLCQLEITWNENLLVGLNTNDDAAVYRLNEDIAIVHTVDYFTP  VVDDPYDFGQIAAANALSDVYAMGAVPLFALNVVCFPAAYIDVLKEVLRGGNDKVKEAGALIAGGHTIED  EEPKYGLSVTGIVHPEKVIKNSTAKPGDVLILTKPLGIGVINTAIKGEMCPSETYLLAVEVMKYLNKEAS  EIMKEVGVNACTDITGFGLLGHAYEMAFSSGVTIEFDKDSIPLIEGARELAQMGLIPGGCYRNKKYLKGK  VCIKVEEDEVIDLMFDPQTSGGLLISVSEEKAEELYRRLNKKLKFGAFIVGRVKEKQEYDIYVR |
| 21 | AE014075 | *�Escherichia coli CFT073* | gi|2983532|gb|AAC07107.1| formate dehydrogenase alpha subunit [Aquifex aeolicus VF5] | >ORF 10682 and organism AE014075.seq  MQVSRRQFFKICAGGMAGTTAAALGFAPSVALAETRQYKLLRTRETRNTCTYCSVGCGLLMYSLGDGAKN  AKASIFHIEGDPDHPVNRGALCPKGAGLVDFIHSESRLKFPEYRAPGSDKWQQISWEEAFDRIAKLMKED  RDANYIAQNAEGATVNRWLSTGMLCASASSNETGYLTQKFSRALGMLAVDNQARV**U**HGPTVASLAPTFGR  GAMTNHWVDIKNANLVVVMGGNAAEAHPVGFRWAMEAKIHNGAKLIVIDPRFTRTAAVADYYAPIRSGTD  IAFLSGVLLYLLNNEKFNREYTEAYTNASLIVREDYGFEDGLFTGYDAEKRKYDKSTWTYELDENGFAKR  DTTLQHPRCVWNLLKQHVSRYTPDVVENICGTPKDAFLKVCEYIAETSAHDKTASFLYALGWTQHSIGAQ  NIRTMAMIQLLLGNMGMAGGGVNALRGHSNIQGLTDLGLLSQSLPGYMTLPSEKQTDLQTYLTANTPKPL  LEGQVNYWGNYPKFFVSMMKAFFGDKATAENSWGFDWLPKWDKGYDVLQYFEMMKEGKVNGYICQGFNPV  ASFPNKNKVIGCLSKLKFLVTIDPLNTETSNFWQNHGELNEVDSSKIQTEVFRLPSTCFAEENGSIVNSG  RWLQWHWKGADAPGIALTDGEILSGIFLRLRKMYAEQGGANPDQVLNMTWNYAIPHEPSSEEVAMESNGK  ALADITDPATGAVIVKKGQQLSSFAQLRDDGTTSCGCWIFAGSWTPEGNQMARRDNADPSGLGNTLGWAW  AWPLNRRILYNRASADPQGNPWDPKRQLLKWDGTKWTGWDIPDYSAAPPGSGVGPFIMQQEGMGRLFALD  KMAEGPFPEHYEPFETPLGTNPLHPNVISNPAARIFKDDAEALGKADKFPYVGTTYRLTEHFHYWTKHAL  LNAILQPEQFVEIGESLANKLGIAQGDTVKVSSNRGYIKAKAVVTKRIRTLKANGKDIDTIGIPIHWGYE  GVAKKGFIANTLTPFVGDANTQTPEFKSFLVNVEKV |
| 22 |  |  | gi|56130341|gb|AAV79847.1| formate dehydrogenase H [Salmonella enterica subsp. enterica serovar Paratyphi A str. ATCC 9150] | >ORF 13281 and organism AE014075.seq  MKKVVTVCPYCASGCKINLVVDNGKIVRAEAAQGKTNQGTLCLKGYYGWDFINDTQILTPRLKTPMIRRQ  RGGKLEPVSWDEALNYVAERLSAIKEKYGPDAIQTTGSSRGTGNETNYVMQKFARAVIGTNNVDCCARV**U**  HGPSVAGLHQSVGNGAMSNAINEIDNTDLVFVFGYNPADSHPIVANHVINAKRNGAKIIVCDPRKIETAR  IADMHIALKNGSNIALLNAMGHVIIEENLYDKAFVASRTEGFEEYRKIVEGYTPESVEDITGVSASEIRQ  AARMYAQAESAAILWGMGVTQFYQGVETVRSLTSLAMLTGNLGKPHAGVNPVRGQNNVQGACDMGALPDT  YPGYQYVKDPANREKFAKAWGVESLPAHTGYRISELPHRVAHGEVRAAYIMGEDPLQTDAELSAVRKAFE  DLELVIVQDIFMTKTASAADVILPSTSWGEHEGVFTAADRGFQRFFKAVEPKWDLKTDWQIISEIATRMG  YPMHYNNTQEIWDELRHLCPDFYGATYEKMGELGFIQWPCRDTSDADQGTSYLFKEKFDTPNGLAQFFTC  DWVAPIDKLTDEYPMVLSTVREVGHYSCRSMTGNCAALAALADEPGYAQINTEDAKRLGIEDEALVWVHS  RKGKIITRAQVSDRPNKGAIYMTYQWWIGACNELVTENLSPITKTPEYKYCAVRVEPIADQRAAEQYVID  EYNKLKTRLREAALA |
| 23 |  |  | gi|2983532|gb|AAC07107.1| formate dehydrogenase alpha subunit [Aquifex aeolicus VF5] | >ORF 5945 and organism AE014075.seq  MDVSRRQFFKICAGGMAGTTVAALGFAPKQALAQARNYKLLRAKEIRNTCTYCSVGCGLLMYSLGDGAKN  AREAIYHIEGDPDHPVSRGALCPKGAGLLDYVNSENRLRYPEYRAPGSDKWQRISWEEAFSRIAKLMKAD  RDANFIEKNEQGVTVNRWLSTGMLCASGASNETGMLTQKFARSLGMLAVDNQARV**U**HGPTVASLAPTFGR  GAMTNHWVDIKNANVVMVMGGNAAEAHPVGFRWAMEAKNNNDATLIVVDPRFTRTASVADIYAPIRSGTD  ITFLSGVLRYLIENNKINAEYVKHYTNASLLVRDDFAFEDGLFSGYDAEKRQYDKSSWNYQFDENGYAKR  DDTLTHPRCVWNLLKAHVSRYTPDVVENICGTPKADFLKVCEVLASTSAPDRTTTFLYALGWTQHTVGAQ  NIRTMAMIQLLLGNMGMAGGGVNALRGHSNIQGLTDLGLLSTSLPGYLTLPSEKQVDLQSYLEANTPKAT  LADQVNYWSNYPKFFVSLMKSFYGDAAQKENNWGYDWLPKWDQTYDVIKYFNMMDEGKVTGYFCQGFNPV  ASFPDKNKVVSCLSKLKYMVVIDPLVTETSTFWQNHGESNDVDPASIQTEVFRLPSTCFAEEDGSIANSG  RWLQWHWKGQDAPGEARNDGEILAGIYHHLRELYQAEGGKGVEPLIKMSWNYKQPHEPQSDEVAKENNGY  ALEDLYDANGVLIAKKGQLLSSFAHLRDDGTTASSCWIYTGSWTEQGNQMANRDNSDPSGLGNTLGWAWA  WPLNRRVLYNRASADINGKPWDPKRMLIQWNGSKWTGNDIPDFGNAAPGTPTGPFIMQPEGMGRLFAINK  MAEGPFPEHYEPIETPLGTNPLHPNVVSNPVVRLYEQDALRMGKKEQFPYVGTTYRLTEHFHTWTKHALL  NAIAQPEQFVEISETLAAAKGINNGDRVTVSSKRGFIRAVAVVTRRLKPLNVNGQQVETVGIPIHWGFEG  VARKGYIANTLTPNVGDANSQTPEYKAFLVNIEKA |
| 24 | BA000007 | *Escherichia coli O157H7* | gi|56130341|gb|AAV79847.1| formate dehydrogenase H [Salmonella enterica subsp. enterica serovar Paratyphi A str. ATCC 9150] | >ORF 11303 and organism BA000007.seq  MKKVVTVCPYCASGCKINLVVDNGKIVRAEAAQGKTNQGTLCLKGYYGWDFINDTQILTPRLKTPMIRRQ  RGGKLEPVSWDEALNYVAERLSAIKEKYGPDAIQTTGSSRGTGNETNYVMQKFARAVIGTNNVDCCARV**U**  HGPSVAGLHQSVGNGAMSNAINEIDNTDLVFVFGYNPADSHPIVANHVINAKRNGAKIIVCDPRKIETAR  IADMHIALKNGSNIALLNAMGHVIIEENLYDKAFVASRTEGFEEYRKIVEGYTPESVEDITGVSASEIRQ  AARMYAQAKSAAILWGMGVTQFYQGVETVRSLTSLAMLTGNLGKPHAGVNPVRGQNNVQGACDMGALPDT  YPGYQYVKDPANREKFAKAWGVESLPAHTGYRISELPHRAAHGEVRAAYIMGEDPLQTDAELSAVRKAFE  DLELVIVQDIFMTKTASAADVILPSTSWGEHEGVFTAADRGFQRFFKAVEPKWDLKTDWQIISEIATRMG  YPMHYNNTQEIWDELRHLCPDFYGATYEKMGELGFIQWPCRDTSDADQGTSYLFKEKFDTPNGLAQFFTC  DWVAPIDKLTDEYPMVLSTVREVGHYSCRSMTGNCAALAALADEPGYAQINTEDAKRLSIEDEALVWVHS  RKGKIITRAQVSDRPNKGAIYMTYQWWIGACNELVTENLSPITKTPEYKYCAVRVEPIADQRAAEQYVID  EYNKLKTRLREAALA |
| 25 |  |  | gi|2983532|gb|AAC07107.1| formate dehydrogenase alpha subunit [Aquifex aeolicus VF5] | >ORF 3799 and organism BA000007.seq  MDVSRRQFFKICAGGMAGTTVAALGFAPKQALAQARNYKLLRAKEIRNTCTYCSVGCGLLMYSLGDGAKN  AREAIYHIEGDPDHPVSRGALCPKGAGLLDYVNSENRLRYPEYRAPGSDKWQRISWEEAFSRIAKLMKAD  RDANFIEKNEQGVTVNRWLSTGMLCASGASNETGMLTQKFARSLGMLAVDNQARV**U**HGPTVASLAPTFGR  GAMTNHWVDIKNANVVMVMGGNAAEAHPVGFRWAMEAKNNNDATLIVVDPRFTRTASVADIYAPIRSGTD  ITFLSGVLRYLIENNKINAEYVKHYTNASLLVRDDFAFEDGLFSGYDAEKRQYDKSSWNYQFDENGYAKR  DETLTHPRCVWNLLKEHVSRYTPDVVENICGTPKADFLKVCEVLASTSAPDRTTTFLYALGWTQHTVGAQ  NIRTMAMIQLLLGNMGMAGGGVNALRGHSNIQGLTDLGLLSTSLPGYLTLPSEKQVDLQSYLEANTPKAT  LAGQVNYWSNYPKFFVSLMKSFYGDAAQKENNWGYDWLPKWDQTYDVIKYFNMMDEGKVTGYFCQGFNPV  ASFPDKNKVVSCLSKLKYMVVIDPLVTETSTFWQNHGESNDVDPASIQTEVFRLPSTCFAEEDGSIANSG  RWLQWHWKGQDAPGEARNDGEILAGIYHHLRELYQAEGGKGVEPLMKMSWNYKQPHEPQSDEVAKENNGY  ALEDLYDANGVLIAKKGQLLSSFAHLRDDGTTASSCWIYTGSWTEQGNQMANRDNSDPSGLGNTLGWAWA  WPLNRRVLYNRASADINGKPWDPKRMLIQWNGSKWTGNDIPDFGNAAPGTPTGPFIMQPEGMGRLFAINK  MAEGPFPEHYEPIETPLGTNPLHPNVVSNPVVRLYEQDALRMGKKEQFPYVGTTYRLTEHFHTWTKHALL  NAIAQPEQFVEISETLAAAKGINNGDRVTVSSKRGFIRAVAVVTRRLKPLNVNGQQVETVGIPIHWGFEG  VARKGYIANTLTPNVGDANSQTPEYKAFLVNIEKA |
| 26 |  |  | gi|2983532|gb|AAC07107.1| formate dehydrogenase alpha subunit [Aquifex aeolicus VF5] | >ORF 8649 and organism BA000007.seq  MQVSRRQFFKICAGGMAGTTAAALGFAPSVALAETRQYKLLRTRETRNTCTYCSVGCGLLMYSLGDGAKN  AKASIFHIEGDPDHPVNRGALCPKGAGLVDFIHSESRLKFPEYRAPGSDKWQQISWEEAFDRIAKLMKED  RDANYIAQNAEGVTVNRWLSTGMLCASASSNETGYLTQKFSRALGMLAVDNQARV**U**HGPTVASLAPTFGR  GAMTNHWVDIKNANLVVVMGGNAAEAHPVGFRWAMEAKIHNGAKLIVIDPRFTRTAAVADYYAPIRSGTD  IAFLSGVLLYLLNNEKFNREYTEAYTNASLIVREDYGFEDGLFTGYDAEKRKYDKSSWTYELDENGFAKR  DTTLQHPRCVWNLLKQHVSRYTPDVVENICGTPKDAFLKVCEYIAETSAHDKTASFLYALGWTQHSVGAQ  NIRTMAMIQLLLGNMGMAGGGVNALRGHSNIQGLTDLGLLSQSLPGYMTLPSEKQTDLQTYLTANTPKPL  LEGQENYWGNYPKFFVSMMKAFFGDKATAENSWGFDWLPKWDKGYDVLQYFEMMKEGKVNGYICQGFNPV  ASFPNKNKVISCLSKLKFLVTIDPLNTETSNFWQNHGELNEVDSSKIQTEVFRLPSTCFAEENGSIVNSG  RWLQWHWKGADAPGIALTDGEILSGIFLRLRKMYAEQGGANPDQVLNMTWNYAIPHEPKSEEVAMESNGK  ALADITDPATGAVIVKKGQQLSSFAQLRDDGTTSCGCWIFAGSWTPEGNQMARRDNADPSGLGNTLGWAW  AWPLNRRILYNRASADPQGNPWDPKRQLLKWDGTKWTGWDIPDYSAAPPGSGVGPFIMQQEGMGRLFALD  KMAEGPFPEHYEPFETPLGTNPLHPNVISNPAARIFKDDAEALGKADKFPYVGTTYRLTEHFHYWTKHAL  LNAILQPEQFVEIGESLANKLGIAQGDTVKVSSNRGYIKAKAVVTKRIRTLKANGKDIDTIGIPIHWGYE  GVAKKGFIANTLTPFVGDANTQTPEFKSFLVNVEKV |
| 27 | U00096 | *Escherichia coli K12* | gi|5105267|dbj|BAA80580.1| 114aa long hypothetical protein [Aeropyrum pernix K1]  could be | >ORF 2131 and organism U00096.seq  MAVEHHAFARVDGERGAIEDGQAHAVLLVQDEGFTDVLYVNHFSPRGSGVGCDADASYPTYGERICRPDK  AFTPHPAPAQFCKTDDTSSCVYG**U**CGSSSTRSVNPLSTTCPCFITSKRSASRRATPKSWVTITTARFSST  TRPRSRSSRRACTDTSSPPVGSSINTSFGCVTRLRAICKRCCIPPEKVVGRSSIRLAGISTSSSHFCAVA  RISP |
| 28 |  |  | gi|2983532|gb|AAC07107.1| formate dehydrogenase alpha subunit [Aquifex aeolicus VF5] | >ORF 2944 and organism U00096.seq  MDVSRRQFFKICAGGMAGTTVAALGFAPKQALAQARNYKLLRAKEIRNTCTYCSVGCGLLMYSLGDGAKN  AREAIYHIEGDPDHPVSRGALCPKGAGLLDYVNSENRLRYPEYRAPGSDKWQRISWEEAFSRIAKLMKAD  RDANFIEKNEQGVTVNRWLSTGMLCASGASNETGMLTQKFARSLGMLAVDNQARV**U**HGPTVASLAPTFGR  GAMTNHWVDIKNANVVMVMGGNAAEAHPVGFRWAMEAKNNNDATLIVVDPRFTRTASVADIYAPIRSGTD  ITFLSGVLRYLIENNKINAEYVKHYTNASLLVRDDFAFEDGLFSGYDAEKRQYDKSSWNYQLDENGYAKR  DETLTHPRCVWNLLKEHVSRYTPDVVENICGTPKADFLKVCEVLASTSAPDRTTTFLYALGWTQHTVGAQ  NIRTMAMIQLLLGNMGMAGGGVNALRGHSNIQGLTDLGLLSTSLPGYLTLPSEKQVDLQSYLEANTPKAT  LADQVNYWSNYPKFFVSLMKSFYGDAAQKENNWGYDWLPKWDQTYDVIKYFNMMDEGKVTGYFCQGFNPV  ASFPDKNKVVSCLSKLKYMVVIDPLVTETSTFWQNHGESNDVDPASIQTEVFRLPSTCFAEEDGSIANSG  RWLQWHWKGQDAPGEARNDGEILAGIYHHLRELYQSEGGKGVEPLMKMSWNYKQPHEPQSDEVAKENNGY  ALEDLYDANGVLIAKKGQLLSSFAHLRDDGTTASSCWIYTGSWTEQGNQMANRDNSDPSGLGNTLGWAWA  WPLNRRVLYNRASADINGKPWDPKRMLIQWNGSKWTGNDIPDFGNAAPGTPTGPFIMQPEGMGRLFAINK  MAEGPFPEHYEPIETPLGTNPLHPNVVSNPVVRLYEQDALRMGKKEQFPYVGTTYRLTEHFHTWTKHALL  NAIAQPEQFVEISETLAAAKGINNGDRVTVSSKRGFIRAVAVVTRRLKPLNVNGQQVETVGIPIHWGFEG  VARKGYIANTLTPNVGDANSQTPEYKAFLVNIEKA |
| 29 |  |  | gi|56130341|gb|AAV79847.1| formate dehydrogenase H [Salmonella enterica subsp. enterica serovar Paratyphi A str. ATCC 9150] | >ORF 6947 and organism U00096.seq  MKKVVTVCPYCASGCKINLVVDNGKIVRAEAAQGKTNQGTLCLKGYYGWDFINDTQILTPRLKTPMIRRQ  RGGKLEPVSWDEALNYVAERLSAIKEKYGPDAIQTTGSSRGTGNETNYVMQKFARAVIGTNNVDCCARV**U**  HGPSVAGLHQSVGNGAMSNAINEIDNTDLVFVFGYNPADSHPIVANHVINAKRNGAKIIVCDPRKIETAR  IADMHIALKNGSNIALLNAMGHVIIEENLYDKAFVASRTEGFEEYRKIVEGYTPESVEDITGVSASEIRQ  AARMYAQAKSAAILWGMGVTQFYQGVETVRSLTSLAMLTGNLGKPHAGVNPVRGQNNVQGACDMGALPDT  YPGYQYVKDPANREKFAKAWGVESLPAHTGYRISELPHRAAHGEVRAAYIMGEDPLQTDAELSAVRKAFE  DLELVIVQDIFMTKTASAADVILPSTSWGEHEGVFTAADRGFQRFFKAVEPKWDLKTDWQIISEIATRMG  YPMHYNNTQEIWDELRHLCPDFYGATYEKMGELGFIQWPCRDTSDADQGTSYLFKEKFDTPNGLAQFFTC  DWVAPIDKLTDEYPMVLSTVREVGHYSCRSMTGNCAALAALADEPGYAQINTEDAKRLGIEDEALVWVHS  RKGKIITRAQVSDRPNKGAIYMTYQWWIGACNELVTENLSPITKTPEYKYCAVRVEPIADQRAAEQYVID  EYNKLKTRLREAALA |
| 30 |  |  | gi|2983532|gb|AAC07107.1| formate dehydrogenase alpha subunit [Aquifex aeolicus VF5] | >ORF 7039 and organism U00096.seq  MQVSRRQFFKICAGGMAGTTAAALGFAPSVALAETRQYKLLRTRETRNTCTYCSVGCGLLMYSLGDGAKN  AKASIFHIEGDPDHPVNRGALCPKGAGLVDFIHSESRLKFPEYRAPGSDKWQQISWEEAFDRIAKLMKED  RDANYIAQNAEGVTVNRWLSTGMLCASASSNETGYLTQKFSRALGMLAVDNQARV**U**HGPTVASLAPTFGR  GAMTNHWVDIKNANLVVVMGGNAAEAHPVGFRWAMEAKIHNGAKLIVIDPRFTRTAAVADYYAPIRSGTD  IAFLSGVLLYLLNNEKFNREYTEAYTNASLIVREDYGFEDGLFTGYDAEKRKYDKSSWTYELDENGFAKR  DTTLQHPRCVWNLLKQHVSRYTPDVVENICGTPKDAFLKVCEYIAETSAHDKTASFLYALGWTQHSVGAQ  NIRTMAMIQLLLGNMGMAGGGVNALRGHSNIQGLTDLGLLSQSLPGYMTLPSEKQTDLQTYLTANTPKPL  LEGQVNYWGNYPKFFVSMMKAFFGDKATAENSWGFDWLPKWDKGYDVLQYFEMMKEGKVNGYICQGFNPV  ASFPNKNKVIGCLSKLKFLVTIDPLNTETSNFWQNHGELNEVDSSKIQTEVFRLPSTCFAEENGSIVNSG  RWLQWHWKGADAPGIALTDGEILSGIFLRLRKMYAEQGGANPDQVLNMTWNYAIPHEPSSEEVAMESNGK  ALADITDPATGAVIVKKGQQLSSFAQLRDDGTTSCGCWIFAGSWTPEGNQMARRDNADPSGLGNTLGWAW  AWPLNRRILYNRASADPQGNPWDPKRQLLKWDGTKWTGWDIPDYSAAPPGSGVGPFIMQQEGMGRLFALD  KMAEGPFPEHYEPFETPLGTNPLHPNVISNPAARIFKDDAEALGKADKFPYVGTTYRLTEHFHYWTKHAL  LNAILQPEQFVEIGESLANKLGIAQGDTVKVSSNRGYIKAKAVVTKRIRTLKANGKDIDTIGIPIHWGYE  GVAKKGFIANTLTPFVGDANTQTPEFKSFLVNVEKV |
| 31 | AE014299 | *Shewanella oneidensis* | gi|2983532|gb|AAC07107.1| formate dehydrogenase alpha subunit [Aquifex aeolicus VF5] | >ORF 31 and organism AE014299.seq  MNRRQFFKLCAAGAATSAISALGLMSEKAYAAVREFKLLGAKETRNNCPYCSVGCGLLMYSQGSGGKNSE  HAIFHIEGDADHPVNRGALCSKGAGLVDYVNSPHRLQYPEYRAPGSNKWERISWQDAFKRIARLMKDDRD  ANLIEKNADGVTVNRWLTTGMMTSSGMANESGLATQKFARALGLVAIDTIARN**U**HSPTVASLAPTFGRGA  MTNHWIDIKNSNVVIIMGGNAAEAHPVGFGWVTEAMQHNNAKLIVVDPRFNRSASLADHYAPIRSGTDIA  FLLGVIRYLISTNQVNFDYVKAYTNASYLVRDDFDFHDGLFSGFDEAKGEYNKESWFYQLDEDGYAIVDE  TLEHPRCVWNLLKQHVERYDFATVSNITGTPTEDYQVVCDAIASTHTKDRVATFMYALGWTHHSKGAQNI  RSMAMIQLLLGNIGQLGGGVNALRGHANVQGSTDMGLLAQSLPGYLKLPNDKEPTLAAHLAANTPKPLRP  GQTNYWQNYPKFYVSLLKAFWGENATPENEFGYQWLPKWDQMYDFGKHLDMMYRGKVNGCIVQGVNAINS  MPNRNKNIKALSNLKFLVVLDNLSSETATFWQNEPGFNEVDTASIQTEVFRLPATVFAEEEGSIVNSGRW  MQWHYKWANPPGEAMSDSEIVSGLLLELRKLYREEGGKLPEPIQAINWNYTDPHNPSSIELTKELNGYDV  ATKRQISSFAELKADGSTASACWVYAGSWTEAGNQMARRDNHDPSGKGITPGWAFAWPLNRRVLYNRASC  DVNGKPWDEHRKIVEWKDGKWEGIDVPDFNAKLNPQESAHPFIMQADGVGRFFALKLLKEGPFPEHYEPV  ESPIGTNPLHPNVVHSPVLRWFEGVKDTIGTKEEYPYACTTYSLTEHFNFWTTHCRLAAIAMPETFVEMN  EQLAAEKGIKNGDWVKVSSKRGHILTKALVTKRMRPLQVNGQTVHTLGIPRHGSHNALTRKSYSCNVLTT  EMGDANTGVPEYKAFLVNVEKAEV |
| 32 | AE015451 | *Pseudomonas putida KT2440* | gi|2983532|gb|AAC07107.1| formate dehydrogenase alpha subunit [Aquifex aeolicus VF5] | >ORF 14534 and organism AE015451.seq  LAATVTTKRRRQCSARPPPDQECTMDLNRRQFFKVAAVGLGGSSLAALGMAPTPAFAEQVRHFKLAHTKE  TRNTCPYCSVGCGLILYSQGDAGKNVKQNIIHIEGDADHPVNRGTLCPKGAGLLDFIHSPSRLQYPEVRK  PGSKEWVRVSWDEALDRVADLMKQDRDANFIEKNAQGQTVNRWLTTGFLAASAASSEAGYLTHKVIRATG  MLGFDNQARVUHGPTVASLAPTYGRGAMTNHWSDIANANLVLVMGGNAAEAHPCGFKWVTEAKAHNKARL  IVVDPRFTRTASVADYYAPIRTGTDIAFMGGLINYLLSNDKIQHEYVRNYTDVSFIVKENYGFEDGLFSG  YDEAKRVYADKSGWGYELGEDGYAKVDPTLQHPRCVFQLMKQHYSRYTPELASMTCGMPQDAMMKVWEEI  ASCSVPGKTMTILYALGWTQHSIGAQIIRSAAMVQLLLGNVGMPGGGVNALRGHSNIQGLTDLGLLSNSL  PGYLTLAGDAEQDYAAYIDKRASKPLRPGQLSYWQNYGKFHVSLMKAWYGANATAENNWGYDWLPKLDVP  AYDVLRMFEMMGQGKVNGYMCQGFNPIAALPDKNRVTAALGKLKWLVIMDPLATETSEFWRNAGPFNDVD  TASIQTEVIRLPTTCFAEEDGSLVNSSRWLQWHWKGADGPGETRTDVQIMSELFLRLRQRYQAEGGAYPD  AIMNISWPYKIPEEPSPEELAKEMNGWAVADVTDPTGAVIKAGQQLAGFGQLKDDGSTASGCWIFAGSWT  EQGNQMARRDNSDPYGMHQVQNWAWAWPANRRILYNRASSDPQGKPWDPEKKRLVWWNGKAWTGTDVPDF  KVDSPPEAGMNPFIMNPEGVARFFAIDKMAEGPFPEHYEPFETPIGINPLHPQNKKATSNPAGRIFDSVW  DTLGTHGEFPYAATTYRLTEHFHFWSKHCRLNAIAQPEQFVEIGEVLANEKGIKAGDRVRVSSKRGHIDA  VAVVTKRIRPLQVNNQTVHQIGIPLHWGFTGATRHGYLTNTLVPFLGDGNTQTPESKSFLVKVEKL |
| 33 | AE004091 | *Pseudomonas aeruginosa* | gi|2983532|gb|AAC07107.1| formate dehydrogenase alpha subunit [Aquifex aeolicus VF5] | >ORF 26585 and organism AE004091.seq  MDMNRRQFFKVCGIGLGGSSLAALGMAPTEAFADQVRHFKLAHTVETRNTCTYCSVGCGLIMYSQGDGAK  NVAQNIIHIEGDADHPVNRGTLCPKGAGLLDYIHSPNRLKYPEVREAGSSEWKRIEWDEALERIAKLMKE  DRDANFVEKNEQGQTVNRWLTTGFLAASASSNEAGYITHKVMRSLGILGFDNQARVUHGPTVASLAPTFG  RGAMTNHWTDIKNADLVLIMGGNAAEAHPCGFKWVTEAKAHNKARLLVVDPRFTRSASVADYYAPIRTGT  DIAFLGGLINYLLENDKIQHEYVRNYTDVSFIVKEGFSFEDGLFNGYDAEKRTYPDKSSWGYEIGEDGYA  KVDPTLTHPRCVFNLLKQHYSRYTPDVVSNICGTPKDMMLKVWAEIAETSKPGKVMTIMYALGWTQHSVG  AQMIRTGAMVQLLLGNIGMPGGGMNALRGHSNIQGLTDLGLLSNSLPGYLTLAMDAEQDYDAYIAKRTAK  PLRPGQLSYWQNYGKFHVSLMKAWFGKSATKENNWCYDWLPKLDMPGAGYDVLRYFDMMYQGKVNGYFCQ  GFNPIASFPNKAKVGAALARLKWMVVMDPLVTETSEFWRNVGEYNDVDTASIKTTVFRLPTSCFAEEDGS  IVNSGRWLQWHWKGAEPPGQARPDIAIMAGLFHRLREMYRKDGGAFPDPILGLDWSYLKPDEPGPDELAR  EFNGKALSDLVDPANGMILAKAGEQLPGFALLRDDGSTASGCWIFAGSWTQQGNQMGRRDNSDPYGMGQT  LGWAWAWPANRRILYNRASADVSGKPWDPEKKRLVWWNGKSWGGTDVPDYKADVPPEAGMNPFIMNPEGV  ARLFAVDKMAEGPFPEHYEPFETPIGVNPLHRDNRKAISNPAARVFKNDMELFGTADEFPYAATTYRLTE  HFHYWTKHCRLNAITQPEQFVEIGEALAKELGINAGDKVKVSSNRGYIKAVAVVTKRIRPLQVDGKTVHH  VGIPIHWGFAGMARNGFLANTLTPFVGDGNTQTPEFKSFLVNVEKA |
| 34 | AE016958 | *Mycobacterium avium paratuberculosis* | gi|13880045|gb|AAK44759.1| hypothetical protein MT0536 [Mycobacterium tuberculosis CDC1551] | >ORF 1363 and organism AE016958.seq  VGTVGFLLGRNPRPTHLLTHSAAAVIHTSRTAKKHASVDDAMVEHMFEYVVASRSTPEAVALLDRAREAA  RAEARAAAARLVAIAELLVLRCRETGERADWAADAWEAVAAQVGAALGCSVAMGHSYLRYAMAMRERLPQ  VGAVFAAGGIDYRAFQTLVFRTDLITDAQVLARVDATLAVLVSRRPSLTRGGLAAAVDRVVAAVDADAVR  RARDAVTGRYLDVRANESGMAWVEGNVLGPDGHALDRRLDELASGVCAGDPRSRAQRRADALGALAAGAG  RLACGCGSPDCPSAAAPAPRSTVVIHVVAEQATVAGRGATPAVVAGLDGLIPAQVIAELAASARLVPVAV  PEGGPEPGYTPSARLADFIRCRDLTCRAPGCDRPAVDCDVDHTIPYAQGGPTHPSNLKCLCRGHHLLKTF  WGWRDRQLPDGTVIWTLPDGDTYVTTPGSALLFPTL**U**TPTGAVSPAKPDTEHRCGERTAMMPLRRHTRAH  NRAHRITTERNHNRRTRLTTHPATPGPAPPPDPDDEPPPF |
| 35 |  |  | gi|2983532|gb|AAC07107.1| formate dehydrogenase alpha subunit [Aquifex aeolicus VF5] | >ORF 6487 and organism AE016958.seq  MAPKKALSKVFLEWPVLRQVRSTDKLGRGSAVTSKHTRALAPRTATADRVVQSVCPYCAVGCGQRVYVKD  ERVVAIEGDPDSPISRGRLCPKGSASEQLVNSPGRQLQVLYRAPRATEWQPLQLDTAIDMIADRFVESRR  NSWQDIDKKGNLLRRTMGIAALGGATLDNEENYLIKKLFTAAGAIQIENQARIUHSATVPGLGASFGRGG  ATQSLQDMANADCIVIQGSNMAECHPVGFQWVEEARARGARVIHVDPRFTRTSAVSDRHIPIRAGSDVVL  LGALINHVLTNDLWFSEYVVAYTNAATIINENFRDTEDLGGLFSGFDPETGQYDTSSWAYEEEDDGRSGG  EIESPGGGHTHGATATHSAAGDEHGSGGPPLAHARVRRDETLQHPRTVFQILKRHYARYTPEMVKDVCGI  SREDFDYLARSIVENSGRERTTCFAYAVGWTQHTLGAQFIRTATILQLLMGNVGRPGSGIMALRGHATIQ  GSTDIPTLFNLLPGYLPMPKAGVHDTLADYLAAVGSKKQKGFWANADAYTVSLLKAWWGEAATADNDWAY  DYLPRLTGPHGTYQTVMGMLADEVEGYFLLGQNPAVGSAHGRMQRLGMSHLKWLVVRDFNLIESATWWKD  GPEIASGELKTEEIETEVFFLPAATHVEKAGSFTQTQRLVQWRHQAVEPPGQCQSELQFFYELGKRIRQR  LAGSTDERDRPLLDLTWDYPTDEHGDPDGEAVLAEINGYRVGEPADPLASYTELRADGSTAAGCWIYTGV  YANAVNQAARRVPNGGASPSQSEWGWAWPADRRVLYNRASADPDGKPWSERKRYIWWDPDQRRWVGYDVP  DFVVDRAPGARPDPELGGPDALAGDDPFIMQADGKGWLFAPKGVVDGPLPTHYEPQESPVANALYPQQRN  PARITFARKDNLSAPSAGEPGSDVYPYVFTTYRLTEHHTAGGMSRWLPYLSELQPEMFCEVSPELAAERG  LQPYGWATIISPRAAIEARVLVTKRVAPLVINGHTVHQIGLPYHWGVGGDAVVSGDAANDLLGVTLDPNV  QIQESKAGSCDIRPGRRPRGEELLRLIADYQSRSGVTAETGNVRVDDAVWERGS |
| 36 | AE017042 | *Yersinia pestis biovar Mediaevails* | gi|2983532|gb|AAC07107.1| formate dehydrogenase alpha subunit [Aquifex aeolicus VF5] | >ORF 6579 and organism AE017042.seq  VTMKEIPMQVSRRQFFKICAGGMAGTTVAALGFAPSVALAETRNYKLLRARETRNTCTYCSVGCGLLMYS  LGDGAKNAKESIFHIEGDPDHPVNRGALCPKGAGLVDFIHSESRLKYPEYRAPGSDKWQRITWDDAFTRI  AKLMKEDRDANFIKTNDAGVTVNRWLSTGMLCASASSNETGYLTQKFSRALGMLAVDNQARV**U**HGPTVAS  LAPTFGRGAMTNHWVDIKNADLIIVMGGNAAEAHPVGFRWAMEAKIHNNAKLLVIDPRFTRTASVADFYT  PIRSGTDIAFLSGVLLYLISNNKINREYVEAYTNASLLVREDYAFDDGLFSGYDAENRKYDKTSWNYQLD  EDGFAKRDVTLQHPRCVWNLLKEHVSRYTPEVVSNICGTPKDDFLQVCEYLAETSVSNKTATFLYALGWT  QHSVGAQNIRTMAMIQLLLGNMGMAGGGINALRGHSNIQGLTDLGLLSQSLPGYLNLPSEKQPDIDTYLK  ANTPKTLLPGQVNYWSNYPKFFVSLMKSFYGDNAQKENGWGYDWLPKWDKGYDVLQYFEMMSQGKVNGYL  CQGFNPIASFPDKNKVTAALSKLKFLVTIDPLNTETANFWQNHGEFNDVDPSKIQTEVFRLPSSCFAEEN  GSIVNSSRWLQWHWKGADSPGEALNDGAILAGIFMRMREMYQREGGAVPEQVLNMTWDYLTPENPEPEEV  AMESNGRALADLTDADGKVLVKKGEQLSTFAQLRDDGTTSSGCWIFAGSWTPAGNQMARRDNADPSGLGN  TLGWAWAWPLNRRILYNRASADPQGKPWDPKRQLLEWDGAKWAGIDVADYSAAAPGSDVGPFIMQPEGMG  RLFAIDKMAEGPFPEHYEPFETPLGTNPLHPNVISNPAARVFKDDLAAMGSHEQFPYVGTTYRLTEHFHY  WTKHALLNAIAQPEQFVEIGEKLAAKKGIKQGDTVKVSSNRGFIKAKAVVTKRIRTLNVHGQEVDTIGIP  IHWGYEGVAKKGFLANTLTPYVGDANTQTPEFKAFLVNVEKV |
| 37 | AE009952 | *Yersinia pestis KIM* | gi|2983532|gb|AAC07107.1| formate dehydrogenase alpha subunit [Aquifex aeolicus VF5] | >ORF 6520 and organism AE009952.seq  VTMKEIPMQVSRRQFFKICAGGMAGTTVAALGFAPSVALAETRNYKLLRARETRNTCTYCSVGCGLLMYS  LGDGAKNAKESIFHIEGDPDHPVNRGALCPKGAGLVDFIHSESRLKYPEYRAPGSDKWQRITWDDAFTRI  AKLMKEDRDANFIKTNDAGVTVNRWLSTGMLCASASSNETGYLTQKFSRALGMLAVDNQARV**U**HGPTVAS  LAPTFGRGAMTNHWVDIKNADLIIVMGGNAAEAHPVGFRWAMEAKIHNNAKLLVIDPRFTRTASVADFYT  PIRSGTDIAFLSGVLLYLISNNKINREYVEAYTNASLLVREDYAFDDGLFSGYDAENRKYDKTSWNYQLD  EDGFAKRDVTLQHPRCVWNLLKEHVSRYTPEVVSNICGTPKDDFLQVCEYLAETSVSNKTATFLYALGWT  QHSVGAQNIRTMAMIQLLLGNMGMAGGGINALRGHSNIQGLTDLGLLSQSLPGYLNLPSEKQPDIDTYLK  ANTPKTLLPGQVNYWSNYPKFFVSLMKSFYGDNAQKENGWGYDWLPKWDKGYDVLQYFEMMSQGKVNGYL  CQGFNPIASFPDKNKVTAALSKLKFLVTIDPLNTETANFWQNHGEFNDVDPSKIQTEVFRLPSSCFAEEN  GSIVNSSRWLQWHWKGADSPGEALNDGAILAGIFMRMREMYQREGGAVPEQVLNMTWDYLTPENPEPEEV  AMESNGRALADLTDADGKVLVKKGEQLSTFAQLRDDGTTSSGCWIFAGSWTPAGNQMARRDNADPSGLGN  TLGWAWAWPLNRRILYNRASADPQGKPWDPKRQLLEWDGAKWAGIDVADYSAAAPGSDVGPFIMQPEGMG  RLFAIDKMAEGPFPEHYEPFETPLGTNPLHPNVISNPAARVFKDDLAAMGSHEQFPYVGTTYRLTEHFHY  WTKHALLNAIAQPEQFVEIGEKLAAKKGIKQGDTVKVSSNRGFIKAKAVVTKRIRTLNVHGQEVDTIGIP  IHWGYEGVAKKGFLANTLTPYVGDANTQTPEFKAFLVNVEKV |
| 38 | AL590842 | *Yersinia pestis CO92* | gi|2983532|gb|AAC07107.1| formate dehydrogenase alpha subunit [Aquifex aeolicus VF5] | >ORF 4966 and organism AL590842.seq  VTMKEIPMQVSRRQFFKICAGGMAGTTVAALGFAPSVALAETRNYKLLRARETRNTCTYCSVGCGLLMYS  LGDGAKNAKESIFHIEGDPDHPVNRGALCPKGAGLVDFIHSESRLKYPEYRAPGSDKWQRITWDDAFTRI  AKLMKEDRDANFIKTNDAGVTVNRWLSTGMLCASASSNETGYLTQKFSRALGMLAVDNQARV**U**HGPTVAS  LAPTFGRGAMTNHWVDIKNADLIIVMGGNAAEAHPVGFRWAMEAKIHNNAKLLVIDPRFTRTASVADFYT  PIRSGTDIAFLSGVLLYLISNNKINREYVEAYTNASLLVREDYAFDDGLFSGYDAENRKYDKTSWNYQLD  EDGFAKRDVTLQHPRCVWNLLKEHVSRYTPEVVSNICGTPKDDFLQVCEYLAETSVSNKTATFLYALGWT  QHSVGAQNIRTMAMIQLLLGNMGMAGGGINALRGHSNIQGLTDLGLLSQSLPGYLNLPSEKQPDIDTYLK  ANTPKTLLPGQVNYWSNYPKFFVSLMKSFYGDNAQKENGWGYDWLPKWDKGYDVLQYFEMMSQGKVNGYL  CQGFNPIASFPDKNKVTAALSKLKFLVTIDPLNTETANFWQNHGEFNDVDPSKIQTEVFRLPSSCFAEEN  GSIVNSSRWLQWHWKGADSPGEALNDGAILAGIFMRMREMYQREGGAVPEQVLNMTWDYLTPENPEPEEV  AMESNGRALADLTDADGKVLVKKGEQLSTFAQLRDDGTTSSGCWIFAGSWTPAGNQMARRDNADPSGLGN  TLGWAWAWPLNRRILYNRASADPQGKPWDPKRQLLEWDGAKWAGIDVADYSAAAPGSDVGPFIMQPEGMG  RLFAIDKMAEGPFPEHYEPFETPLGTNPLHPNVISNPAARVFKDDLAAMGSHEQFPYVGTTYRLTEHFHY  WTKHALLNAIAQPEQFVEIGEKLAAKKGIKQGDTVKVSSNRGFIKAKAVVTKRIRTLNVHGQEVDTIGIP  IHWGYEGVAKKGFLANTLTPYVGDANTQTPEFKAFLVNVEKV |
| 39 | AE017180 | *Geobacter sulfurreducens* | >gi|19918170|gb|AAM07420.1| 4-carboxymuconolactone decarboxylase [Methanosarcina acetivorans str. C2A];  >gi|19916871|gb|AAM06252.1| conserved hypothetical protein [Methanosarcina acetivorans str. C2A]  [AhpD - RECODE] | >ORF 12099 and organism AE017180.seq  MPLPRHSRSAMRALRSSAANTAGCSERPRCGTSRDCVKVRSLNSLITARGQIRHGVDAPGQWRTINRPEP  FRINGLPPKKRLQITGWNRIVPMLGHEVHGKGAMAMKIRKKILDFEYEEVLDARTRELIRVGCAVAVGCP  T**U**LKKHFAAAKEAGATDAELKEALAYGIIAPSGRAKNFVLNMAGELELGD |
| 40 |  |  | >gi|21956737|gb|AAM83670.1|AE013608\_5 glutaredoxin 3 [Yersinia pestis KIM]  [Glutaredpoxin-RECODE[ | >ORF 12150 and organism AE017180.seq  MMVRSLTAMLVLAATVALTPALLHSAPDKPGRTAESRNPSVVIFVGEG**U**PYCDEVERFFTEKGIPYTCRD  IRRDRAAFREWRERYGGEIVPMVVLDGGKKVIDGCDIPAIERALADIRSSRP |
| 41 |  |  | gi|37201109|dbj|BAC96933.1| thiol-disulfide isomerase and thioredoxins [Vibrio vulnificus YJ016]  Thioredoxin RECODE | >ORF 16885 and organism AE017180.seq  MAESGQSFLVACPACGTSNRVPASREGVAGRCGSCRGVLPPLYFQPVPLTDRSFDPFVAGYHGPVLVEFW  APWUPHCRDFAPVVREVARELAGTAAVVQVNTQENPQLAARFGIRGIPALVLLRRGQVLATWSGALPREA  VLSRVRDALR |
| 42 |  |  | gi|2983532|gb|AAC07107.1| formate dehydrogenase alpha subunit [Aquifex aeolicus VF5] | >ORF 5159 and organism AE017180.seq  LRNVIPICWPVTELAQCLPVYASAIFKPARRGLTPKGDVMGISRRQFLQGGALAGAALALSGTPGEASAD  SPDLRTKGTKVTTTVCPFCSVGCGLIVHTKDGKVINAEGDPQHPINQGSLCPKGGALFQIANNDKRLQKV  MYRAPGSDKWEEKSWDWALDRIALRMKETRDKSFKKTELNKKDNKEYVVNRTDGMAFFGGAGLDNEECYL  WTKFARAMGVGQLEHQARLUHSSTVAGLAASFGRGAMTNHWIDLKNSDVIFAIGCNPAENHPISFKWIEA  ALDNGAKLISVDPRFTRTSSKADIYAQIRPGTDIAFLGGMINYAIQNRMIHEEYVREYTNATFIVSEQFD  FQDGMFCAFDDQEKVYDLKSWAYSTGADGKPKRDMSMKDPKCVYQLMKNHYKRYDIDTVCAITGTPKEDY  LKVVKAFCATGRPDKSGTILYAMGITQSTHGSQNVRAVALLQMLLGNIGIAGGGVNALRGESNVQGSTDY  GLLFHILPGYLKSPEFDNVDLKAYVEKWTPKTKDPKSANWWGNTPKYTVSLLKAWYGDNATAENDFCYDY  LPKRMGNYSYVKIMEKMAKGELEGLVCMGMNPAVGGPDSVAAREALGKLKWLVTADLWETETSIFWKRPG  VDPKSIQTEVFMLPAASSIEKEGSISNSGRWAQWRYAAVHPLGDSRSDLHIIDEFYKRIKALYLKDGGAF  PEPLTKLAWNYGTGHEPDVHLVAKEINGYFTKDVTIKEKDKVLEFKKGDQVPMFKYLQDDGSTVSGCWIY  CGSYTNEGNQMARRDPSDPTGLGLFPKWTWCWPVNRRIIYNRASVNPAGEPFNPKRPVIAWDALEKKWKG  DVPDGPWPPMKDDKEGKYPFIMLPEGHGRLYALDMKDGPFPEHYEPVESPAKNLLSKVQTNPVVKVPSNV  SSDTSKFPLIGTTYRVTEHWQAGAMTRSLPWLVELVPDMFVEISETLAKQKGISQGDMVKVSTERGSIQA  KALVTSRLKPFNVQGKLIEQVGMPWHFGYAGLATGDSGNVLTPTVGCANTGIPEFKAFLCNIEKGGKAA |
| 43 |  |  | >gi|34105000|gb|AAQ61356.1| conserved hypothetical protein [Chromobacterium violaceum ATCC 12472]  >gi|53758707|gb|AAU92998.1| HesB/YadR/YfhF family protein [Methylococcus capsulatus str. Bath]  [HESB\_RECODE} | >ORF 5718 and organism AE017180.seq  MTITDAAKAVLAPIVGEHPGKILRVVFEGFGUGGPRLGLVLDEPADNDARMVLNGIEVAVTSNFRSLLDD  QILDYITNEQGEGLVFRRESGDVCC |
| 44 |  |  | gi|32448022|emb|CAD77542.1| peroxiredoxin [Pirellula sp.];  gi|15622885|dbj|BAB66875.1| 155aa long hypothetical bacterioferritin comigratory protein [Sulfolobus tokodaii str. 7]      ```   ```  ```   ``` | >ORF 8425 and organism AE017180.seq  VVAIDEQKPVYELQKELDALREDYLAGMSPEHAATLQRTATELVLSGIVGHAATIGDRAQDFTLPNAVGR  QIRLSEVTAQSTAVVTFYRGAWUPYCSLQLRAYQAVLPRLRELGGELLAISPQTPDKSQATLLKNFLQYE  VLSDVGNLVARSFGLVYPLGEEMRRIYLGFGVNLADYNGDESWELPLPGTFVIDGTMTIRYSFVDADYTR  RLEPATILDVLERIREERGRDDNQAS |
| 45 |  |  | gi|46914191|emb|CAG20971.1| Putative selenophosphate synthase [Photobacterium profundum]  SELD5 | >ORF 9084 and organism AE017180.seq  VPKNLPSGPVPSPTKFSAASASGCRASTSTRRSGTGERRETPMTEGIKLTSLVKAAGUAAKLGPAGLEQA  LSDMVREDDPNLLVGPETADDAGVYRIGEGLALVETVDIITPLVDDPYTFGRIAAANALSDVFAMGGRPV  TAMNLVFFPACALPGSVLSAILAGGHDALREAGACLVGGHTVEDDELKYGLAVTGLISPSRVVRNATARA  GDRLVLTKPLGTGIVSTAIKADMAPAALTAEAVRWMTMLNAEAAGLMLECGASACTDVTGFGLVGHACEV  ARGAGVTLRLHLEQVPVLDGVMGLVADGLVPAGCYRNRDHYAPFVGAPRSDDDRLLPLFDPQTSGGLLLS  LSPSSAGRFLAAAGDRGLFALEVGEVLPAGECAVDIV |
| 46 |  |  | gi|29605647|dbj|BAC69712.1  hypothetical protein [Streptomyces avermitilis MA-4680]  SELW (short) | LRYRRQRSTGGRTMNVRILFCPT**U**SQYPIAAGLARLIEQTEENVSVELDKQAPRSEFAVYLDGEIIFSRLERGRMPEPLDIIPAIRARRHGTSG |
| 47 |  |  | gi|34482757|emb|CAE09757.1|   ``` SULFUR TRANSFERASE PRECURSOR [Wolinella succinogenes] ```  ``` �� ``` | LRVVGKVYKSCGLLMFAYPVYFCCILVPCLHFEEPSHFSRRHVTPMKTKGWGALAVLPLALALAGNVAAEEVKGKVKTVSVKAGQISLTVESKGVMLFRVTDGTTF  ANAASIREIHADDLLQVDYRVDGFDNVAKAVAKVVAKLPEGVTAMDTRELEALVAKGTEEGGYLLIDSRPAGKYNEAHIPTAVSIPFAELEKNPALLTASKDRLLV  FYCGGVTUVLSPKSAGLAKKSGYEKVRVYLDGEPEWKKAELPLESSLAFVEKGNIVLIDLRSPEKVAAGHIPGAVGIPAADLAAAQAKFPAYRGAHLVFYSDSAED  LSQALELARDWNYKNATIFPGGIGAWQKAGKHLKTGAAAATVTYVKKLAPGEVGTEDFQAALKDGRTVVIDARAPGEFEKGHFKGAVNIPAEEAAKRLAEIPADRP  VLVHCSTGTRAEMVYDLVKDKGYNLKYLKAGVEFAADGSYTISE |
| 48 | AE017226 | *Treponema denticola ATCC 35405* | gi|51857694|dbj|BAD41852.1| glycine reductase complex selenoprotein A [Symbiobacterium thermophilum IAM 14863] | >ORF 181 and organism AE017226.seq  MVDLKTKKVIIIGDRDGVPGEAIKLCAESAGAEVVYAATECFV**U**TSAGAMDLENQKRVKDLAEKYGPENV  IVLLGGAEAESSGLACETVTVGDPTFAGPLAGVSLGLLCYHVAEPEIKSQIDPAVYEEQVSMMEMVMDVN  AIIAEISEYRNKGCKFL |
| 49 |  |  | gi|51857693|dbj|BAD41851.1| glycine reductase complex selenoprotein B [Symbiobacterium thermophilum IAM 14863] | >ORF 2515 and organism AE017226.seq  MSKVIVHYINQFFAGKGGEDMADYKPEVIDGTAGPGTGIQGALGDAGKIVKTIICGDNFFNEHEEEAVAF  VKKVLTDTKADLLIAGPGFNAGRYGMACGNAAKVAFELGIPAISGLYEENPGYDVFKAFMYTIKTGNSAV  SMREAVPAIGALAKKLLKGEQICCPEKEGLLPRGVRQNYFAEERGAKRAVDMLIKKIKGEAFVTEYPMPV  FDRVPPQPPVKDITKAKIALVTSGGVVPKGNPDHIEASNASHYGEYSIAGMAALSSKDSETAHGGYDPTY  CNANPNRVLPVDVLRDLEKEGKIGKLHDKYYTTVGNGTAVKRAKKFAEEIVQKLVKDGVQAVILTST**U**GT  CTRCGATMVKEIERFLPVVHIATVVPISKTVGANRIVPAVAIPHPLGDPKMNDADEKKLRRSLVEKALKA  LETPISEQTVF |
| 50 |  |  | gi|56380162|dbj|BAD76070.1| glutathione peroxidase [Geobacillus kaustophilus HTA426] | >ORF 2570 and organism AE017226.seq  MGIYNYTVKDSLGNDFSFNDYKDYVILIVNTACE**U**GLTPHFQGLEALYKEYRDKKFLVAAFPCNQFGGQD  PGTNEEIRNFAQSKYGVSFPIMAKIEVNGENTEPIFSFLKKASNGEDIKWNFAKFLVDKTGERVTAYAPT  VAPEDLKKDIEKLLN |
| 51 |  |  | gi|51857693|dbj|BAD41851.1| glycine reductase complex selenoprotein B [Symbiobacterium thermophilum IAM 14863] | >ORF 2932 and organism AE017226.seq  MIYKAIHYINQFYAGIGGESSADSGFVVLRDKKGPAIGLEGLWHGKMIVTKVICCGDNYINLDRNFEEVC  RQLKKIVEEEKPDVLIAGPAFNAGRYGMACAKICDYVRSNLNLPSVTAMWHENPAVKIYVRNNYIISSTE  TAAGMHKTLQDLADLALKLAKKEKIGPARIEGYLPTGHRYNEYHKKTGAERVVDMLLDKLNGRPYQTEVP  LRGFERVPPAPPIHKMNKTTIALFTTGGLVPIGNPDKLKQAFAEDFKVYDISNRDTLPQGVYESIHGGYD  TTAASAEPNRLIPLDALRQCEAEGIIGGIYPYFGTTCGVGTNVAVSESMGKAWARKIKEEGVGAVILTST  **U**GTCTRCGATICKELDRVGIPNVHINAFTSISESVGANRIVFGGGFTAPVGNPMLPLERETAYRRKIVDK  ALEALQTEVLSPTVFTVDHDKEG |
| 52 |  |  | gi|26108424|gb|AAN80626.1|AE016761\_201 Selenide,water dikinase [Escherichia coli CFT073] | >ORF 3039 and organism AE017226.seq  MSCSLINEDFDLLKAAKNPG**U**GAKLSAGALDKLLKNFSVRNDDNLLVGFNTSDDAAVYKINDKTALISTI  DFFPPVSGDPYIFGQVAAANSLSDIYAMGGEPKLALNLFCITKDMPEDMIKEILRGGFDKVYEAGAIVCG  GHTIYDDSPKYGLAVNGFVHPKKILENSTAKEGDVLILTKPIGTGILLTASKADMSPPEELDRCYKIMAF  LNAKARNIMVKYKINACTDITGFGLLGHLYEMGKGSGMSIEVDYKSVPIYKSVIESAEMGMMPAGVYSNR  NFVGDNIVFENVPLAYQDLMFDPQTSGGLLISVDKEDAAALYEELSQALENTPCGKPAIIGLVTKRDEKI  LRVS |
| 53 |  |  | gi|52209545|emb|CAH35498.1| thioredoxin 1 [Burkholderia pseudomallei K96243] | >ORF 698 and organism AE017226.seq  MIMAVLDITNANFDETVKTAKPVLIDFWAPW**U**PGCVQLSPELQAAEAELGDKAVIAQSNVDNARELAVKF  KFMSIPTLIVLKDGKEVDRHTGYMDKKSLVNFVSKHI |
| 54 | AL111168 | *Campylobacter jejuni* | gi|27362035|gb|AAO10941.1|AE016805\_198 Formate dehydrogenase, alpha subunit [Vibrio vulnificus CMCP6] | >ORF 520 and organism AL111168.seq  MSSVGENIKLTRRSFLKMAALSSLATPLLARSETLREASADELKEAYEGSKKVKTVCTACSVGCGIIAEV  QNGVWVRQEIAQDHPVSSGGHCCKGSDMIDMVRSHVRLKYPMKKENGEWKRISYEQALSEIGEKLAAYRK  ENPESVMFLGSAKLNNEQAYYIRKFAAFFGTNNVDHQARI**U**HSATVAGVANTFGYGAMTNHLGDIQRSKC  IIIIGANPAVNHPVGFRHFLKAKEKGAKLIVVDPRFTKSAAKADIYARIRPGTDIAFMYGMLKIIFDEGL  EDTKYLDERVFGIDKIREEAAKWTVEEVENVTGISKELLVQITHEVAKNKPTTLIWAMGLTQHTVGTSNT  RLAPIVQMVLGNIGKFGGGVNILRGHDNVQGASDMACLSENLPGYYPLNEATWRYYAKIWGVDYEWLLGN  FVSKDWMHKTGLSLARWWAAALNGKDGNDAIDNAGTPLKALVVMGNGITSTAQQVKVKEGLEALELLVLA  DPFVNEAGIIAERKDGIYLLPAATQFETSGSVTATNRSGQWRFKVVDPLYESMEDQEILFELAKKLGFYE  DFTKTLRDEKGEIVWPENATREIAKAVRSIGLNGWSPERLKKHTLYWDKFDEVTLEGKDEVAGEYYGLPW  PCWSDKHPGSPVLYNTDIEVAKGGMGFRNNFGLEYEGESLLAKNAPLNSPIDTGYPQITKDNIEKVLGIT  LSAQEKEKMGSTWSYDDSNIIATKCIEKGIVPYGNAKARAVVWTFKDKIPLHREPLHSPRNDLVQKYPSF  EDQKALYRVDTKFVSVQQAKDYSKEFPLNLVTARLVNLNGAGMENRASMYLTRLTPEMFCEINPELAKEQ  DIKAGDMIWVHSPEGTKIHVRVKVNPGVAKDMIFLPFHFTGVMQGVDLTHNFPEGTKPYASGESANTVTN  YGYDIMCQIPETKGGLCRISKDGK |
| 55 |  |  | gi|54018125|dbj|BAD59495.1| hypothetical protein [Nocardia farcinica IFM 10152]  � (selw) | >ORF 1228 and organism AL111168.seq  MMKVKIAYCNL**U**NYRPQAARVAEELQSDFKDVEVEFEIGGRGDFIVEVDGKVIFSKTQLINCESERFPYQ  NEINQLIKNRV |
| 56 | AL513382 | *Salmonella typhi* | gi|3868721|gb|AAD13462.1| selenopolypeptide subunit of formate dehydrogenase H; formate dehydrogenase H, selenopolypeptide subunit [Escherichia coli K12] | >ORF 11989 and organism AL513382.seq  MKKVVTVCPYCASGCKINLVVDNGKIVRAEAAQGKTNQGTLCLKGYYGWDFINDTQILTPRLKTPMIRRQ  RGGKLESVSWDEALNYVAERLSAIKAKYGPDAIQTTGSSRGTGNETNYVMQKFARAVIGTNNVDCCARV**U**  HGPSVAGLHQSVGNGAMSNAINEIDNTDLVFVFGYNPADSHPIVANHVINAKRNGAKIIVCDPRKIETAR  IADMHIALKNGSNIALLNAMGHVIIEENLYDKAFVASRTEGFEEYSKIVEGYTPESVEEITGVSAQEIRQ  AARMYASAKSAAILWGMGVTQFYQGVETVRSLTSLAMLTGNLGKPSAGVNPVRGQNNVQGACDMGALPDT  YPGYQYVKFPENREKFAKAWGVESLPAHTGYRISELPHRAAHGEVRAAYIMGEDPLQTDAELSAVRKAFE  DLELVIVQGIFMTKTASAADVILPSTSWGEHEGVFSAADRGFQRFFKAVEPKWDLKTDWQIISEIATRMG  YPMHYNNTQEIWDELRHLCPDFYGATYEKMGELGYIQWPCRDTSDADQGTSYLFKEKFDTPNGLAQFFTC  DWVAPIDKLTEEYPMVLSTVREVGHYSCRSMTGNCAALAALADEPGYAQINTADAARLGIEDEALVWVHS  RKGKIITRAQVSDRPNKGAIYMTYQWWIGACNELVTENLSPITKTPEYKYCAVRVEPIADQRAAEQYVID  EYNKLKTRLRESAMG |
| 57 |  |  | gi|2983532|gb|AAC07107.1| formate dehydrogenase alpha subunit [Aquifex aeolicus VF5] | >ORF 1813 and organism AL513382.seq  MQVSRRQFFKICAGGMAGTTAAALGFAPGVALAETRQYKLLRTRETRNTCTYCSVGCGLLMYSLGDGAKN  AKASIFHIEGDPDHPVSRGALCPKGAGLVDFIHSESRLKFPQYRAPGSDKWQQISWEEAFDRIAKLMKED  RDANYQAQNAEGVTVNRWLTTGMLCASASSNETGYLTQKFSRALGMLAVDNQARV**U**HGPTVASLAPTFGR  GAMTNHWVDIKNANLVVVMGGNAAEAHPVGFRWAMEAKIHNGAKLIVIDPRFTRTASVADFYAPIRSGTD  IAFLSGVMLYLLTNEKYNREYTEAYTNASLIVREDFGFDDGLFTGYDADKRQYDKTSWHYELDENGFAKR  DTTLQHPRCVWNLLKQHVSRYTPDVVENICGTPKADFLKVCEYIAETSAKDKTASFLYALGWTQHSIGAQ  NIRTMAMIQLLLGNMGMAGGGVNALRGHSNIQGLTDLGLLSQSLPGYLTLPSEKQTDLQTYLAANTPKPL  LKDQVNYWGNYPKFFVSMMKAFFGDKATAENSWGFDWLPKWDKGYDVLQYFEMMKQGKVNGYICQGFNPV  ASFPNKNKVVASLSKLKYLVTIDPLNTETSTFWQNHGESNDVDPSKIQTEVFRLPSTCFAEENGSIVNSG  RWLQWHWKGADAPGIAMTDGEILAGIFLRLRKMYSEQGGANPEQVLNMTWNYTKPYEPASEEVAMESNGK  ALADLIDPATGAVVVKKGQQLSSFAQLRDDGTTSSGCWIFAGSWTPEGNMMARRDNADPSGLGNTLGWAW  AWPLNRRILYNRASADPQGNPWDPKRQLLKWEGGKWAGWDIPDYSAAAPGSDVGPFIMQPEGMGRLFAID  KMAEGPFPEHYEPFETPLGTNPLHPNVISNPAARIFKDDADALGKADKFPYVGTTYRLTEHFHYWTKHAL  LNAIAQPEQFVEIGEKLANKLGIAHGDTVKVSSNRGYIKAKAVVTKRIRTLKADGKDIDTIGIPIHWGYE  GVAKKGFIANTLTPFVGDANTQTPEFKSFLVNVEKV |
| 58 | AE006468 | *Salmonella typhimurium LT2* | gi|2983532|gb|AAC07107.1| formate dehydrogenase alpha subunit [Aquifex aeolicus VF5] | >ORF 7455 and organism AE006468.seq  MQVSRRQFFKICAGGMAGTTAAALGFAPGVALAETRQYKLLRTRETRNTCTYCSVGCGLLMYSLGDGAKN  AKASIFHIEGDPDHPVSRGALCPKGAGLVDFIHSESRLKFPQYRAPGSDKWQQISWEEAFDRIAKLMKED  RDANYQAQNAEGVTVNRWLTTGMLCASASSNETGYLTQKFSRALGMLAVDNQARV**U**HGPTVASLAPTFGR  GAMTNHWVDIKNANLVVVMGGNAAEAHPVGFRWAMEAKIHNGAKLIVIDPRFTRTASVADFYAPIRSGTD  IAFLSGVMLYLLTNEKYNREYTEAYTNASLIVREDFGFDDGLFTGYDADKRQYDKTSWHYELDENGFAKH  DTTLQHPRCVWNLLKQHVSRYTPDMVENICGTPKADFLKVCEYIAETSAKDKTASFLYALGWTQHSIGAQ  NIRTMAMIQLLLGNMGMAGGGVNALRGHSNIQGLTDLGLLSQSLPGYLTLPSEKQTDLQTYLAANTPKPL  LKDQVNYWGNYPKFFVSMMKAFFGDKATAENSWGFDWLPKWDKGYDVLQYFEMMKQGKVNGYICQGFNPV  ASFPNKNKVVASLSKLKYLVTIDPLNTETSTFWQNHGESNDVDPAKIQTEVFRLPSTCFAEENGSIVNSG  RWLQWHWKGADAPGIAMTDGEILAGIFLRLRKMYSEQGGANPEQVLNMTWNYTKPYEPASEEVAMESNGK  ALADLIDPATGAVVVKKGQQLSSFAQLRDDGTTSSGCWIFAGSWTPEGNMMARRDNADPSGLGNTLGWAW  AWPLNRRILYNRASADPQGNPWDPKRQLLKWEGGKWAGWDIPDYSAAAPGSDVGPFIMQPEGMGRLFAID  KMAEGPFPEHYEPFETPLGTNPLHPNVISNPAARIFKDDADALGKADKFPYVGTTYRLTEHFHYWTKHAL  LNAIAQPEQFVEIGEKLANKLGIAHGDTVKVSSNRGYIKAKAVVTKRIRTLKADGKDIDTIGIPIHWGYE  GVAKKGFIANTLTPFVGDANTQTPEFKSFLVNVEKV |
| 59 |  |  | gi|2983532|gb|AAC07107.1| formate dehydrogenase alpha subunit [Aquifex aeolicus VF5] | >ORF 8686 and organism AE006468.seq  MDVSRRQFFKICAGGMAGTTVAALGFTPKMALAQARNYKLLRAKEIRNSCTYCSVGCGLLMYSLGDGAKN  AKEAIYHIEGDPDHPVSRGALCPKGAGLLDYVHSEDRLRYPEYRAPGSDKWQRISWDDAFTRIAKLMKAD  RDANFIEKNEQGVTVNRWLSTGMLCASAASNETGMLTQKFARSLGMLAVDNQARV**U**HGPTVASLAPTFGR  GAMTNHWVDIKNANVVMVMGGNAAEAHPVGFRWAMEAKNNNDATLIVVDPRFTRTASVADIYAPIRSGTD  ITFLSGVLLYLIENNKINAEYVKHYTNASLLVRDDFAFDDGLFSGYDAQKRQYDKSSWNYQFDENGYAKR  DETLTHPRCVWNLLKQHVSRYTPDVVENICGTPKADFLKVCEVLASTSVPDRTTTFLYALGWTQHTVGAQ  NIRTMAMIQLLLGNMGMAGGGVNALRGHSNIQGLTDLGLLSTSLPGYLTLPSEKQADLQTYLAANTPKAT  LADQVNYWGNYPKFFVSLMKSFYGDAAQQENDWGFAWLPKWDQSYDVIKYFNMMDSGKVTGYFCQGFNPV  ASFPDKNKVVQSLSKLKYLVVIDPLVTETSTFWQNHGESNDVDPTTIQTEVFRLPSTCFAEEDGSIANSG  RWLQWHWKGQDAPGEARNDGEILAGIYHRLREMYRAEGGKGAEPLLKMSWNYKQPDEPHSEEVAKENNGY  ALEDLYDANGTLLARKGQLLSSFALLRDDGTTSSSCWIYTGSWTEQGNQMSRRDNADPSGLGNTLGWAWA  WPLNRRVLYNRASADPQGKPWDPKRMLIQWNGAKWTGNDIPDFNNAAPGSGTNPFIMQPEGLGRLFAIDK  MAEGPFPEHYEPMETPLGTNPLHPNVVSNPAARLYEEDALRMGKKEQFPYVGTTYRLTEHFHTWTKHALL  NAIAQPEQFVEISETLAAAKGIANGDYVKVSSKRGFIRAVAVVTRRLRTLHVNGQQVETVGIPIHWGFEG  VARKGYIANTLTPNVGDANSQTPEYKAFLVNIEKA |
| 60 |  |  | gi|3868721|gb|AAD13462.1| selenopolypeptide subunit of formate dehydrogenase H; formate dehydrogenase H, selenopolypeptide subunit [Escherichia coli K12] | >ORF 9676 and organism AE006468.seq  MKKVVTVCPYCASGCKINLVVDNGKIVRAEAAQGKTNQGTLCLKGYYGWDFINDTQILTPRLKTPMIRRQ  RGGKLESVSWDEALNYVAERLSAIKAKYGPDAIQTTGSSRGTGNETNYVMQKFARAVIGTNNVDCCARV**U**  HGPSVAGLHQSVGNGAMSNAINEIDNTDLVFVFGYNPADSHPIVANHVINAKRNGAKIIVCDPRKIETAR  IADMHIALKNGSNIALLNAMGHVIIEENLYDKAFVASRTEGFEEYSKIVESYTPESVEEITGVSAQEIRQ  AARMYASAKSAAILWGMGVTQFYQGVETVRSLTSLAMLTGNLGKPSAGVNPVRGQNNVQGACDMGALPDT  YPGYQYVKFPENREKFAKAWGVESLPAHTGYRISELPHRAAHGEVRAAYIMGEDPLQTDAELSAVRKAFE  DLELVIVQDIFMTKTASAADVILPSTSWGEHEGVFSAADRGFQRFFKAVEPKWDLKTDWQIISEIATRMG  YPMHYNNTQEIWDELRHLCPDFYGATYEKMGELGYIQWPCRDTSDADQGTSYLFKEKFDTPNGLAQFFTC  DWVAPIDKLTEEYPMVLSTVREVGHYSCRSMTGNCAALAALADEPGYAQINTADAARLGIEDEALVWVHS  RKGKIITRAQVSDRPNKGAIYMTYQWWIGACNELVTENLSPITKTPEYKYCAVRVEPIADQRAAEQYVID  EYNKLKTRLRESAMG |
| 61 | BA000016 | *�Clostridium perfringens* | gi|28202985|gb|AAO35429.1| conserved protein [Clostridium tetani E88]  gi|20906561|gb|AAM31712.1| HesB protein [Methanosarcina mazei Goe1] | >ORF 1399 and organism BA000016.seq  MSVVKMSNEAYTEFKSFLQENGVEKFDIRINLAGVG**U**GGPVFNIVLDEQSDNDEVVKIEDITFFVDKELV  KDFEGFTLLSSDENGGRGLSLKPVKESEGGCSSCSSCH |
| 62 |  |  | gi|46914191|emb|CAG20971.1| Putative selenophosphate synthase [Photobacterium profundum] | >ORF 1849 and organism BA000016.seq  MIEKRLTELSKTSG**U**AAKIGPGALSEILSKLPKMNDKNLIVGIDTSDDAAVYKLNDEMATIQTLDFFTPI  VDDPYTFGQIAAANSLSDVYAMGGKPIVALNIVCFPNCLNMNILGEILRGGADKVLEAGAVIVGGHTVQD  DEPKYGLSVTGIVHPDKVLKNYGSETGDILILTKPIGLGIINTAIKAKIASKEAYEKAVKVMAYLNKYAG  EIITDYNITSCTDITGFSLIGHAYEMAEPSKKTFRIFKDAIPFIKEAKEYASMGLIPAGCYENKRYLEGK  YLLNNVESWMEDILFDPQTSGGLLISCKEKDYIDILTRLEKLEVESAVIGRVEDFNDAYIVVE |
| 63 | BX470251 | *�Photorhabdus luminescens* | gi|2983532|gb|AAC07107.1| formate dehydrogenase alpha subunit [Aquifex aeolicus VF5] | >ORF 1552 and organism BX470251.seq  VSEEIPMQVSRRQFFKICAGGMAGTTVAALGFAPTAALAQTRNYKLLRARETRNTCTYCSVGCGLLMYSL  GDGAKNAKATIFHIEGDPDHPVNRGALCPKGAGLVDFIHSESRLKYPEYRAAGSDKWQRITWDEAFDRIA  KLMKADRDANFIKTNQEGVTVNRWLTTGMLCASAASNETGYLSQKFSRALGMLAVDNQARV**U**HGPTVASL  APTFGRGAMTNHWVDIKNANLIVVMGGNAAEAHPVGFRWAMEAKIHNNAKLIVIDPRFTRTASVADFYTP  IRSGTDIAFLSGVILYLLTNDKINHEYVEAYTNASLIVREDYSFDDGLFSGYDAEKRQYDKTSWNYALDE  NGFAQRDITLKHPRCVWNLLKEHVSRYTPDVVSNICGTPKEDFLKVCEYIAETCVKDKTASFLYALGWTQ  HSVGAQNIRTMAIIQLLLGNMGMAGGGVNALRGHSNIQGLTDLGLLSQSLPGYLTLPSEKQADLQTYLQA  NTPKPMRPGQVNYWGNYPKFFISLMKSFYGDNARKDNDWGFDWLPKWDKGYDVLQFFDMMSKGEVNGYIC  QGFNPVASFPNKNKVVGALSKLKFLITIDPLNTETSTFWQNHGEFNEVDSSKIQTEVFRLPCCCFAEENG  SIVNSGRWLQWHWKGADAPGEAIGDGEILSGIFKRLRDMYRTEGGASPEPMLSMTWDYFNPDNPTSEEVA  QESNGRALVDLIDANGNVIVKQGQQLTSFAQLRDDGTTASGCWIFAGSWTPEGNQMARRDNADPSGLGNT  LGWAWAWPLNRRILYNRASADPQGKPWDPKRQLLTWDGTKWGGVDTADYSVAAPETDVGPFIMQPEGMAR  LFAIDKMAEGPFPEHYEPFETPLGTNPLHPNVVSNPAARVFKSDFEAMGKPDKFPYVGTTYRLTEHFHYW  TKHALLNSIIQPEQFVEIGEKLAEKKGIKHGDTVKVSSNRGYIKAKAVVTKRIRTLNVHGREVDTIGIPI  HWGFEGAAKKGFIANTLTPFVGDANTQTPEFKAFLVNIEKV |
| 64 | BX571656 | *Wolinella succinogenes* | gi|27362035|gb|AAO10941.1|AE016805\_198 Formate dehydrogenase, alpha subunit [Vibrio vulnificus CMCP6] | >ORF 1016 and organism BX571656.seq  MSENIVQTLSPLKVGRRSFLKMAALAGAMGASSAVASEGVVRSATTQELKEAHPGAKKIKTICTACSVGC  GIVAEVKNDVWVRQEVAQDHPISLGGHCSKGAGMIDVLRSPKRVKYPMKKENGKWKRISWDQAMDEISAK  MLQLRTDFGPDAVQFFGSAKVSTEQAYYIRKFAAFWGTNNVDHQARV**U**HSSTVAGVANTFGYGAMTNHLA  DIQKSKAIIIFGANPAVNHPVGFQHFLKAKEINGSKLIVVEPRFTRTAAKADMFAQIRPGTDIPFMYGMI  NLILKNGWEDKKFVAERTFGFEEIAKEAAKYTPEVVEDITGVPAQQLIDITRVYAQTKPGTLIWAMGLTQ  HTIGTSNTRLAPILQLILGNMGKPGGGTNILRGHDNVQGASDMGCLAENLPGYFPNAEPSFKHWANVWQV  DFEWLKARFAPDMMFKNGFSLSRWWQGVLEEETIHNGPAGKLRAMVCMGNGLISVAQTEKVKQALDKLEL  FVMIDIFPHDAIAYTDRKDGVYLLPAASQYETSGTVTATNRSGQWRYQVVNPIYESKADQDILFAFAKKF  GFYNEYVRALGDGKGNFVWPEDATREIAKGVKTIGLSGWLPERLKAHTDNWHMFDELTLEGKGPMKGEYY  GLPWPCWSDKHPGTPNLYDNSLPVMKGGMGFRNNFGLKQELNGVEYDMLASEGSVPPGGTQKGGYPAITA  ANIEALAGITLTEEEKAKVAGKAWHTDLSMILVNKALEAGLCPYGNARARMFVKEWADQIPRHREPLHSP  RTDMVAKYPSFKDKPNHFRVDTKYESIQMQKDWAKEFPLNLITGRLVTHNGQGIESRISPALSEIYPEMF  IEIHPDRALKLGIKDGDMVWVHSPEGTKGYMKAKYSYSIKEDCVFAPFHWAGIHQGKDLSKNYPEGLVPY  SVGESINTVTNYGYDIVTQIPETKGGLCRIEKA |
| 65 | L42023 | *Haemophilus influenzae* | gi|2983532|gb|AAC07107.1| formate dehydrogenase alpha subunit [Aquifex aeolicus VF5] | >ORF 1 and organism L42023.seq  LLLKGVIMQVSRRKFFKICAGGMAGTSAAMLGFAPANVLAAPREYKLLRAFESRNTCTYCAVSCGMLLYS  TGKPYNSLSSHTGTNTRSKLFHIEGDPDHPVSRGALCPKGAGSLDYVNSESRSLYPQYRAPGSDKWERIS  WKDAIKRIARLMKDDRDANFVEKDSNGKTVNRWATTGIMTASAMSNEAALLTQKWIRMLGMVPVCNQANT  **U**HGPTVASLAPSFGRGAMTNNWVDIKNANLIIVQGGNPAEAHPVGFRWAIEAKKNGAKIIVIDPRFNRTA  SVADLHAPIRSGSDITFLMGVIRYLLETNQIQHEYVKHYTNASFLIDEGFKFEDGLFVGYNEEKRNYDKS  KWNYQFDENGHAKRDMTLQHPRCVINILKEHVSRYTPEMVERITGVKQKLFLQICEEIGKTSVPNKTMTH  LYALGFTEHSIGTQNIRSMAIIQLLLGNMGMPGGGINALRGHSNVQGTTDMGLLPMSLPGYMRLPNDKDT  SYDQYINAITPKDIVPNQVNYYRHTSKFFVSMMKTFYGDNATKENGWGFDFLPKADRLYDPITHVKLMNE  GKLHGWILQGFNVLNSLPNKNKTLSGMSKLKYLVVMDPLQTESSEFWRNFGESNNVNPAEIQTEVFRLPT  TCFAEEEGSIVNSGRWTQWHWKGCDQPGEALPDVDILSMLREEMHELYKKEGGQGIESFEAMTWNYAQPH  SPSAVELAKELNGYALEDLYDPNGNLMYKKGQLLNGFAHLRDDGTTTSGNWLYVGQWTEKGNQTANRDNS  DPSGLGCTIGWGFAWPANRRVLYSRASLDINGNPWDKNRQLIKWNGKNWNWFDIADYGTQPPGSDTGPFI  MSAEGVGRLFAVDKIANGPMPEHYEPVESPIDTNPFHPNVVTDPTLRIYKEDREFIGSNKEYPFVATTYR  LTEHFHSWTAQSALNIIAQPQQFVEIGEKLAAEKGIQKGDMVKITSRRGYIKAVAVVTKRLKDLEIDGRV  VHHIGLPIHWNMKALNGKGNRGFSTNTLTPSWGEAITQTPEYKTFLVNIEKVGEA |
| 66 |  |  | gi|26108424|gb|AAN80626.1|AE016761\_201 Selenide,water dikinase [Escherichia coli CFT073] | >ORF 53 and organism L42023.seq  MEEKIRLTQYSHGAG**U**GCKISPKVLGTILHSELEKFYDPNLIVGNETADDAAVYDLGNGTAIISTTDFFM  PIVDDPFDFGRIAATNAISDIFAMGGKPIMGIAILGFPTNVLPAEVAQKIVDGGRFACHQAGIALAGGHS  IDSPEPIFGLAVTGVIDTEKVKRNASAKSGCKLYMTKPLGIGILTTAEKKGKLKPEHQGLATAAMCQMNS  IGSQFSQVDGVTAMTDVTGFGLLGHLIEICEGSNLSAVVFSDKIKTLDGVKDYIAQGCVPGGTGRNFDSY  GHKVGILTEEQKAILCDPQTSGGLLVAVELNSVQTVIDIAKDAGIDLYEVGKLKPKSESDIVVEVK |
| 67 | CR354531 | *Photobacterium profundum* | gi|58428447|gb|AAW77484.1| conserved hypothetical protein [Xanthomonas oryzae pv. oryzae KACC10331] | >ORF 4256 and organism CR354531.seq  MFSSSIIIAKACSFDVPHISGQALKLWHTAGRSGLPKILQRFCSFSUMQRTPAGLPFGMPGIAEEILGAI  QQHHNLSDIPSHTLYFTKIYRWVLQPITTFTKGCASYHSSFSGSKFHSF |
| 68 | CR354532 | *Photobacterium profundum* | gi|41816370|gb|AAS11237.1| glycine reductase complex selenoprotein GrdA [Treponema denticola ATCC 35405] | >ORF 1774 and organism CR354532.seq  VNKEVFATKTAIILGDRDGIPGQAIEACIKTTGAHVAFSTTECFVUTSAGAMDLENQKRIKALADEFGAE  NIIVILGGAEAEASGLACETVTTGDPTFAGPLAGVQLGLSCYHVVEDAIKEAVDPAVYEEQIGMMEMVLD  VDAIKAEMQQYREEPVEA |
| 69 |  |  | gi|51589698|emb|CAH21328.1| selenide, water dikinase [Yersinia pseudotuberculosis IP 32953] | >ORF 1776 and organism CR354532.seq  MRSYRPLLRKLEMTDKAVRLTQMTKKAGUAAKIGPKAMAQVLQTISPLFPEQDYPNLMVGLAVSDDAAVY  KINDDVAVIQTLDFFTPIVDDPYDFGAIAAANALSDVYAMGGQVTLAMNIFCVPVDLPQEVVGQILKGGA  DKVREAGAVLVGGHTVEDDEPKFGLSVMGMIHPSKVQTKAAVESGDILVLTKPLGTGVISTAAKRGKASQ  ESIQTSTDSMKKLNRNAAQIFVKYPIKACTDITGYSLLGHALEMAEKSDVCMHFIADQVPFLLGAEDYAA  QGIFPGGANRNLEAYKDDIEFAPELDESWQQKLCCPETSGGLLATVPKDCLESLLLEFSNTGESCWVVGY  AESGSGIKVS |
| 70 |  |  | gi|41816370|gb|AAS11237.1| glycine reductase complex selenoprotein GrdA [Treponema denticola ATCC 35405] | >ORF 2210 and organism CR354532.seq  MGLYQAIDYNVSLVICNPSELTMLKDKKVIILGDRDGIPGQAIEACIKSAGAHVLFSTTECFV**U**TSAGAM  DLENQKRIKGFAEEFGAENILIVLGGAEAEASGLACETVTNGDPTFAGPLAGVQLGLSCYHVVEPEIKNN  VDADVYDEQIGMMEMVLDVDAIIAEIKGYREQFGKYVLAEAEV |
| 71 |  |  | gi|41818450|gb|AAS12639.1| glycine reductase complex selenoprotein GrdB2 [Treponema denticola ATCC 35405] | >ORF 2211 and organism CR354532.seq  MTLRVVYYLNQFFAQKGGEEMAHIPMEVVEGSVGVGSQINTMLKDKAEVTHTIICGDSYLNENESLCCHS  LKEILAQLKPDLVVAGPAFNAGRYGMACGTVAKVAHEMGIKTISGMYVENPGYELFGQYAYIAETGNSAA  SMRQAIPAMVKLINRFIETDGELGDPADEGYMPRGIRVNYFAEKRGATRAVDLLIAKLAGPEFTTEYPMP  VFDRVDPQPAIGLLSQAKIALVTSGGVVPKGNPDHIESSSASKYGEYSIEGLDTITCATHETAHGGYDPV  ACNDNPNRVLPVDVLRDMEREGIIGSLHNVFYSTVGNGTAVAKSKEYGAEIAMKLQQAGVTAAIFTST**U**G  TCTRCGATMVKEIEKVMPVVHIATVVPISKTVGANRIVPAIAIPYPLGNPAMQPEEELYNRRQIVEKSLV  ALQTEISEQTVF |
| 72 | AE009439 | *Methanopyrus kandleri* | gi|2622673|gb|AAB86026.1| formate dehydrogenase, alpha subunit homolog [Methanothermobacter thermautotrophicus]  gi|2622681|gb|AAB86033.1| tungsten formylmethanofuran dehydrogenase, subunit B� [Methanothermobacter thermautotrophicus] | >ORF 1324 and organism AE009439.seq  VARKVIKDVVCPFCGTLCDDLEVVVEDGEIVEVRHACRIGAAKFLTAQEDHRHTEPMIKENGEWKKIDYE  DAAEETAKMLVEAKLPVLYGWSATLVEAQEKGVELAELVGGIIDNTASVUHGPSVLGLQDVGVPSCTLGE  VKNRADTVIYWGSNPMHAHPRHMSRYTAFTRGFFRPKGREDRTIIVVDPRKTATARLADVYIRVRPNEDY  ELISALRAAVHGIEIEREEVAGVPTEQIYEVADLIKEASFGTLFWAMGLTMSRGRHRNIDNAICLIKDLN  EYTKWTLIMMRGHYNVTGFNEVLAWTTGYPYAVDFSRGYPRYNPGETSTVDLLTRGEVDAMMVIASDPGA  HFPRKAVEHMARIPLVCVDPHWTPTAELADLYVPVTIAGIEWEGTAYRMDSVPIRMRKVVEPPESMLNDV  EFLEMVIEKVEEM |
| 73 |  |  | gi|57160335|dbj|BAD86265.1| probable formate dehydrogenase, alpha subunit [Thermococcus kodakaraensis KOD1] | >ORF 1700 and organism AE009439.seq  VARMRFVPQVCPFCGCGCGILVGTDGEEIKLLEPWRRHPVNEGRQCVKLWELPEAVQKDRLERPVRMTES  GEPRELSWNRALEEVAEVLSTHEPEEVYFVTSAKATNEDNYVAQKLARTLGTNNVDHCARLUHAPTVVAL  SELLGSGAMTNSIPDLVEADCYLVAGSNTAEQHPIVYRRILQGLEENDADLIVLDPRRTQIAELADIHLQ  VRPRTDLIVFLYMAKVIVEEGLHDGTFIEERTTGFESFEEYVREAVSEGDVRRIAGVDPEDVRKAAVRYA  EAERGCILYCMGLTHHDIATRTVRALCALALLTGNVGRPGTGVNPLRGQNNVQGACDVGALATHFPGYRP  INTETANEMSKIWSFEVPDEPGLKLTEAFDADEITVMYVVGENPAVSEPNTRHAVEKLESLEFLVVQDLY  LTETGELADLVLPAAGWAERTGTFTATDRRVQLAEKAVEPPGEARPDWWILEAVARRLGLKGFGHRSPRE  VFEEIRRVVPQYRGITYERLRRRPGGIHWPCPSEDHPGTPILHTEEFATEDGKARFPKPEDVEYREPERD  VDEEYPLILTTGRVYAHYHTRTITRRSRLLSEEVPESFVEIHPKDAERYGVRDGELVVVETPYGEWRCRA  RVTDRVREGTIFTPFHFGENVLTPHDVRDPESGIPEYKYVPARVRPDSRGSASRG |
| 74 |  |  | gi|33566318|emb|CAE37231.1| putative iron-sulfur binding protein [Bordetella parapertussis]  >gi|27362032|gb|AAO10938.1|AE016805\_195 Ferredoxin [Vibrio vulnificus CMCP6]  gi|59480071|gb|AAW85858.1| formate hydrogenlyase subunit 6 [Vibrio fischeri ES114] | >ORF 5492 and organism AE009439.seq  LLQDFLNHILSREGSKRLLDAHTSREIMQRPPRFRDFPDVDLDRCILCGACADACPVEGRDGCPPAMEMS  EEGPVLHKERCIRCGLCVEVCPTGAIEMGTLHEEVEERVQPPKPARIVVDSDLCVGCGKCESACPSDAIT  VEETAEVDEERCVLCEVCLEVCPVAGAIKLVPTDTDELVKRWKEYLEASLRGUPVVVDLHFSFEDRLRNV  TINIVEVERPDECAGCGLCAEVCPTGAIEVDERVRLDEDRCVACSFCVQACPRDVFRFYEVSFTELKPKR  RPVRVPKADIEVRFIGVDLRTCDRCENRPCIEVCPTGVMREIIEEHRIDLDACHGCLECVKVCPYGSVTV  ELEVPQLKRRSNPRLNRELCVECNRCHEVCPTGAADNVPDGDPDPERCLGCYNCVAYCPTEALKRPDHRP  RPKCTDEVFYIQPDMCIGCRICYDVCPVDAIRIEEITRMPVIMPDLCVRCGLCADACPTSAVDRVPTEEA  EREVLRSRISDAFLGILTREMLEAAEEFGSTTRTERDVEEKLSELLERKMSEEMIRRVIEFEVKNVIEEL  MAEVVSGRDSRGP |
| 75 |  |  | gi|44921146|emb|CAF30381.1| heterodisulfide reductase subunit A [Methanococcus maripaludis]  gi|19916866|gb|AAM06247.1| heterodisulfide reductase, subunit A/methylviologen reducing hydrogenase, subunit delta [Methanosarcina acetivorans str. C2A] | >ORF 5638 and organism AE009439.seq  VGDKDDVRIGVFVCHCGVNIKASVDVEEVVEYAKKLPGVVYATDYPFFCADPGQEIIQEAIKEHDLDRVV  VAACTPKIHENTFRNCVKEAGLSPYYMEMVNIREHCSFVHMQEPEKATEKAKDLIRAAVERAKRLEDVPT  KEVEVENSVLIIGGGIAGIQAALDLADQGFKVYLVEKEPTIGGNMARLAKTFPTDDCAMUILAPKMVQVG  NHPNIEMITYAEVKDVDGYIGNFEVTIEKKPRYVDEDACTGCGVCAEVCPIEVPNEFDLGIGTRKAIYVP  FPQAMPLVYTIDMEHCIQCGLCEEACPQDPPAIDFDQEPEEIRLKVGTIIVATGYEEFDASKLEEYGYGK  YDNVITTLELERMINPAGPTEGHVIRPSDGKEPHRIVFIHCVGSRCPGKEEKGEAYCSRICCMFILKNAQ  LIKQHEPDAEVYCCYMDVRAFGKGYEEYYERAQKQFGVRFIRGRPAEIVEDPETKNLIVRVEDTLTGEPM  EIEADLVVLGCGLVAPEETYSKLADILGIDRSPDGFFKELHPKLEPVSTKVRGVQIAGVAQGPKDIPDTV  AQAKGAASEASIPMSQGKVEIELITATVDEDVCGGCGACAQVCPFDAIEMVEKDGKRVAEVQDVACQGCG  QCAAACPSGAMQLRYYRDEQLMPQIEALLAEALEEEEEE |
| 76 |  |  | gi|44921142|emb|CAF30377.1| coenzyme F420-non-reducing hydrogenase subunit delta [Methanococcus maripaludis]  gi|2622243|gb|AAB85627.1| methyl viologen-reducing hydrogenase, delta subunit homolog FlpD [Methanothermobacter thermautotrophicus]  \gi|20904385|gb|AAM29752.1| Heterodisulfate reductase, subunit A [Methanosarcina mazei Goe1] | >ORF 8331 and organism AE009439.seq  VRVPCTGRVGIEHILTALAKGAWTVFVAGUKKGECSYEDGNLKCERRVQAAKKLLEELGIEPERVEIYFM  SSAEADKFVAAVKEMHERAKELGPLA |
| 77 |  |  | gi|45047811|emb|CAF30938.1| coenzyme F420-reducing hydrogenase subunit alpha [Methanococcus maripaludis] | >ORF 8083 and organism AE009439.seq  LAEGAVEIQPTTRHEGHAKLVLYVDDEGYVERAFYLNTSAVRGFEALAKGRPAEFVQVAVMRICGICQAT  HGTASAEAFERAMGIEPPKDGKLLRELCALGNRIQSHVLHQLLVLDDFVEDESEKVEAVKRIQQIRRIGQ  YVVDVVGGEGIHPPNIRIGGMAENISEAARRKLYRRLREARELMMEQHEFMVNIVERFGDENDLDIDEFG  RHDQPFLATHPTYGDPDRLDMDRVVELLPIEYYGEEHKEVAYQHRGQIPLYDGVPVEVGPRARYILFDGV  DPRGVLYIHVLRSQETLAAIDRAMTILDELNTSGKTLAEWEPKAGVGIGVHEAPRGTNVHIAKVNEKGIV  EDYRIIAASTWNFPVVEKAIEGENEEYAEVIMRCYDIUASCAAHVVKEVRDADSREKIRESVVKLA |
| 78 |  |  | >gi|39576202|emb|CAE80367.1| selenide, water dikinase [Bdellovibrio bacteriovorus HD100] | >ORF 6088 and organism AE009439.seq  MSRKKSLVEMADLHGUACKLPQGDLEDLLKGVELPEEGGRVEVGVGDDAAVIRVDGGYVIQSVDFFTPIH  PDPYTQGRIAANNSINDVFAMGATEVLSVLVVSGFPRELPEEDAREMLQGFADQCREVDALIVGGHTIMN  PWPILGGCVTGFAERYVTVGGAEPGDVLYLTKPLGTQPAMAALRLPEDVRKQFLTDSELEEAVDLAVEVM  TEPLKDAAEAALEVGVHAMTDVTGFGLKGHAGEMAEASGVRVVIERLPVIPGTTELSRALGYGLERGESA  ETAGGLLVAVPEEHAEDLEDAFERRDVWYRRIGRVEEGSGVEVRGDVEEVEDYP |
| 79 | L77117 | *Methanococcus jannaschii* | gi|44921146|emb|CAF30381.1| heterodisulfide reductase subunit A [Methanococcus maripaludis] | >ORF 266 and organism L77117.seq  LIVISILGGESMSPRVGVFVCYCGANINGVVDCEAVRDFAEKLDGVVVAKTYPFMCADPGQNLIKEAIKE  YNLDRVVVAACTPKIHEPTFRNCIKEAGLSPYYLEFVNIREHCSFVHMNDREKATKKAMELVAGAVERAK  RLEDVPQKIVEVDKSCLIIGGGIAGIQAALDLGDQGYKVYLVEKEPSIGGRMAQLAKTFPTDDCALUILA  PKMVSVANHPNVELITYAEVKNVEGFIGNFEVTIEKKPRYVDENICTGCGACAAVCPIEVPNEFDLGLGT  RKAIYVPFAQAVPLVYTIDMDHCIRCGLCEKACGPGAIRYDQKPEEIKLKVGTIICAVGYDEFDATLKEE  YGYGVYDNVITTLELERMINPAGPTGGHEIRPSDGKHPHRVVFIQCVGSRDAKVGKHYCSRICCMFALKN  AQLIKQHDPSTEVYICYMDIRSFGKGYEEYYRRAQEQFGVKFIRGRPACIMEDPETKNLIVRVEDTLLGE  IVEIEADLVVLSAGLSPRPDNPKLAKMLGLELSPDGFFKELHPKLAPVNTKVDGIAIAGVAQGPKDIPDT  VAQAKGAASAVSIPMAQGQFRIEMIRAVVDEDVCGGCQVCAKMCPYNAITYVEKDGHLVAQVNDVACKGC  GSCAGACPSGAMQLRYYRDEQIISFIDGVLEAHQKLES |
| 80 |  |  | gi|45047811|emb|CAF30938.1| coenzyme F420-reducing hydrogenase subunit alpha [Methanococcus maripaludis] | >ORF 318 and organism L77117.seq  LEVNFVTNRIEIAPTTRHEGHAKLILEVDEEGIVNKAYYLNTTPVRGFETMLKGKPAEFAPIAVMRICGI  CQTTHGIASCEAIENAIDCEVPDDGLLLRELVGIGNRLHSHPLHHLLTIDDFLKPDETDLKIELIKLIQR  MRKVGQLVVDIVGGEGIHPPNIVIGGMRTNITERAKSRLYYALRQYEKDAYELYEKYTELIERYLEEIGI  PDLGAHEYPYIATHTTYGDRYAINWDDVTEIPAQRYYDDEEAKQTTTIQIPLYAGVPAEGGPRARMVKFG  NFREGGSAMDINIARAQENLGAVYRALEILDELDLNGKTRAEVEYKDGFGIGVHEAPRATNTHMAEVGKD  GKIKSYRIIAASTWNFPIVEKAIEGYPQQYAEVIMRAYDIUASCATHVIVKDEETKEIIEVRKML |
| 81 |  |  | gi|50875900|emb|CAG35740.2| methyl-viologen-reducing hydrogenase, delta subunit [Desulfotalea psychrophila LSv54] | >ORF 429 and organism L77117.seq  VPSSKLNYKFNSHVVIEFIKLIIYRGISMDPVIIAFCCYQ**U**GYGAADLAGTSRMQYPATVRIVRLPCTGK  FDITYALRAFQKGADAVMVVG |
| 82 |  |  | gi|2622240|gb|AAB85625.1| methyl viologen-reducing hydrogenase, delta subunit� [Methanothermobacter thermautotrophicus]  gi|44921142|emb|CAF30377.1| coenzyme F420-non-reducing hydrogenase subunit delta [Methanococcus maripaludis] | >ORF 430 and organism L77117.seq  LAGTSRMQYPATVRIVRLPCTGKFDITYALRAFQKGADAVMVVG**U**KKGECAYETGNLKAEERVRFAKQLL  DELGIGGDRIDMFFMSAAEADKFVSAVNEMTARVEKLGPNPLKAQ |
| 83 |  |  | gi|2622673|gb|AAB86026.1| formate dehydrogenase, alpha subunit homolog [Methanothermobacter thermautotrophicus]  gi|45048129|emb|CAF31247.1| tungsten containing formylmethanofuran dehydrogenase subunit B [Methanococcus maripaludis] | >ORF 431 and organism L77117.seq  MVKVVRNVVCPFCGTLCDDLEILVEDNHIVGTRHACRIGNAKFMHFEGAVRYTEPLMRENKKDDFKKVDY  ETAIEETARLLTEATLPLIYGWSATECHAHMYGVELAELVGAVIDNTASVUHGPSLLAVQDVGYPVCTLG  EVKNRADVIIFWGSNPMHAHPRHMSRYSVFARGFFRERGREDRTLIVVDPRETDTAKLADIHLQVEPHKD  YELVSAMRAVLKGFELQVDKVAGVPADLIYEAVEVCKNAQFGELFFAMGVTMTRGKHRNIDNAIQLVIDL  NAYTKFGLMPMRGHYNVNGFNQVLTWVTGYPFGVDFSRGYPRYNPGETTANDLLQRGETDMMLNIASDPG  AHFPQKAVQHMAKIPLVCIDPHETPTTQLANIIIPPAIAGVEVEGTAYRMDGVPIQLRKVIDPPEGVLPD  REILKILIKKVKEML |
| 84 |  |  | gi|26108424|gb|AAN80626.1|AE016761\_201 Selenide,water dikinase [Escherichia coli CFT073] | >ORF 476 and organism L77117.seq  MERGNEKIKLTELVKLHGUACKLPSTELEFLVKGIVTDDDLLDKNILVGLGDDASIIKRNGLVIAKTVDV  FTPIVDDPYIQGKIAACNSTSDIYAMGLLDIVGVLAIVGIPEKLPIHVVREMLKGFQDFCRENKTTIVGG  HTILNPWPLIGGAVTGVGREEEVLTKAGVKVGDVLILTKPLGTQTAMALSRIPEEFKDLISITEEERDYI  INKAIEIMTTSNRYALKALRKAEERVGDKIANALTDITGFGILGHSNEMAKNSNVLIEINLLPCIKRTPE  LSRLFGHALLDGYGAETAGGLLISAKEEYKDNLIDELEKAKCYAFEVGRVVKKGEGKAVLSKDVKVIEI |
| 85 |  |  | gi|53758707|gb|AAU92998.1| HesB/YadR/YfhF family protein [Methylococcus capsulatus str. Bath] | >ORF 587 and organism L77117.seq  MKKVVISDEAKKFILDKLKKANQDKVVIYFEGFAUGGPKFGIAIAHPNENDKLIYDNEFKVYIDPIADQW  LDEVNISLRRSIFGKYLKIEGSSEC |
| 86 |  |  | gi|45047727|emb|CAF30854.1| formate dehydrogenase alpha subunit [Methanococcus maripaludis] | >ORF 859 and organism L77117.seq  MHKNKNLKELGETMEFKIVNTICPYCGVGCGLGLVVKDGRVIGIHPNKRHPINEGKLCAKGNYCYQFIHS  KDRLTKPLIKKESGFVETTWNKALEVIAENLKTYKDEIGFFSSARCTNEDNYILQKFARVALKTNNIDHC  ARLUHSATVTGMSACFGSGAMTNSIEDIELADCILIIGSNTFEQHPLIARRIMRAKDKGAKIIVIDPRRT  ITAKNSDIYLQIIPGTNVALINAMINVIIKENLIDKEFIKNRTEGFEKLKEIIKKYTPEYASKICGVDKE  LIIESAKIYGNAERASIIYCMGVTQFTHGVDAVKALCNLAMITGNIGKEGTGVNPLRGQNNVQGACDMGA  LPNVFPGYQKVEDGYKLFEEYWKTDLNPNSGLTIPEMIDESGKNIKFLYIMGENPIVSDPDVKHVEKALK  SLDFLVVQDIFLTETAKLADVVLPAACWAEKDGTFTNTERRVQLIRKAVNPPGEALEDWIIIKKLAEKLG  YGDKFNYNKVEDIFNEIRKVTPQYRGITYKRLKIDGIHWPCLDENHSGTKILHKDKFLTDNGRGKIFPVE  YREVAELPDKDYPFILTTGRIIFHYHTGTMTRRCKNLVEEINEPFIEINPDDAKSLKIENGDLVKVISRR  GEITAKARITEDIKKGVVFMPFHFVEANPNVLTNTALDELCKIPELKVCAVKIERI |
| 87 | BX950229 | *�Methanococcus maripaludi* | gi|2622673|gb|AAB86026.1| formate dehydrogenase, alpha subunit homolog [Methanothermobacter thermautotrophicus]  gi|19886584|gb|AAM01476.1| Formylmethanofuran dehydrogenase subunit B [Methanopyrus kandleri AV19] | >ORF 1117 and organism BX950229.seq  MQQNQKRSIIIEINHSHFFWVIKVEVFKNVVCPFCGTLCDDIEVLVENNHVVGTRNACRIGNAKFMHFEG  AIRHESPLMRENKKDDFKKVDYETATEETARLLVEAKLPLIYGWSSAECHAQQLGVLLAEKTKAIVDNTA  SVUHGPSLLAVQDVGYPVSTLGETKNRADVVLFWGSNPMHAHPRHMSRYSVFPRGFFRQRGKQDRQMIVV  DPRKTDTAKLADIHLQVEPHKDYELVSALRAAAKGFNIEAEQVAGVPTETIYEAVDICKNAQFGSLFFAM  GVTMSRGKHRIIDNAIQFVIDMNAYTKFVLTPMRGHYNVNGFNQVSTWVTGYPYGVDFSRGYPRYNPGET  ASNDVLQRGDTDMMINVASDAGAHFPQKAVQHMAKIPLVCIDPHETPSSVISNIVLPPAITGLEVSGTAY  RMDGVPIELRKVIKAPEGMLSDAEIMKMLIKKVDEMK |
| 88 |  |  | gi|2622673|gb|AAB86026.1| formate dehydrogenase, alpha subunit homolog [Methanothermobacter thermautotrophicus] | >ORF 1196 and organism BX950229.seq  MELDFIHTICPYCGTGCGVDLVVKDGTLVGTNPFKRHPVNEGKTCIKGSYCHEFVHRDDRLKTPLIRKNG  ELVEASWDEALELISGKLQNYSPEEVGFFSSARCTNEDNYVFQKFARTVIKTNNVDHCARLUHSATVVGL  GQAFGSGAMTNSISDIEDADCIFIIGSNTFEQHPLIARRVVRAKEKGTKIIVIDPRYTPTAKQADLYLQL  LPGTNIAVLNAIMHVLVKENLVDEEFIKNRTKGYEELKTTLETYTPEYASKLSGVAPELIVEAAKMYGSA  NAASILYCMGITQFTTGVNNVKSCCNLAMITGNIGKPGTGVNPLRGQNNVQGACDMGALPNVFPGYQAVP  ANHEKYAEAWNTCVDPNVGLSIPDMLAKAGEQVKCIYVMGENPMVSDPDIHHVEHALKSLDLLIVQDIFL  TETAQVADVVLPGASWAEKDGTFSNTERRIQKINKAVDSPGEAIADWKIVKMLAEKMGQGELFNFNTAEE  VFQEIAKVTPQYAGVTYERLGVDGLHWPCKTCEDPGTPILHCEKCLTPDGLGNIFAIDYADPDEMADSEY  PMTLTTGRIIFHYHTGTMTRRSKHMADEINEGFVEIHPEDAEKMGIKNKQKVKVSTRRGEVVVNAKITPN  IKQGVVFMPFHFAETAANILTNPAQDPNCKIPEYKVCAAKVEKI |
| 89 |  |  | gi|2622240|gb|AAB85625.1| methyl viologen-reducing hydrogenase, delta subunit� [Methanothermobacter thermautotrophicus]     gi|39981962|gb|AAR33424.1| heterodisulfide reductase subunit [Geobacter sulfurreducens PCA] | >ORF 1115 and organism BX950229.seq  LAGTSRMQYPASVRAIRVPCTGKFDITYALRAFQKGADAVFVAGUKPNECAFETGNFKAEERVKFGKQIL  DELGIGGERLEMFFMSGADAGKFTEAVKEMTDRVKKLGPNPIKA |
| 90 |  |  | gi|2622673|gb|AAB86026.1| formate dehydrogenase, alpha subunit homolog [Methanothermobacter thermautotrophicus] | >ORF 559 and organism BX950229.seq  MTEFKVVHTICPYCGTGCGIDLVVKDGKVVDSHPFKRHPVNEGKVCIKGNYCYEFVHSEDRLTKPLIKKN  GEFIEATWDEALDLIAGKLKQYSPDEVAFFSCARGTNEESYALQKFARTVLKTNNVDHCARIUHAPTVVG  LGECFGSGAMTNSITDLAQADVLLIYGSNTFEAHPLIARSIVKAKENGTKIIAIDPRTTHTAKMADLHLK  LIPGSNIDLINTITNIIIQEGMADEEFIKNRTEGYDELKDVVSKYTLEKTAELSGIPAETILEAARMYGS  AENASIMYCLGVTEYTFGVDNVKSCCNLAMVTGNLGRPGTGVNPLRGQNNVQGACDMGALPNVFPGYQKV  GEAYERLENLWETADLNREIGLTSPEVLHKAGEQVKFLHIVGEDPMVADADINHVEKALKSLDFFVVQDI  FLTETAKLADVVLPAACWAEKDGTFTNSERRVQRIRKAVDAPGDALPDWLIVRKLAEKMGAGEKLNFESA  SEIFDEMAKVIPQYAGMSFERLGIDGLQWPCKTPEDPGTPILHKEKFLRPNGLGKFTPVEHKDADELIDE  EYPLILTTGRIIFHYNSGTMTRRCDSITNEIDENFIEINTEDAKELGIKPGEKVRVSSRRGTVNADARVT  ENVIKGVVYMSFHFLEEATNKLTNSAYDPVSKTAELKICAVKVEKI |
| 91 |  |  | gi|2622673|gb|AAB86026.1| formate dehydrogenase, alpha subunit homolog [Methanothermobacter thermautotrophicus]  gi|19918286|gb|AAM07526.1| formylmethanofuran dehydrogenase, subunit B [Methanosarcina acetivorans str. C2A] | >ORF 630 and organism BX950229.seq  MASQTFKDIVCPVCGGACDDIEIVWDEEKRDLTVRNACKLGAAKFKEIISHHRIMSPQIRKNGVLVDVSW  EEALEKAAEILANSKRPLLYMGAETSCEAMTTGLHMGEYLGGIVDSCSTVUHGPSLMGVQEAGKAGSTAG  ETKNRADVVIYWGTNPMDSMPRHLSRYGVFPRGYFVEKGRNSRTVITIDPRKSATAKASDIHLQLNPSTD  YELFSAFMMASRGKRPHPSIEKVTGIPVDTIMETVELIKNAKFASIYGGLGLASSFGKQRNIECVMTLVK  ELQRYTKVTIGLIRGHCNVAGFNVLASYLYGFPFGLDFAKGYPRYNPGEFTANDVLREKEVDCAFIMASD  VGAHYPQDSVSHLKNIPVITLDIAPCPSTSVADVVLPGVIDALECDGTFYRFDEIPIYYKPFAKSPFDFT  KSNEDTMEQLFELVKEIKERNGQ |
| 92 |  |  | gi|19886593|gb|AAM01482.1| Heterodisulfide reductase, subunit A, polyferredoxin [Methanopyrus kandleri AV19] | >ORF 827 and organism BX950229.seq  MSDPKVGVFVCYCGANINGAVDCEAVKDFASELDGVAVAATYPFMCADPGQGLIKDAIKEHGLDRIVVAA  CTPKIHEPTFRGCLQDAGISPYYLEFVNIREHDAFVHMGDVEGATRKACEMIAGGVERAKKLEDVPQKVV  DVDKSCMVIGAGIAGIQSALDLGDQGFKVYLVDKDESIGGRMAQLAKTFPTDDCAMUILAPKMVSAANHP  NIELITFAEIKNIDGYIGNFDVTLEKKPRYVDEDTCTGCGACAAACPIEVPNEFDLGLGTRKAIYVPFPQ  AVPLLYTIDKEHCIDCGLCAKVCCAEAVRYDQKPQELNIKVGTIITATGYDEFDATKKEEYGYGVYDNVI  TTLEVERMINPAGPTHGHEIRPSDGKAPKRTVYIQCVGSRDEKVGNPYCSRVCCMFALKNAQLMKMHDPN  AEVYICYMDIRAFGKGYEEYYKRAQDQFGVKFIRGRPANIFEDPETKNLTVRVEDTLMGEILEIDADLVV  LSAGLEAKKDAGELAKMLGIDRGPEGFFKELHPKLAPVNTKVDGIAIAGVAQGPKDIPDTVAQAKGAASA  VAIPMSQGQFKIEMIRATVNEEVCGGCKVCALMCPYNAITYEEKDGHLVAITDDVACKGCGACAAACPSG  AMQLRYYRDEQVIGMIDGILNAAKMLEE |

 

 

**Master list: Candidate selenoprotein ORFs with an
in-frame UGA codon that aligns with conserved D/E/S etc.**

 

|  |  |  |  |  |
| --- | --- | --- | --- | --- |
|  | **Accession ID** | ***Organism*** | **Functional**  **Assignment** | **Sequence** |
| 1 | AE004439 | *Pasteurella multocida* | gi|5105712|dbj|BAA81024.1| 280aa long hypothetical protein [Aeropyrum pernix K1] | >ORF 1591 and organism AE004439.seq  MIMVRRSGPILILSFAFSNSSIVTKRLLVRAANNAASLTKLAKSAPEYPGVPRASVIASTSGAIGTRRIC  TFKICSRPRTSGKPTTT**U**RSKRPGRVNAGSSTSGRFVAAITITPSFPSKPSISTNIWFNVCSRSSCPPPK  PAPR |
| 2 | AE005674 | *Shigella flexneri 2a* | gi|12519413|gb|AAG59567.1|AE005669\_11 putative lipoate-protein ligase A [Escherichia coli O157:H7 EDL933] | >ORF 11616 and organism AE005674.seq  MARTKLKFRLHRAVIVLFCLALLVALMQGASWFSQNHQRQRNPQLEELARTLARQVTLNVAPLMRTDSPD  EKRIQAILDQLTDESRILDAGVYDEQGDLIARSGESVEVRDRLALDGKKAGGYFNQQIVEPIAGKNGPLG  YLRLTLDTHTLATEAQQVDNTTNILRLMLLLSLAIGVVLTRTLLQGKRTRWQQSPFLLTASKPVPEEEES  EKKE**U**PITTRKEIVMSTLRLLISDSYDPWFNLAVEECIFRQMPATQRVLFLWRNADTVVIGRAQNPWKEC  NTRRMEEDNVRLARRSGGGGAVFHDLGNTCFTFMAGKPEYDKTISTSIVLNALNALGVSAEASGRNDLVV  KTVEGDRKVSGSAYRETKDRGFHHGTLLLNADLSRLANYLNPDKKKLAAKGITSVRSRVTNLTELLPGLT  MSRFARP |
| 3 | AE014073 | *Shigella flexneri 2a* | gi|12519413|gb|AAG59567.1|AE005669\_11 putative lipoate-protein ligase A [Escherichia coli O157:H7 EDL933] | >ORF 11527 and organism AE014073.seq  MARTKLKFRLHRAVIVLFCLALLVALMQGASWFSQNHQRQRNPQLEELARTLARQVTLNVAPLMRTDSPD  EKRIQAILDQLTDESRILDAGVYDEQGDLIARSGESVEVRDRLALDGKKAGGYFNQQIVEPIAGKNGPLG  YLRLTLDTHTLATEAQQVDNTTNILRLMLLLSLAIGVVLTRTLLQGKRTRWQQSPFLLTASKPVPEEEES  EKKE**U**PITTRKEIVMSTLRLLISDSYDPWFNLAVEECIFRQMPATQRVLFLWRNADTVVIGRAQNPWKEC  NTRRMEEDNVRLARRSGGGGAVFHDLGNTCFTFMAGKPEYDKTISTSIVLNALNALGVSAEASGRNDLVV  KTVEGDRKVSGSAYRETKDRGFHHGTLLLNADLSRLANYLNPDKKKLAAKGITSVRSRVTNLTELLPGLT  MSRFARP |
| 4 | AE006469 | *Sinorhizobium meliloti* | gi|5105084|dbj|BAA80398.1| 123aa long hypothetical protein [Aeropyrum pernix K1] | >ORF 2508 and organism AE006469.seq  MASTGPVSTICPAYMTATR**U**QRPATMPRSWVIRTIANPIFSCNSARSSRIWAWIVTSRAVVGSSAIRSLG  SQAIPMAIMTRCRMPPENSCGY |
| 5 | AE008691 | *Thermoanaerobacter tengcongensis* | gi|56379161|dbj|BAD75069.1| hypothetical conserved protein [Geobacillus kaustophilus HTA426] | >ORF 2809 and organism AE008691.seq  MAMLGTGKTHLATAIGVEACKKGYNVKFFRTAALVNRLVEARKGGELSGFLKQLSKADLLMCDEWGYVPL  DQEGAQLLFQVISDCYEQKSIIITTNLEFS**U**WVNIFYDEQMTAAIIDRLIPHCYLLIFDGQSYRMKQSLM  KQLS |
| 6 | BA000007 | *Escherichia coli O157H7* | gi|24052006|gb|AAN43255.1| orf, conserved hypothetical protein [Shigella flexneri 2a str. 301]  gi|56127849|gb|AAV77355.1| putative oxidoreductase [Salmonella enterica subsp. enterica serovar Paratyphi A str. ATCC 9150] | >ORF 12678 and organism BA000007.seq  MVQRITIAPQGPEFSRFVMGYWRLMDWNMSARQLVSFIEEHLDLGVTTVDHADIYGGYQCEAAFGEALKL  APHLRERMEIVSKCGIATTAREENVIGHYITDRDHIIKSAEQSLINLATDHLDLLLIHRPDPLMDADEVA  DAFKHLHQSGKVRHFGVSNFTPAQFALLQSRLPFTLATNQVEISPVHQPLLLDGTLDQLQQLRVRPMAWS  CLGGGRLFNDDYFQPLRDELAVVAEELNAGSIEQVVYAWVLRLPSQPLPIIG**U**GKIERVRAAVEAETLKM  TRQQWFRIRKAALGYDVP |
| 7 |  |  | gi|24053117|gb|AAN44217.1| PTS system, glucitol/sorbitol-specific IIB component and second of two IIC components [Shigella flexneri 2a str. 301] | >ORF 7410 and organism BA000007.seq  LRKRWAFNLNKKFIWQEQHHDAYSDRKRNGGLGRPA**U**AGNHAGQKNRLYHPAGTRPAIVDKLAQLTGWQA  IDGFKEGEPAEAEIGVAVIDCGGTLRCGIYPKRRIPTINIHSTGKSGPLAQYIVEDIYVSGVKEENITVV  GDATPQPSSVGRDYDTSKKITEQSDGLLAKVGMGMGSAVAVLFQSGRDTIDTVLKTILPFMAFVSALIGI  IMASGLGDWIAHGLAPLASHPLGLVMLALICSFPLLSPFLGPGAVIAQVIGVLIGVQIGLGNIPPHLALP  ALFAINAQAACDFIPVGLSLAEARQDTVRVGVPSVLVSRFLTGAPTVLIAWFVSGFIYQ |
| 8 |  |  | gi|22777319|dbj|BAC13592.1| transposase in Marinococcus halophilus [Oceanobacillus iheyensis HTE831] | >ORF 3692 and organism BA000007.seq  VIVIQCRLGTRFCVKRLLLKVSIKAVIKLDNKVTHCYSLVLKGCIVN**U**IASSHRPDFNALSGASEAAYLH  DRMSAKGRCYDNARAESFFHSLKVECIYG |
| 9 | AE017042 | *Yersinia pestis biovar Mediaevails* | gi|12515969|gb|AAG56892.1|AE005412\_2 orf, hypothetical protein [Escherichia coli O157:H7 EDL933]  Probably yes | >ORF 5947 and organism AE017042.seq  LSKISSLSFQPCGHHASQCDIFLSITLFLCKFQINQCNSLLDCSFGHWICPRWMC**U**EIDSIELMRHFITH  FGRRDDIDNIKNLFDDQLASDQIRD |
| 10 | AE017180 | *Geobacter sulfurreducens* | gi|33237401|gb|AAP99469.1| Membrane carboxypeptidase (penicillin-binding protein) [Prochlorococcus marinus subsp. marinus str. CCMP1375]  >gi|46450120|gb|AAS96769.1| monofunctional biosynthetic peptidoglycan transglycosylase [Desulfovibrio vulgaris subsp. vulgaris str. Hildenborough]  >gi|6457783|gb|AAF09708.1|AE001874\_5 acetyl-CoA carboxylase, bitoin carboxyl carrier protein [Deinococcus radiodurans] | >ORF 142 and organism AE017180.seq  VRMKKLLYLAIGAVVAYGIYIAISLMFLPSVAELKNRRTTMTIQVKDWHGEYHPFTVGPKNRYWTPSGSI  PPEMKWAVILAEDANFYKHEGIDVKAIKNAIKYDLEKKSFARGASTITQQVAKNLFLSREKTISRKIKEI  VLAKRMEEELTKGRIIELYLNVVELGPMVYGIGHGARYYFGKPASALTPRECAFLAAMLPGPRVAYNPYK  NLGKVLKRSDMILRLLRGKGVLSDDEYRQALAQTPNIAGLQRKVDASIEKEETTFENRTGATVPLEPQST  TAPDEQAPEEVPAASSQPAANGEPAAGDGGEQQSPPPAR**U**GSRTCSDCCPLVRQSERRKKGVTDCSGHPF |
| 11 |  |  | >gi|18144401|dbj|BAB80447.1| probable cytochrome C-type biogenesis protein [Clostridium perfringens str. 13] | >ORF 1728 and organism AE017180.seq  LWWIKMGLYSRRSWGACSGTTPT**U**SPISTAPWPSRAPRQVEVFMESTNITFVGAFVAGLLSFLSPCVLPL  IPSFITYITGLSFADIQSEHPTHKVRQQTIVHSLLFIAGFTFVFVLLGASATFIGGFLHEHMNVIRKVGG  ALIVIFGIHVSGLVPIHLLLGEKRLQVHRKPAGYLGSFLVGLAFAAGWTPCIGPILASILMVAATEETVT  KGILLLFTYSMGLAIPFFLSSLAMHQFLTFFNRFKKHIRILEIVTGLFLVVVGVMIFTNYLSVLSRYTMK  WFGGM |
| 12 | AE017226 | *Treponema denticola ATCC 35405* | gi|39983625|gb|AAR35018.1| ABC transporter, ATP-binding protein [Geobacter sulfurreducens PCA] | >ORF 3136 and organism AE017226.seq  MQQMLGIWTAASTLLWKP**U**CPPADQVIDVLSGGEKRRVALCRLLLQKPDILLLDEPTNHLDAETVAWLER  HLHQYAGTIICVTHDRYFLDNVAGWILELDRGEGIPWKGNYSSWLDQKQKRLALEEKGETERQKALKREL  EWIGMSPKGRHAKSKARINEYEKLLAQGSKEKIKDSQITIPPGPRLGNLVIDVKNAAKHYGDRILFDKLN  FSVPAGAIVGIIGPNGAGKTTLFKMIVGAAGFETPEGADQKRQIVKPDEGEIKIGDSVKLCYVDQTREKL  DPNKTVWEQLSDGLDIIKLGASDGSSGVREVNSRAYCSWFNFSGQDQSRKVGVLSGGERNRLNLAMMLKE  GGNVLMLDEPTNDLDVTTLRALEEALESFAGSVLVISHDRWFLDRVCSHILAFEADGEVVWFDGNWTEYA  EWRREKYGKDADTPHRGVYRKLER |
| 13 | AL513382 | *Salmonella typhi* | gi|24053123|gb|AAN44223.1| putative 2-component transcriptional regulator [Shigella flexneri 2a str. 301] | >ORF 10316 and organism AL513382.seq  LQKVVLLIAGSVPTLHLIDLYEIIIILYFWYSQKDYK**U**CCQYDTIDCQNDNEVTMSFSVEVLAGIAIELQ  RGIGHQDRFQRLITTLRQVLACDASALLRYESRQFIPLAIDGLAQDVLGRRFTLEGHPRLEAIARAGDVV  RFPADSDLPDPYDGLIPGQESLKVHACVGLPLFAGQNLIGALTLDAMTPEQFEVFSDEELRLVAALAAGA  LSNALLIEQLESQNMLPGSSGVFEPIKETHMIGLSPAMTQLKKEIEIVAGSDLNVLIGGETGTGKELVAK  AIHQGSPRAVNPLVYLNCAALPESVAESELFGHVKGAFTGAISNRSGKFEMADNGTLFLDEIGELSLALQ  AKLLRVLQYGDIQRVGDDRSLRVDVRVLAATNRDLREEVLAGRFRADLFHRLSVFPLFVPPLRERGDDVV  LLAGYFCEQCRLRLGLSRVVLSPGARRHLLNYGWPGNVRELEHAIHRAVVLARATRAGDEVVLEEQHFAL  SEDVLPAPSAESFLALPACRNLRESTENFQREMIRQALAQNNHNWAASARALETDVANLHRLAKRLGLKD |
| 14 |  |  | gi|28854727|gb|AAO57790.1| fumarate hydratase, class I, putative [Pseudomonas syringae pv. tomato str. DC3000] | >ORF 3896 and organism AL513382.seq  LRRFTSGGLNRSSLSRCRCCHVSFCCAIMTTSSVCGVARRRKSGRS**U**KRNARKIELELRLEEGLNRLGIG  PQGLTGNSSVIGVHIESAARHPSTIGVVRRDFRPDFPGAADRLQAVVDAHRFAVNPLGRHDVAPFFRFYA  AGSSASGQRGRTVTPRSAVTPALNRTIPA |
| 15 | L42023 | *Haemophilus influenzae* | gi|52629104|gb|AAU27845.1| cell division protein FtsK [Legionella pneumophila subsp. pneumophila str. Philadelphia 1] | >ORF 909 and organism L42023.seq  MLPILFLSPLFSCLFIY**U**KQKQLNSFPVLELFYGSFGFTMLIIGLCVVSMLLLSSNTFYLSGGVLGGSLV  VNWFYPVLGKFGSILIGFVLALIGFIFCSGTSLIRLIVTFYHWLTMKNEQSENAEQEKSTEELEQIVIVK  SDRSETENLDQNYLNVEQNSEIETVKPSLEAENISIGKSSSHLINISGLNPEVSIKSEYELANEENEKPQ  FSFGFDSESLPSVNLSSDSDEQRVSKNDFVAVWNKPVKTVVQEDLAIKSKCG |

 

 

**Master list:� PYRROLYSINE-containing methyltransferase
proteins (These predictions are lower confidence than the selenoprotein
predictions)**

 

|  |  |  |  |
| --- | --- | --- | --- |
| **Accession ID** | ***Organism*** | Functional  Assignment | **FASTA** |
|  |  |  |  |
| AE008384 | *Methanosarcina mazei* | AE008384.1004.seq:>gi|19914316|gb|AAM03972.1| trimethylamine methyltransferase [Methanosarcina acetivorans str. C2A] | >ORF 1004 and organism AE008384.seq  MAKCNAVAGFNALNGVQLNLFTTDELKAIHYATMEVLMDPGIQVSDPEARQIFKENGCEVNEQTNVVKIP  EYLVRRALQLAPSRFVLWGRDKKYNTVQEAGGKVHWTCFGTGVKMCKYQEGKYVTVDSVEQDIADIAKLC  DWAENIDYFSLPVSARDIAGQGAQDVHETFTPLTNTAKHFHHIDPVGENVEYYRDIVNAYYGGDEEEARK  KPIFSMLLCPTSPLELSVNACQVIIKGARFGMPVNVLSMAMSGGSSPVYLAGTLVTHNAEVLAGITLAQL  TVPGAKVWYGSSTTTFDLKKGTAPVGSPELGLISASVAKLAQFYGLPAFVAGT**U**SDAKIPDNQAGHEKTM  TCLLPALAGANTLYGAGMLELGMTFSMEQLVIDNDIIKMTKKALQGVPVNEETLAVESIQKVGIGNNFLA  LKQTRQLVNYPSDPMLIDRRMFGDWAAAGSKDLAAAAHEKVVDVLKNHVVKPIDADILKDMKAVVDKADK  AFRGM |
|  |  | AE008384.2293.seq:>gi|19914753|gb|AAM04365.1| trimethylamine methyltransferase [Methanosarcina acetivorans str. C2A] | >ORF 2293 and organism AE008384.seq  MAQNNAVAGFNALNGVELSLFTTDELKAIHYATMEVLMNPGVQVSDPEARQIFKENGCEVDEKTSIVKIP  EYLVRRALQLAPSRFVLWGRDKKYNTVQEAGGKVHWTCFGTGVKMCKYQDGKYVTVDSVEQDIADIAKLC  DWAENIDYFSLPVSARDWAGKGAQDVHETLTPIANTAKHYHHIDPVGEHVDYYRDIVKAYYGGDEEEARK  KPIFSMLLCPTSPLELSVNACQVIIRGARFGMPVNVLSMAMSGGSSPVYLAGTLVTHNAEVLSGIVLAQL  TVPGAKVWYGSSTTTFDLKKGTAPVGSPELGLISAAVAKLAQFYGLPSYVAGT**U**SDAKIPDNQAGHEKTM  TCLLPALAGANTIYGAGMLELGMTFSMEQLVIDNDIIKMVKKAMQGIPVSPETLAVESIQKVGIGNNFLA  LKQTRMLVDYPSSPMLIDRRMFGDWAASGSKDLAAVANEKVQDILKNHQVPPVDADILKDMQAIVDKADR  AFKEG |
|  |  | AE008384.1005.seq:>gi|19914320|gb|AAM03976.1| dimethylamine methyltransferase [Methanosarcina acetivorans str. C2A] | >ORF 1005 and organism AE008384.seq  LPCGRQGLSCPNSEPGIQLSDLILKQYKWRIKMATEYALRMGDGKRIFLTKDKIIEELEAGMANASDLGE  IPDLSGDEIDKLAEILMMPGKAVSVEQGMEVPVTHDIGTLRLDGDQGNSGVGIPSSRLVGCMMHERAFGA  DTMELGHIDYSYKPVKPVVANECQAMEVCQQNMIIPLFYGAMPNMGLYYTPDGPFENPGDLMKAFKIQEA  WDSMEHAAAHLTRDTVWVMQKLFASGADGVNFDTTAAAGDADMYGTLHAIEALRKEFPDMYIEAGMAGEC  VLGMHGNLQYDGVTLAGLWPHQQAPLIAKAGANVFGPVCNTNTSKTSPWNLARAVNFMKAAVQASSIPCH  VDMGMGVGGIPMLETPPIDAVTRASKAMVEIAGVDGI**U**IGVGDPLGMPISHIMASGMTGMRAAGDLVARM  QFSKNMKIKEAKEYVAKKLNVETMDLADEYVMRELREELDIGVITSVPGAAKGIAAKMNIEKLLDVKINS  CNLFRKQTR |
|  |  | AE008384.2415.seq:>gi|19913899|gb|AAM03597.1| monomethylamine methyltransferase [Methanosarcina acetivorans str. C2A] | >ORF 2415 and organism AE008384.seq  MTFRKSFDCYDFYDRAKVGEKCTQDDWDLMKIPMKAMELKQKYGLDFKGEFVPTDKDMMEKLFQAGFEML  LECGIYCTDTHRIVKYTEDEIWDAINNVQKEFTLGTGRDAVNVRKRSVGDKRKPIVQGGPTGSPISEEVF  MPVHMSYALEREVDTIVDGVMTSVRGKAPIPGSPYEVLAAKTETRLIKQACAMAGRPGMGI**U**GPETSLSA  QGNISSDCMGGQISSDSHEVSQLNELKIDLDAIAVIAHYKGNSDIIMDEQMPIFGGYAGGIEETTIVDIA  THINAFVMSSASWHLDGPVHIRWGSTNTRETLTIAGWACATISEFTDMLSGNQYYPCAGPCTEMCLLEAS  AQSITDTASGREILSGVASAKGVVTDKTTGMEARMMGEVARATAGMEISEVNKVLNALVPLYEKNYATAP  AGKTFQECYDVKTITPTEEYMQVYDGARKKLEDLGLVF |
|  |  | AE008384.2905.seq:>gi|19914755|gb|AAM04366.1| dimethylamine methyltransferase [Methanosarcina acetivorans str. C2A] | >ORF 2905 and organism AE008384.seq  MATEYALRMGDGKRVFLAREKIMEEIEAGTANAADLGEIPALSADEMNKLAEILMMPGKAVSVEHGMEIP  VTHDIGTIRLDGDQGNSGVGIPSSRLVGCMMHERAFGADTMELGHIDYSFKPVKPVVANECQAMEVCQQN  MIIPLFYGAMPNMGLYYTPDGPFENPGDLMKAFKIQEAWDSMEHAAEHLTRDTIWIMQKLFASGADGVNF  DTTAAAGDGDFYGTLHAIEALRKEFPEMYIEAGMAGEMVLGMHGNLQYDGVTLAGLWPHQQAPLVAKAGA  NVFGPVVNTNTSKTSPWNLARAVTFIKEAVKVSSLPCHVDMGMGVGGIPMLETPPIDAVTRASKAMVEIA  GVDGI**U**IGVGDPLGMPISHIMASGMTGMRAAGDLVARMQFSKNMKIKEAKEYVAKKLNVEIRDLADEYIM  RELREELNIGVITSVPGSAKGIAAKMNIEKLLGIKINSCELFRKQTGK |
|  |  | gi|19914320|gb|AAM03976.1| dimethylamine methyltransferase [Methanosarcina acetivorans str. C2A] | >ORF 607 and organism AE008384.seq  MATEYALRMGDGKRIFLTKDKIMEELEAGMANASDLGEIPDLSGDEIDKLAEILMMPGKTVSVEQGMEVP  VTHDIGTLRLDGDQGNSGVGIPSSRLVGCMMHERAFGADTMELGHIDYSYKPVKPVVANECQAMEVCQQN  MIIPLFYGAMPNMGLYYTPDGPFENPGDLMKAFKIQEAWDSMEHAAAHLTRDTVWVMQKLFASGADGVNF  DTTAAAGDADMYGTLHAIEALRKEFPDMYIEAGMAGECVLGMHGNLQYDGVTLAGLWPHQQAPLIAKAGA  NVFGPVCNTNTSKTSPWNLARAVNFMKAAVQASSIPCHVDMGMGVGGIPMLETPPIDAVTRASKAMVEIA  GVDGI**U**IGVGDPLGMPISHIMASGMTGMRAAGDLVARMQFSKNMKIKEAKEYVAKKLNVETMDLADEYVM  RELREELDIGVITSVPGAAKGIAAKMNIEKLLDVKINSCNLFRKQTR |
|  |  | gi|19913899|gb|AAM03597.1| monomethylamine methyltransferase [Methanosarcina acetivorans str. C2A] | >ORF 675 and organism AE008384.seq  MTFRKSFDCYDFYDRAKVGEKCTQDDWDLMKIPMKAMELKQKYGLDFKGEFVPTDKDMMEKLFQAGFEML  LECGIYCTDTHRIVKYTEDEIWDAINNVQKEFTLGTGRDAVNVRKRSVGDKRKPIVQGGPTGSPISEEVF  MPVHMSYALEREVDTIVDGVMTSVRGKAPIPGSPYEVLAAKTETRLIKQACAMAGRPGMGI**U**GPETSLSA  QGNISSDCMGGQISSDSHEVSQLNELKIDLDAIAVIAHYKGNSDIIMDEQMPIFGGYAGGIEETTIVDIA  THINAFVMSSASWHLDGPVHIRWGSTNTRETLTIAGWACATISEFTDMLSGNQYYPCAGPCTEMCLLEAS  AQSITDTASGREILSGVASAKGVVTDKTTGMEARMMGEVARATAGMEISEVNKVLNALVPLYEKNYATAP  AGKTFQECYDVKTITPTEEYMQVYDGARKKLEDLGLVF |
| AE010299 | *Methanosarcina acetivorans* | gi|56678713|gb|AAV95379.1| trimethylamine methyltransferase family protein [Silicibacter pomeroyi DSS-3] | >ORF 1195 and organism AE010299.seq  MAKNNAVAGFNALNGVELNLFTTDELKAIHYATMDVLMNPGVQVSDPEARQIFKENGCEVDEKTNVVKIP  EYLVRRALQLAPSRFVLWGRDKKFNTVQECGGKVHWTCFGTGVKMCKYQDGKYVTVDSVEQDIADIAKLC  DWAENIDYFSLPVSARDIAGQGAQDVHETLTPIANTAKHYHHIDPVGENVEYYRDIVTAYYGGDEEEARK  KPIFSMLLCPTSPLELSVNACQVIIKGARFGMPVNVLSMAMSGGSSPVYLAGTLVTHNAEVLAGITLAQL  TVPGTKVWYGSSTTTFDLKKGTAPVGSPELGLISASVAKLAQFYGLPAFVAGT**U**SDAKIPDNQAGHEKTM  TCLLPALAGANTLYGAGMLELGMTFSMEQLVIDNDIIKMTKKALQGVPVNEETLAVESIQKVGIGNNFLA  LKQTRQLVNYPSDPMLIDRRMFGDWAAAGSKDLASAAHDKVVDVLKNHVVKPIDADILKDMQAVVDRADK  AFRGM |
|  |  | >gi|14247242|dbj|BAB57633.1| menaquinone biosynthesis methyltransferase [Staphylococcus aureus subsp. aureus Mu50] | >ORF 3292 and organism AE010299.seq  LSIAIGEINSQEGIRILM**U**NFLEVKTMNTIKARLLNRKASSAKSRPDEIVKILSLKPGQKIADVGSGGGH  FSLLFARYVGSEGKVYATDTNKGFLDFVSNSSEKSDFHNIVTVLAEGDRFPFTNEKLDLVFLRNVYHHLP  NREVYFRDLAAALSTGTRVAIIDYDGRGKWSFHRLFCHSVPKETIINEMAVAGYCLVEDHTFLPEQSFLI  FSAANNKKNCNQEGTLF |
|  |  | >gi|20907142|gb|AAM32236.1| Conserved hypothetical protein [Methanosarcina mazei Goe1]  >gi|36785418|emb|CAE14364.1| Protein methyltranferase HemK (Protein-glutamine N-methyltransferase) [Photorhabdus luminescens subsp. laumondii TTO1] | >ORF 3612 and organism AE010299.seq  LKGIQIEYKNTRIKLGASDLVYEPAEDSFLLADTALEEA**U**PGMRILEIGVGSVFVSAVLRANVKDIRVLA  IEINPHVALCAKANGIEVIRTDLFRGSETGKFENFL |
|  |  | >gi|56679325|gb|AAV95991.1| trimethylamine methyltransferase family protein [Silicibacter pomeroyi DSS-3] | >ORF 4214 and organism AE010299.seq  MAQNNAVAGFSSLQGVELNLFTIDELKAIHYATMEVLMNPGVQVSDPEARQIFKENGCEVDEKTNVVKIP  EYLVRRALQLAPSRFVLWGRDKKYNTVQEAGGKVHWTCFGTGVKMCKYQSGKYVTVDSVEQDIADIAKLC  DWTENIDYFSLPVSARDWAGKGAQDVHETLTPIANTAKHYHHIDPVGENVEYYRDIVKAYYGGDEEEARK  KPIFSMLLCPTSPLELSVNACQVIIKGARFGMPVNVLSMAMSGGSSPVYLAGTLVTHNAEVLSGIVLAQL  TVPGAKVWYGSSTTTFDLKKGTAPVGSPELGLISAAVAKLAQFYGLPSYVAGT**U**ADAKIPDNQTGHEKTM  TCFLPALAGANTIYGAGMLELGMTFSMEQLVIDNDIIKMVKKAMQGIEVSPETLAVDSIQKVGIGNNFLA  LKQTRLLVNYPSDPMLIDRRMYGDWAASGSKDLAAVANEKVTDVLKHHEVPPIDTDILKDMQAIVDRADK  AFKES |
|  |  | >gi|20904823|gb|AAM30145.1| SAM-dependent methyltransferases [Methanosarcina mazei Goe1]  >gi|29607541|dbj|BAC71598.1| hypothetical protein [Streptomyces avermitilis MA-4680] | >ORF 815 and organism AE010299.seq  MARMIFMKSSQYDTAFVKENMMGPNSMKIIEEVAESLTLEKEMRVLDLGCGKGLTSIFLAKEYDATVFAT  DLWISATENYERIKSMGIEDKIIPIHAEAHDLLFAEEFFDVTISIDAYHYFGVEEDYLTKHLAPLVKRGR  KIAVAVPGLKKEFENGVPEEL**U**PYWLDDMSLTLHSYNWWYNLWKKSDTVSIKECKELKCLEEAWQEWLSC  DNDYARRDIGMMEVEGGNYFNLV |
|  |  | >gi|56312282|emb|CAI06927.1| conserved hypothetical protein,predicted methyltransferase [Azoarcus sp. EbN1] | >ORF 2690 and organism AE010299.seq  MSAMRFPVKTNTALTRTIPRSRGTSDIKPAATVARPIPG**U**EKTCSTRTAPPNSSLKLKNCRVMAGRATFL  TPCLKTRSSPFSPLAFANNT |
|  |  | gi|45047608|emb|CAF30735.1| SAM (and some other nucleotide) binding motif:Generic methyltransferase [Methanococcus maripaludis] | >ORF 5565 and organism AE010299.seq  VDEKGKSPGFPYIAEHIFAPIYPVIAAHIVKESGIKQGICLDLGCGIASLGIAVAELTDMQVYGVDFSTE  MCRLSKAKANRHFLSDKVVPLQADVHLLPFRDNSAVLIVSRGSVFFWKDLPVAFREISRVLAPGGQAWIG  GGFGTKELKAQISEKMVEIDPDWHTASK**U**RLSPETIRAIREAGERTEIPCHVVKDDSGFWVVLSKEK |
|  |  | gi|20905508|gb|AAM30766.1| Methylcobalamin: Coenzyme M methyltransferase [Methanosarcina mazei Goe1] | >ORF 6447 and organism AE010299.seq  MADPDLRDRFLNTLHGKAVDKVPVLAVTQTGTVELMRESGAAWPDAHFDAKKMADLALSAHTFAGLEAVR  YPFCLTVLSEALGCKVNPGR**U**DIQPSPGSNPFAKEPEKMELPSDFAQRGRIPLIKDVTSILREKAGEEVP  LIAGMEGPASLASRLLGTYNFLTWMIRRPETLSQCLKVTGATCSAYAEILSEAGADAVCIVDGIAGPDML  DPRHLEALIRPEYESFCKSGKGIKLIHVCGNSTPILKTLSRCGFQGISIEEKVTDLQTAKKLVGSKTKLI  GNLSSSGIMLNGTCEEIKLEAQKCLEDGIDILAPGCGIAPKTPIKNIRAMVEARDEYYLTGKIRVRTHHD  CASP |
|  |  | Predicted ORF monomethylamine methyltransferase [Methanosarcina mazei Goe1] | >ORF 699 and organism AE010299.seq  MTFKKSFDCYDFYDRAKVGEKCTQDDWDLMKIPMKAMELKQKYGLDFKGEFVPTDRDMMEKLFQAGFEML  LECGIYCTDTHRIVKYTEDEIWDAINNVQKEFTLGTGRDAVNVRKRSVGDKRKPIVQGGPTGSPISEDVF  MPVHMSYALEKEVDTIVNGVMTSVRGKPPVPKSPYEVLAAKTETRLIKQACAMAGRPGMAVUGPETSLSA  QGNISADCAGGMQSTDSHEVSQLNELKIDLDAIAVIAHYNANSDIIMDEQMPIFGGYAGGIEETTIVDVA  THINAFVMSNASWHLDGPVHIRWGSTNTRETLTIAGWACATISEFTDMLSGNQYYPCAGPGTEMCLLEAS  AQSITDTASGREILSGVASAKGVVTDKTTGMEARMMGEVARATAGAEITEINKILDKLVALYEKNYASAP  AGKTFQECYDVKTVTPTEEYMQIYDGARKKLEELGLVF |
|  |  | Predicted ORF monomethylamine methyltransferase [Methanosarcina mazei Goe1] | >ORF 6589 and organism AE010299.seq  MTFRKSFDCYDFYDRAKVGEKCTQDDWDLMKIPMKAMELKQKYGLDFKGEFVPTDRDMMEKLFQAGFEML  LECGIYCTDTHRIVKYTEDEIWDAINNVQKEFTLGTGRDAVNVRKRSVGDKRKPIVQGGPTGSPISEDVF  MPVHMSYALEKEVDTIVDGVMTSVRGKPPVPKSPYEVLAAKTEARLIKQACAMAGRPGMGIUGPETSLSA  QGNISSDCVGGQISSDSHEVSQLNELKIDLDAIAVIAHYKGNSDIIMDEQMPIFGGYAGGIEETTIVDVA  THINAFVMSSASWHLDGPVHIRWGSTNTRETLTIAGWACATISEFTDILSGNQYYPCAGPCTEMCLLEAS  AQSITDTASGREILSGVASAKGVVTDKTTGMEARMMGEVARATAGVEISEINKILDKLVALYEKNYANAP  AGKTFQECYDVKTVTPTEEYMHVYDGARKKLEDLGLVF |
|  |  | Predicted ORF dimethylamine methyltransferase [Methanosarcina mazei Goe1] | >ORF 6431 and organism AE010299.seq  MATEYALRMGDGKRIFLTKEKIREELEAGAANAADLGEIPALSGDEIDRLAEILMMPGKAVSVEQGMEVP  VTHDIGTIRLDGDQGNSGVGIPSSRLVGCMMHERAFGADTMELGHIDYSFKPVKPVVSNECQAMEVCQQN  MIIPLFYGAMPNMGLYYTPDGPFENPGDLMKAFKIQEAWESMEHAADHLTRDTVWIMQKLFASGADGVNF  DTTAAAGDGDFYGTLHAVEALRKEFPAMHIEVGMAGEMVLGMHGNLQYEGVTLAGLWPHQQASLVAKAGA  NVFGPVVNTNTSKTSPWNLARAVTFIKEAVKVSSLPCHVDMGMGVGGIPMLETPPIDAVTRASKAMVEVA  GVDGIUIGVGDPLGMPISHIMASGMTGIRAAGDLVARMQFSKNMRIKEAKKYVAKKLDVDVMDLADEHVM  RELREELDIGVITSVPGAAKGIAAKMNIEKLLDVRINSCELFRKQTGR |
|  |  | Predicted ORF dimethylamine methyltransferase [Methanosarcina mazei Goe1] | >ORF 125 and organism AE010299.seq  MATEYALRMGDGKRVFLTKEKILEELEAGMANASDLGEIPDLSADEIDKLAEILMMPGKAVSVEQGMEVP  VTHDIGTIRLDGDQGNSGVGIPSSRLVGCMTHERAFGADTMELGHIDYSFKPVKPVVSNECQAMEVCQQN  MIIPLFYGAMPNMGLYYTPDGPFENPGDLMKAFKIQEAWESMEHAAEHLTRDTVWVMQKLFASGTDGVNF  DTTAAAGDADMYGTLHAIEALRKEFPDMYIEAGMAGECVLGMHGNLQYDGVTLAGLWPHQQAPLVAKAGA  NVFGPVCNTNTSKTSAWNLARAVNFMKAAVQASPIPCHVDMGMGVGGIPMLETPPVDAVTRASKAMVEVA  GVDGIUIGVGDPLGMPISHIMASGMTGIRAAGDLVARMQFSKNMRIGEAKEYVAKKLNVDVMDLADEHVM  RELREELDIGVITSVPGAAKGIAAKMNIEKLLDIKINSCNLFRKQIQ |
|  |  | Predicted ORF dimethylamine methyltransferase [Methanosarcina mazei Goe1] | >ORF 566 and organism AE010299.seq  MATEYALRMGDGKRIYLTKEKILSEIEAGSSNAADLGDIPDLSTDEMKKLAEILMMPGKAVSVEQGMEVP  VTHDIGTIRLDGDQGNSGVGIPSSRLVGCMTHERAFGADTMELGHIDYSFKPVKPVVSNECQAMEVCQQN  MIIPLFYGAMPNMGLYYTPDGPFENPGDLMKMFKIDKARESMEHAADHLTRDTVWVMQKLFASGADGVNF  DTTAAAGDADMYGTLRAVEVLRAQFPEMYIEVGMAGEMVLGMHGELEYDEVRLAGLWPHEQAPLIAKAGA  NVFGPVVNTNTSKTSAWNLARAVTFIKEAVKASPIPCHVNMGMGVGGIPMLETPPVDAVTRASKAMVEVA  GVDGIUIGVGDPLGMPISHIMASGMTGIRAAGDLVARMEFSKNMRIGEAKEYVAKKLNVDTMDLADEHVM  RELREELDIGVITSVPGAAKGIAAKMNIEKLLGIKINSCETFRAQLA |
| DRFAT | *M barkeri* | >gi|19914320|gb|AAM03976.1| dimethylamine methyltransferase [Methanosarcina acetivorans str. C2A] | >ORF 194mbark.fasta 1283 and organism 194mbark.fasta  MATEYALRMGDGKRVFLTKEKIMAEIEAGTANAADLGDIPALNDNEMDKLAEILMMPGKTVSVEQGMEIP  VTHDIGTIRLDGDQGNSGVGIPSSRLVGCMMHERAFGADTMELGHIDYSFKPVKPVVSNECQAMEVCQQN  MIIPLFYGAMPNMGLYYTPDGPFENPGDLMKLFKIDKAKESMEHAAEHLTRDTVWVMQKLFASGADGVNF  DTTGAAGDGDMYGTLYAIQALRKEFPDMYIEAGMAGEMVLGMHGELEYDGVRLAGSWPHEQAPLIAKAGA  NVFGPVCNTNTSKTSAWNLARAVTFIKAAVEASPIPCHVNMGMGVGGIPMLETPPIDAVTRASKAMVEIA  GVDGI**U**IGVGDPMGMPISHIMASGMTGIRAAGDLVARMEFSKNMRIGEAKEYVAKKLGVDKMDLVDEHVM  RELREELDIGIITSVPGAAKGIAAKMNIEKLLDIKINSCNLFRKQIA |
|  |  | >gi|19913899|gb|AAM03597.1| monomethylamine methyltransferase [Methanosarcina acetivorans str. C2A] | >ORF 194mbark.fasta 1314 and organism 194mbark.fasta  MTFRKSFDCYDFYDRAKVGEKCTLDDWDLMRIPMKAMELKQKYGLDFKGEFIPTDKDMMEKLFKAGFEML  LECGIYCTDTHRIVKYTEDEIWDAINNAQKEFVLGTGRDAVNVRKRSVGDKAKPIVQGGPTGSPISEDVF  MPVHMSYALEKEVDTIVNGVMTTVRGKAPVPKSPYEVLAAKTETRLIKNACAMAGRPGMGV**U**GPETSLSA  QGNISADCAGGMTCTDSHEVSQLCELKIDLDAISVIAHYNGNSDIIMDEQMPIFGGYAGGIEETTIVNIA  THINSLVMSNASWHLDGPVHIRWGSTNTRETLTIAGWACATISEFTDILSGNQYYPCAGPCTEMCLLEAS  AQSITDTASGREILSGVASAKGVVTDKTTGMEARMMGEVARATAGVEISEVNVILDKLVALYEKNYASAP  AGKTFQECYDVKTVTPTEEYMQVYDGARKKLEDLGLVF |
|  |  | >gi|19914316|gb|AAM03972.1| trimethylamine methyltransferase [Methanosarcina acetivorans str. C2A] | >ORF 208mbark.fasta 3202 and organism 208mbark.fasta  MAKNNAVAGFNALNGVELNLFTTDELKAIHYATMEVLMDPGIQVSDPEARQIFKENGCEVDEKTNVVKIP  EYLVRKALQLAPSRFILWGRDKKFNTVQECGGKVHWTCFGTGVKMCKYQDGKYVTVDSVEKDIADIAKLC  DWAENIDYFSLPVSARDIAGQGAQDVHETLTPIANTAKHFHHIDPVGENVEYYRDIVKAYYGGDEEEARK  KPIFSMLLCPTSPLELSVNACQVIIKGARYGIPVNVLSMAMSGGSSPVYLAGTLVTHNAEVLSGIVLAQL  TVPGAKVWYGSSTTTFDLKKGTAPVGSPELGLISAAVAKLAQFYGLPSYVAGS**U**SDAKVPDDQAGHEKTM  TTLLPALSGANTIYGAGMLELGMTFSMEQLVIDNDIFSMVKKAMKGIPVSEETLAVESIQKVGIGNNFLA  LKQTRQLVDYPSNPMLLDRHMFGDWAAAGSKDLATVAHEKVEDVLKNHQVTPIDADILKDMQAIVDKADK  AFRGM |
|  |  | >gi|19914320|gb|AAM03976.1| dimethylamine methyltransferase [Methanosarcina acetivorans str. C2A] | >ORF 208mbark.fasta 3262 and organism 208mbark.fasta  MATEYALRMGDGKRIYLTKEKIIAEIEDGTANAADLGEIPALNANEMEKLAEILMMPGKTVSVEQGMEVP  VTHDIGTIRLDGDQGNSGVGIPSSRLVGCMTHERAFGADTMELGHIDYSFKPVKPVVSNECQAMEVCQQN  MIIPLFYGAMPNMGLYYTPDGPFENPGDLMKAFKIPEAWESMEHAAEHLTRDTVWVMQKLFASGADGVNF  DTTGAAGDGDMYGTLHAIEALRKEFPDMYIEAGMAGECVLGMHGNLQYDGVTLAGLWPHQQAPLVAKAGA  NVFGPVCNTNTSKTSAWNLARAVTFMKAAVEASPIPCHVDMGMGVGGIPMLETPPIDAVTRASKAMVEIA  GVDGI**U**IGVGDPMGMPIAHIMASGMTGMRAAGDLVARMEFSKNMRIGEAKEYVAKKLGVDQMDLVDEHVM  RELREELDIGIITSVPGAAKGIAAKMNIEKLLDIKINSCNLFRKQIA |
|  |  | >gi|19914334|gb|AAM03988.1| protein-L-isoaspartate (D-aspartate) O-methyltransferase [Methanosarcina acetivorans str. C2A] | >ORF 208mbark.fasta 3412 and organism 208mbark.fasta  LRERNGKVRVSVNSEEGRGKEKDNREKEEEKLEAMRRYLVDNLEAYLLLKDNVREAMLRVPRHKFVPEYE  QKAAYMDRPLDIGHGQTISAPHMVAMMCELLELSEGHKVLEIGTGSGYNAAVMGELVGKSGHVYTVERIE  VLANFARENLKKAGYNNVTVLLEDGSMGYS**U**YAPYDRIAVTCAAPYIPEPLLEQLKPGGIMLIPVGDYFQ  ELYKIKKDSKGRIHKKKKGEVIFVPLIGKHGFRKSLEC |
|  |  | >gi|19913899|gb|AAM03597.1| monomethylamine methyltransferase [Methanosarcina acetivorans str. C2A] | >ORF 209mbark.fasta 3584 and organism 209mbark.fasta  MTFRKSFDCYDFYDRAKVGEKCTQDDWDLMKIPMKAMELKQKYGLDFKGEFIPTDKDMMEKLFKAGFEML  LECGIYCTDTHRIVKYTEDEIWDAINNVQKEFVLGTGRDAVNVKKRSVGDKAKPIVQGGPTGSPISEDVF  MPVHMSYALEKEVDTIVNGVMTSVRGKAPVPKSPYEVLAAKTETRLIKNACAMAGRPGMGV**U**GPETSLSA  QGNISADCAGGMTCTDSHEVSQLNELKIDLDAISVIAHYKGNSDIIMDEQMPIFGGYAGGIEETTIVDVA  THINAVIMSSASWHLDGPVHIRWGSTNTRETLTIAGWACATISEFTDILSGNQYYPCAGPCTEMCLLEAS  AQSITDTASGREILSGVASAKGVVTDKTTGMEARMMGEVARATAGVEISEVNVILDKLVALYEKNYASAP  AGKTFQECYDVKTVTPTEEYMQVYDGARKKLEDLGLVF |
|  |  | >gi|19913899|gb|AAM03597.1| monomethylamine methyltransferase [Methanosarcina acetivorans str. C2A] | >ORF 209mbark.fasta 3912 and organism 209mbark.fasta  MTFRKSFDCYDFYDRAKVGEKCTQDDWDLMKIPMKAMELKQKYGLDFKGEFIPTDKDMMEKLFKAGFEML  LECGIYCTDTHRIVKYTEDEIWDAINNVQKEFVLGTGRDAVNVKKRSVGDKAKPIVQGGPTGSPISEDVF  MPVHMSYALEKEVDTIVNGVMTSVRGKAPVPKSPYEVLAAKTETRLIKNACAMAGRPGMGV**U**GPETSLSA  QGNISADCAGGMTCTDSHEVSQLNELKIDLDAISVIAHYKGNSDIIMDEQMPIFGGYAGGIEETTIVDVA  THINAVIMSSASWHLDGPVHIRWGSTNTRETLTIAGWACATISEFTDILSGNQYYPCAGPCTEMCLLEAS  AQSITDTASGREILSGVASAKGVVTDKTTGMEARMMGEVARATAGVEISEVNVILDKLVALYEKNYASAP  AGKTFQECYDVKTVTPTEEYMQVYDGARKKLEDLGLVF |
| DRFAT | *M. burtonii* | >gi|19914320|gb|AAM03976.1| dimethylamine methyltransferase [Methanosarcina acetivorans str. C2A] | >ORF 14mburtonii.fasta 312 and organism 14mburtonii.fasta  MTWIMQKLQNVGSDGVNFDTIGAAGDGDMYASLNSIEALRKEFPGMYIEAGMAGELVLGMHGELEYDGTV  LAGLWPHQQAALIAKAGANIFGPVCNTNTSRTSAWNLGRAVTFTKAAVEASTIPVHVNMGMGVGGSPMLE  TPPIDAVTRASKAMVEIAGVDGI**U**IGVGDPMGMPISHIMASGMTGIRAAGDLVARMQFDKSMRIGEAKDF  VAKKLGVSNADLSDEYVMRELREELDIGVITSVPGCAKGIAAKMNIEKLLGIDINCCDRFREITG |
|  |  | >gi|19914753|gb|AAM04365.1| trimethylamine methyltransferase [Methanosarcina acetivorans str. C2A] | >ORF 14mburtonii.fasta 319 and organism 14mburtonii.fasta  MDISKRRLNMTNENLCAGRNRYDGVSIDFFSDADLRAIDSATMDVFQNPGIQVSDAESRALFKEAGCDVN  EKTMVVKIPEHVVRRAIMTAPSKITLYGREKQHTFTQQAGGKVHYTCFGTGVKMCKYEAPGVFKTVDSTE  EDLANTARVCDWADNIDSYSLAVSARDWAGKGAQDVHETFTPLMNTSKHFFHIDPVGENVEYYWDILKAY  YGGDEEQARSRPIFSELLCPTSPLEIGTNACQLILKSVNFGIPINVISMAMSGASCPVHLAGTLVTHNAE  VLSGIVLAQLASPGAKVWYGSSTTAFDLKHGTAPVGSPELGLISAAVAKLGQYYDLPTYVAST**U**TDAKVP  DGQAGHEKTLTNLLPALAGANTLYGAGMLELGMTFSMEQLMIDNDIISMGKKVMKGIPVNDETLGLASIQ  KVGIGNNFLAHKETRDNINLVSSPDIFDRDMFGDWAAAGSKDIATVAHEKVTEILKNHEVTPIDSDLVRD  MKAVVDRADADFRSSM |
|  |  | >gi|18893258|gb|AAL81299.1| hypothetical protein [Pyrococcus furiosus DSM 3638  >gi|5458504|emb|CAB49992.1| Methlytransferase, putative [Pyrococcus abyssi]  >gi|57158963|dbj|BAD84893.1| SAM-dependent methyltransferase, UbiE/COQ5 family [Thermococcus kodakaraensis KOD1] | >ORF 15mburtonii.fasta 341 and organism 15mburtonii.fasta  MKRSAFNIHTWKYDLWYNKNSAVYASELEAIRELMPSKIAHNSIEIGVGTGRFASELGITYGLDPSARML  KIAESRKVECIKGVGESLPFKGSSMKLALIVTSLCFMDAKKVL**U**EAYRMLAPEGYLIVAFVERNSLLGEE  YRKEASESSFFKNIEFHTREEVLLMLKEHGFEDMHIRQTLFKPLTKI |
|  |  | >gi|18893258|gb|AAL81299.1| hypothetical protein [Pyrococcus furiosus DSM 3638]  >gi|5458504|emb|CAB49992.1| Methlytransferase, putative [Pyrococcus abyssi] | >ORF 15mburtonii.fasta 342 and organism 15mburtonii.fasta  MLAPEGYLIVAFVERNSLLGEEYRKEASESSFFKNIEFHTREEVLLMLKEHGFEDMHIRQTLFKPLTKI**U**  DLEKPEKDFGKGSFVTIRARSIKYK |
|  |  | >gi|19914320|gb|AAM03976.1| dimethylamine methyltransferase [Methanosarcina acetivorans str. C2A] | >ORF 19mburtonii.fasta 503 and organism 19mburtonii.fasta  VYNMATEYFLRMGDGQKIFMTKEDIRADIEAGSADAADLGDIPALSENEMDHMLDIITSPGRIVGVEPGM  EVPVTHDIGAIRIDGDQGNSGVGIPASRLVGSMIHERAFGADTMELGHIDYSYKPVKPVISQECQTMESC  QQNMIIPMLYGAMPNMGLYYTPDGPFENPGDLMKAFKISEAQDSIFHAAEHGIRDMTWIMQKLQNVGCDG  VNFDTIGAAGDGDMYASLNAIEALRKEFPGIYIEAGMAGELVLGMHGELEYDGTVLAGLWPHQQAPLVAK  AGANIFGPVCNTNTSRTSAWNLGRSVTFTKAAVEASTIPCHADMGMGVGGIPMLETPPIDAVTRASKAMV  EIAGVDGI**U**IGVGDPMGMPISHIMASGMSGMRAAGDLVARMQFDKSMRIGEAKDFVAKKLGVSNADLSDE  YVMRELREELDIGVITSVPGCAKGIAAKMNIEKLLGIDINCCDKFRETIA |
|  |  | >gi|19914753|gb|AAM04365.1| trimethylamine methyltransferase [Methanosarcina acetivorans str. C2A] | >ORF 2mburtonii.fasta 1223 and organism 2mburtonii.fasta  MSEPNYAARFPLQGVQLELFSEDDLRAIHYASMEVFLNPGVQVSDPEARAIFKEGGCEVDEASQIVKIPE  YVVNRALFDAPSRFMLYGRDKKNTMEQEHKGKVHYIPFGTGVKMCNYVAPGKYQTVDSVEQDIADTAKVC  DYLDEFSYMALTVSARDWAGKGAQDVHETLTPLMNTTKHFHHIDPVGENVEYYKGIVDAYYGGDSEAARK  RPIMSMLVCPTSPLELSVNACQVIMKGARFGMPVNVLSMAMSGGSSPVFRAGTLVTHNAEVLAGIVLAQL  VQPGAEVWYGSSTTTFDLRKGTAPVGAPELGIISAAVGKMGQFYGLPTYVAGT**U**SDSKVPDTQSGHEKTM  TTLLPAFAGCNTIYGGGMLELGMTFSLDQFVIDADIINMTRSAMRGVPVSDETLAVPSIQKVGIGNNFLA  HKETRENIHLVSNPELIDRDMFGDWEAAGSKDLSVVAHEKLLDIMKNHEVKAIDSELLADMKAVVDKADA  AFRASL |
|  |  | >gi|19913899|gb|AAM03597.1| monomethylamine methyltransferase [Methanosarcina acetivorans str. C2A] | >ORF 4mburtonii.fasta 1745 and organism 4mburtonii.fasta  LGELKMTFTKSVTCFDFYDRAQKGEKCTQDDWDLMTIPMKSMELKQKYNLDFGTESVPTDKDQMERLFKA  GFEMLLECGIYCTDTKRIVKYTEDEIWDAINNPMPAFQLGTGRDSVQMKKRTVGDKRKPIVQGGPTGSPI  SEDMFMPIHMSYALEKEVDTIVNGVMMTIRGKPPIPGSPYEILAAKSETRLIRNAAAMAGRPGMAV**U**GPE  TSLSAQGNIASDCVGGQVTSDSHEVSQLNELKIDLDAIAVIAHYKGNSDIIMDEQMPIFGGYAGGIEETT  IVDIATSLNSMVMSSASWHLDGPVHIRWGSTNTRETLQIAGWACATLSEFTDLMTGNQYYPCAGPCTEMC  LLEAAAQSVNDTASGREILSGVAAAKGVITDKTTGMEARMMGEVSRATTGMDIDSVNAVINNIVSSYEGN  YANAPEGKRFQDCYDVATITPTDEYVKVYEGAIKKLEDFGLTF |

 

 

**Master list:� PYRROLYSINE-containing proteins in *Methanosarcina
sp.* (These predictions are lower confidence than the selenoprotein
predictions)**

 

|  |  |  |  |
| --- | --- | --- | --- |
| **Accession ID** | ***Organism*** | Functional  Assignment | FASTA |
| AE008384 | *Methanosarcina mazei* | AE008384.1026.seq:>gi|19915283|gb|AAM04840.1| transposase [Methanosarcina acetivorans str. C2A]  AE008384.1026.seq:>gi|26109888|gb|AAN82093.1|AE016766\_181 Unknown protein encoded by ISEc8 within prophage [Escherichia coli CFT073] | >ORF 1026 and organism AE008384.seq  MKRDEILSYCASNPEIIVAYIESLESQVKELTERLVALESRLNQNSRNSSRPPSTDYFVKEKPNPKSLRK  PSGKKPGGQEGHPGTTLDMVDHPEUVIEHSLTCCKECGSTLENVEVEAYERRQVFDIPPVNLIVTEHKSQ  IKTCPCCGKLNKAVFPESVKYPVQYGPNILASAIYCKNYQFVPYDRISELFEDIMGIKICPATIIRAERE  CFQNLEEFENVIREKLLASPVINFDETGMKIEGKRHWLHVASNEKYTCYFAHTKRGAEAIDAMGILPKFK  GVAVHDGWKPYNVYDCDHALCNAHLQRELTGIEENYKQTWAKEMNELLTEMKKYTDECKEQLREPDFEQI  KALEERFDAIIIRALEENPHSLNPEKQGKRGKNPKTKSRNLLDRFIEHKEKILRFLTDLKVPFDNNQAER  DIRMMKLQQKISGTFRKAMGAQAFCRIRAYISTGKKNGLPVLEGIRAALIGAPLTIL |
|  |  | gi|19915283|gb|AAM04840.1| transposase [Methanosarcina acetivorans str. C2A] | >ORF 150 and organism AE008384.seq  MLTREEILIIYDAGPEAVISVIQRLETIIEEQSIRIAELEERVKVLESRLNQNSRNSSRPPSTDFFIKEK  PNPKSLRKKSGKKPGGQDGHPGTTLEMVDHPEUVIEHSLSCCKECGHTLENVEVEAYEKRQVFDIPPVNL  IVTEHKSQIKTCPYCGKINKAVFPESVKYPVQYGPNILASAIYCKNHHFIPYERISEFFEDIMGIKICPA  TIIRAERECFQNLEEFENVIREKLLASPVINFDETGMKIEGKRHWLHVDSNEKYTCYLPHSKRGAEAIDA  MGILPEFKGVAVHDGWKPYNVYDCDHALCNAHLQRELTGIEENYKQQWAKEMNELLTEMKKYTDECKDQV  KELDFEQIKALEERFDAIIIKGIEENPQSLNPEKKGKRGKNPKTKARNLLDRFIEHKENILRFLTDLKVP  FENNQAERDIRMMKLQQKISGTFRTIQGAEAFCRIRAYISTIRKNGLPVLEGIIAALKRAPLTIP |
|  |  | AE008384.1142.seq:>gi|19915283|gb|AAM04840.1| transposase [Methanosarcina acetivorans str. C2A]  AE008384.1142.seq:>gi|26109888|gb|AAN82093.1|AE016766\_181 Unknown protein encoded by ISEc8 within prophage [Escherichia coli CFT073] | >ORF 1142 and organism AE008384.seq  MKRDEILSYCASNPEIIVAYIESLESQVKELTERLVALESRLNQNSRNSSRPPSTDYFVKEKPNPKSLRK  PSGKKPGGQEGHPGTTLDMVDHPEUVIEHSLTCCKECGSTLENVEVEAYERRQVFDIPPVNLIVTEHKSQ  IKTCPCCGKLNKAVFPESVKYPVQYGPNILASAIYCKNYQFVPYDRISELFEDIMGIKICPATIIRAERE  CFQNLEEFENVIREKLLASPVINFDETGMKIEGKRHWLHVASNEKYTCYFAHTKRGAEAIDAMGILPKFK  GVAVHDGWKPYNVYDCDHALCNAHLQRELTGIEENYKQTWAKEMNELLTEMKKYTDECKEQLREPDFEQI  KALEERFDAIIIRALEENPHSLNPEKQGKRGKNPKTKSRNLLDRFIEHKEKILRFLTDLKVPFDNNQAER  DIRMMKLQQKISGTFRKAMGAQAFCRIRAYISTGKKNGLPVLEGIRAALIGAPLTIL |
|  |  | AE008384.1582.seq:>gi|19915283|gb|AAM04840.1| transposase [Methanosarcina acetivorans str. C2A]  AE008384.1582.seq:>gi|26109888|gb|AAN82093.1|AE016766\_181 Unknown protein encoded by ISEc8 within prophage [Escherichia coli CFT073] | >ORF 1582 and organism AE008384.seq  MKRDEILSYCASNPEIIVAYIESLESQVKELTERLVALESRLNQNSRNSSRPPSTDYFVKEKPNPKSLRK  PSGKKPGGQEGHPGTTLDMVDHPEUVIEHSLTCCKECGSTLENVEVEAYERRQVFDIPPVNLIVTEHKSQ  IKTCPCCGKLNKAVFPESVKYPVQYGPNILASAIYCKNYQFVPYDRISELFEDIMGIKICPATIIRAERE  CFQNLEEFENVIREKLLASPVINFDETGMKIEGKRHWLHVASNEKYTCYFAHTKRGAEAIDAMGILPKFK  GVAVHDGWKPYNVYDCDHALCNAHLQRELTGIEENYKQTWAKEMNELLTEMKKYTDECKEQLREPDFEQI  KALEERFDAIIIRALEENPHSLNPEKQGKRGKNPKTKSRNLLDRFIEHKEKILRFLTDLKVPFDNNQAER  DIRMMKLQQKISGTFRKAMGAQAFCRIRAYISTGKKNGLPVLEGIRAALIGAPLTIL |
|  |  | AE008384.1684.seq:>gi|19915283|gb|AAM04840.1| transposase [Methanosarcina acetivorans str. C2A]  AE008384.1684.seq:>gi|26109888|gb|AAN82093.1|AE016766\_181 Unknown protein encoded by ISEc8 within prophage [Escherichia coli CFT073] | >ORF 1684 and organism AE008384.seq  MLTREEILIIYDAGPEAVISVIQRLETIIEEQSIRIAELEERVKVLESRLNQNSRNSSRPPSTDFFIKEK  PNPKSLRKKSGKKPGGQDGHPGTTLEMVDHPEUVIEHSLSCCKECGHTLENVEVEAYEKRQVFDIPPVNL  IVTEHKSQIKTCPYCGKINKAVFPESVKYPVQYGPNILASAIYCKNHHFIPYERISEFFEDIMGIKICPA  TIIRAERECFQNLEEFENVIREKLLASPVINFDETGMKIEGKRHWLHVASNEKYTCYLPHSKRGAEAIDA  MGILPEFKGVAVHDGWKPYNVYDCDHALCNAHLQRELTGIEENYKQQWAKEMNELLTEMKKYTDECKDQV  KELDFEQIKALEERFDAIIIKGIEENPQSLNPEKKGKRGKNPKTKARNLLDRFIEHKENILRFLTDLKVP  FENNQAERDIRMMKLQQKISGTFRTIQGAEAFCRIRAYISTIRKNGLPVLEGIIAALKRAPLTIP |
|  |  | AE008384.1877.seq:>gi|19915283|gb|AAM04840.1| transposase [Methanosarcina acetivorans str. C2A]  AE008384.1877.seq:>gi|26109888|gb|AAN82093.1|AE016766\_181 Unknown protein encoded by ISEc8 within prophage [Escherichia coli CFT073] | >ORF 1877 and organism AE008384.seq  MKRDEILSYCASNPEIIVAYIESLESQVKELTERLVALESRLNQNSRNSSRPPSTDYFVKEKPNPKSLRK  PSGKKPGGQEGHPGTTLDMVDHPEUVIEHSLTCCKECGSTLENVEVEAYERRQVFDIPPVNLIVTEHKSQ  IKTCPCCGKLNKAVFPESVKYPVQYGPNILASAIYCKNYQFVPYDRISELFEDIMGIKICPATIIRAERE  CFQNLEEFENVIREKLLASPVINFDETGMKIEGKRHWLHVASNEKYTCYFAHTKRGAEAIDAMGILPKFK  GVAVHDGWKPYNVYDCDHALCNAHLQRELTGIEENYKQTWAKEMNELLTEMKKYTDECKEQLREPDFEQI  KALEERFDAIIIRALEENPHSLNPEKQGKRGKNPKTKSRNLLDRFIEHKEKILRFLTDLKVPFDNNQAER  DIRMMKLQQKISGTFRKAMGAQAFCRIRAYISTGKKNGLPVLEGIRAALIGAPLTIL |
|  |  | AE008384.2042.seq:>gi|19915283|gb|AAM04840.1| transposase [Methanosarcina acetivorans str. C2A]  AE008384.2042.seq:>gi|26109888|gb|AAN82093.1|AE016766\_181 Unknown protein encoded by ISEc8 within prophage [Escherichia coli CFT073] | >ORF 2042 and organism AE008384.seq  MKRDEILSYCASNPEIIVAYIESLESQVKELTERLVALESRLNQNSRNSSRPPSTDYFVKEKPNPKSLRK  PSGKKPGGQEGHPGTTLDMVDHPEUVIEHSLTCCKECGSTLENVEVEAYERRQVFDIPPVNLIVTEHKSQ  IKTCPCCGKLNKAVFPESVKYPVQYGPNILASAIYCKNYQFVPYDRISELFEDIMGIKICPATIIRAERE  CFQNLEEFENVIREKLLASPVINFDETGMKIEGKRHWLHVASNEKYTCYFAHTKRGAEAIDAMGILPKFK  GVAVHDGWKPYNVYDCDHALCNAHLQRELTGIEENYKQTWAKEMNELLTEMKKYTDECKEQLREPDFEQI  KALEERFDAIIIRALEENPHSLNPEKQGKRGKNPKTKSRNLLDRFIEHKEKILRFLTDLKVPFDNNQAER  DIRMMKLQQKISGTFRKAMGAQAFCRIRAYISTGKKNGLPVLEGIRAALIGAPLTIL |
|  |  | AE008384.2493.seq:>gi|19915283|gb|AAM04840.1| transposase [Methanosarcina acetivorans str. C2A] | >ORF 2493 and organism AE008384.seq  MLTREEILIIYDAGPEAVISVIQRLETIIEEQSIRIAELEERVKVLESRLNQNSRNSSRPPSTDFFIKEK  PNPKSLRKKSGKKPGGQDGHPGTTLEMVDHPEUVIEHSLSCCKECGHTLENVEVEAYFYLRC |
|  |  | AE008384.2612.seq:>gi|19915283|gb|AAM04840.1| transposase [Methanosarcina acetivorans str. C2A]  AE008384.2612.seq:>gi|26109888|gb|AAN82093.1|AE016766\_181 Unknown protein encoded by ISEc8 within prophage [Escherichia coli CFT073] | >ORF 2612 and organism AE008384.seq  MLTREEILALCASNPEVIAYIVSLETQIKELTERLIALESRLNQNSRNSSRPPSTDFFVKEKPNPKSLRK  KSGKKPGGQDGHPGTTLEMVDDPEUVIEHSLSCCKECGHTLENVEVEAYEKRQVFDIPPVNLIVTEHKSQ  IKTCPHCGRINKAVFPESVKYPVQYGPNILASAIYCKNHHFIPYERISEFFEDIMGIKICPATIIRAEKE  CFQNLECFENIIREKLMTSYVVHFDETGMKIEGKRHWLHVASNDKYTCYLPHSKRGAEAIDAMGILPEFK  GVAVHDGWKPYNVYDCDHALCNAHLQRELTGIEENYKQQWAKEMNKLLTEMKKYTDECKEQVKELDFEQI  KALEERFDAIIMKGIEENPQSLNPEKQGKRGKNPKTKARNLLDRFIEHKEKILRFLKDLKVPFENNQAER  DIRMMKLQQKISGTFRTTQGAQAFCRMRAYISTIRKNGLLVLEGIIAALKGAPLTIT |
|  |  | AE008384.2740.seq:>gi|19915283|gb|AAM04840.1| transposase [Methanosarcina acetivorans str. C2A]  AE008384.2740.seq:>gi|26109888|gb|AAN82093.1|AE016766\_181 Unknown protein encoded by ISEc8 within prophage [Escherichia coli CFT073] | >ORF 2740 and organism AE008384.seq  MKRDEILSYCASNPEIIVAYIESLESQVKELTERLVALESRLNQNSRNSSRPPSTDYFVKEKPNPKSLRK  PSGKKPGGQEGHPGTTLDMVDHPEUVIEHSLTCCKECGSTLENVEVEAYERRQVFDIPPVNLIVTEHKSQ  IKTCPCCGKLNKAVFPESVKYPVQYGPNILASAIYCKNYQFVPYDRISELFEDIMGIKICPATIIRAERE  CFQNLEEFENVIREKLLASPVINFDETGMKIEGKRHWLHVASNEKYTCYFAHTKRGAEAIDAMGILPKFK  GVAVHDGWKPYNVYDCDHALCNAHLQRELTGIEENYKQTWAKEMNELLTEMKKYTDECKEQLREPDFEQI  KALEERFDAIIIRALEENPHSLNPEKQGKRGKNPKTKSRNLLDRFIEHKEKILRFLTDLKVPFDNNQAER  DIRMMKLQQKISGTFRKAMGAQAFCRIRAYISTGKKNGLPVLEGIRAALIGAPLTIL |
|  |  | AE008384.2830.seq:>gi|19915283|gb|AAM04840.1| transposase [Methanosarcina acetivorans str. C2A]  AE008384.2830.seq:>gi|26109888|gb|AAN82093.1|AE016766\_181 Unknown protein encoded by ISEc8 within prophage [Escherichia coli CFT073] | >ORF 2830 and organism AE008384.seq  MKRDEILSYCASNPEIIVAYIESLESQVKELTERLVALESRLNQNSRNSSRPPSTDYFVKEKPNPKSLRK  PSGKKPGGQEGHPGTTLDMVDHPEUVIEHSLTCCKECGSTLENVEVEAYERRQVFDIPPVNLIVTEHKSQ  IKTCPCCGKLNKAVFPESVKYPVQYGPNILASAIYCKNYQFVPYDRISELFEDIMGIKICPATIIRAERE  CFQNLEEFENVIREKLLASPVINFDETGMKIEGKRHWLHVASNEKYTCYFAHTKRGAEAIDAMGILPKFK  GVAVHDGWKPYNVYDCDHALCNAHLQRELTGIEENYKQTWAKEMNELLTEMKKYTDECKEQLREPDFEQI  KALEERFDAIIIRALEENPHSLNPEKQGKRGKNPKTKSRNLLDRFIEHKEKILRFLTDLKVPFDNNQAER  DIRMMKLQQKISGTFRKAMGAQAFCRIRAYISTGKKNGLPVLEGIRAALIGAPLTIL |
|  |  | AE008384.2844.seq:>gi|19915283|gb|AAM04840.1| transposase [Methanosarcina acetivorans str. C2A]  AE008384.2844.seq:>gi|26109888|gb|AAN82093.1|AE016766\_181 Unknown protein encoded by ISEc8 within prophage [Escherichia coli CFT073] | >ORF 2844 and organism AE008384.seq  MLTREEILALCASNPEVIAYIVSLETQIKELTERLIALESRLNQNSRNSSRPPSTDFFVKEKPNPKSLRK  KSGKKPGGQDGHPGTTLEMVDDPEUVIEHSLSCCKECGHTLENVEVEAYEKRQVFDIPPVNLIVTEHKSQ  IKTCPHCGRINKAVFPESVKYPVQYGPNILASAIYCKNHHFIPYERISEFFEDIMGIKICPATIIRAEKE  CFQNLECFENIIREKLMTSYVVHFDETGMKIEGKRHWLHVASNDKYTCYLPHSKRGAEAIDAMGILPEFK  GVAVHDGWKPYNVYDCDHALCNAHLQRELTGIEENYKQQWAKEMNKLLTEMKKYTDECKEQVKELDFEQI  KALEERFDAIIMKGIEENPQSLNPEKQGKRGKNPKTKARNLLDRFIEHKEKILRFLKDLKVPFENNQAER  DIRMMKLQQKISGTFRTTQGAQAFCRMRAYISTIRKNGLLVLEGIIAALKGAPLTIT |
|  |  | AE008384.3082.seq:>gi|15622302|dbj|BAB66294.1| 106aa long conserved hypothetical protein [Sulfolobus tokodaii str. 7]  AE008384.3082.seq:>gi|5104578|dbj|BAA79893.1| 122aa long hypothetical protein [Aeropyrum pernix K1] | >ORF 3082 and organism AE008384.seq  LVMILPPVALESLSMSLFFTLRIAKMPAFVRUYWARSSIPFWQNTTFAPVSITLSTISLSIASSWSRKSC  IWSGVVIFISALVSVFLSSIGTLKRRTFASFTTSGMPVWTRSLSTITPSTISESLMLPPGLFSVLTSSMS  TVIFPFSSFSTMDLIARTARLER |
|  |  | AE008384.3160.seq:>gi|19915283|gb|AAM04840.1| transposase [Methanosarcina acetivorans str. C2A]  AE008384.3160.seq:>gi|26109888|gb|AAN82093.1|AE016766\_181 Unknown protein encoded by ISEc8 within prophage [Escherichia coli CFT073] | >ORF 3160 and organism AE008384.seq  MKRDEILSYCASNPEIIVAYIESLESQVKELTERLVALESRLNQNSRNSSRPPSTDYFVKEKPNPKSLRK  PSGKKPGGQEGHPGTTLDMVDHPEUVIEHSLTCCKECGSTLENVEVEAYERRQVFDIPPVNLIVTEHKSQ  IKTCPCCGKLNKAVFPESVKYPVQYGPNILASAIYCKNYQFVPYDRISELFEDIMGIKICPATIIRAERE  CFQNLEEFENVIREKLLASPVINFDETGMKIEGKRHWLHVASNEKYTCYFAHTKRGAEAIDAMGILPKFK  GVAVHDGWKPYNVYDCDHALCNAHLQRELTGIEENYKQTWAKEMNELLTEMKKYTDECKEQLREPDFEQI  KALEERFDAIIIRALEENPHSLNPEKQGKRGKNPKTKSRNLLDRFIEHKEKILRFLTDLKVPFDNNQAER  DIRMMKLQQKISGTFRKAMGAQAFCRIRAYISTGKKNGLPVLEGIRAALIGAPLTIL |
|  |  | AE008384.3242.seq:>gi|15156108|gb|AAK86886.1| AGR\_C\_1991p [Agrobacterium tumefaciens str. C58]  AE008384.3242.seq:>gi|17739471|gb|AAL42090.1| DNA repair protein [Agrobacterium tumefaciens str. C58]  AE008384.3242.seq:>gi|7226014|gb|AAF41195.1| DNA repair protein RadA [Neisseria meningitidis MC58] | >ORF 3242 and organism AE008384.seq  MVSSLPQSILUVSNMGEIEDKKNIISSGNDEIDKKLGEGIPLGSLVLIEGENDTGKSVFCQQMVYGGLNQ  LHRIAYYSTENTVKSMLAQMDSLSLDISDFYSWGYFRIFPVHLEGVEWTSEQMKGTLHLVTTHIKSVREK  VIIIDSLTMFTTYSDEDNILEFLTSLKNLCDKGYTIFITLHQHAFKEDTLVRIRSSCDCHLFLRKEQLTD  RYISVMEVSKIRGAKKSTGNIVSFEVQPGFGLKIIPISQAKV |
|  |  | gi|19915283|gb|AAM04840.1| transposase [Methanosarcina acetivorans str. C2A] | >ORF 310 and organism AE008384.seq  MKRDEILSYCASNPEIIVAYIESLESQVKELTERLVALESRLNQNSRNSSRPPSTDYFVKEKPNPKSLRK  PSGKKPGGQEGHPGTTLDMVDHPEUVIEHSLTCCKECGSTLENVEVEAYERRQVFDIPPVNLIVTEHKSQ  IKTCPCCGKLNKAVFPESVKYPVQYGPNILASAIYCKNYQFVPYDRISELFEDIMGIKICPATIIRAERE  CFQNLEEFENVIREKLLASPVINFDETGMKIEGKRHWLHVASNEKYTCYFAHTKRGAEAIDAMGILPKFK  GVAVHDGWKPYNVYDCDHALCNAHLQRELTGIEENYKQTWAKEMNELLTEMKKYTDECKEQLREPDFEQI  KALEERFDAIIIRALEENPHSLNPEKQGKRGKNPKTKSRNLLDRFIEHKEKILRFLTDLKVPFDNNQAER  DIRMMKLQQKISGTFRKAMGAQAFCRIRAYISTGKKNGLPVLEGIRAALIGAPLTIL |
|  |  | gi|19915283|gb|AAM04840.1| transposase [Methanosarcina acetivorans str. C2A] | >ORF 341 and organism AE008384.seq  MLTREEILALCASNPEVIAYIVSLETQIKELTERLIALESRLNQNSRNSSRPPSTDFFVKEKPNPKSLRK  KSGKKPGGQDGHPGTTLEMVDDPEUVIEHSLSCCKECGHTLENVEVEAYEKRQVFDIPPVNLIVTEHKSQ  IKTCPHCGRINKAVFPESVKYPVQYGPNILASAIYCKNHHFIPYERISEFFEDIMGIKICPATIIRAEKE  CFQNLECFENIIREKLMTSYVVHFDETGMKIEGKRHWLHVASNDKYTCYLPHSKRGAEAIDAMGILPEFK  GVAVHDGWKPYNVYDCDHALCNAHLQRELTGIEENYKQQWAKEMNKLLTEMKKYTDECKEQVKELDFEQI  KALEERFDAIIMKGIEENPQSLNPEKQGKRGKNPKTKARNLLDRFIEHKEKILRFLKDLKVPFENNQAER  DIRMMKLQQKISGTFRTTQGAQAFCRMRAYISTIRKNGLLVLEGIIAALKGAPLTIT |
|  |  | AE008384.3255.seq:>gi|19915283|gb|AAM04840.1| transposase [Methanosarcina acetivorans str. C2A]  AE008384.3255.seq:>gi|26109888|gb|AAN82093.1|AE016766\_181 Unknown protein encoded by ISEc8 within prophage [Escherichia coli CFT073] | >ORF 3255 and organism AE008384.seq  MLTREEILALCASNPEVIAYIVSLETQIKELTERLIALESRLNQNSRNSSRPPSTDFFIKEKPNPKSLRK  KSGKKPGGQDGHPGTTLEMVDHPEUVIEHSLSCCKECGHTLENVEVEAYEKRQVFDIPPVNLIVTEHKSQ  IKTCPYCGKINKAVFPESVKYPVQYGPNILASAIYCKNHHFIPYERISEFFEDIMGIKICPATIIRAERE  CFQNLEEFENVIREKLMISHVVHFDETGMKIEGKRHWLHVASNEKYTCYLPHSKRGAEAIDAMGILPEFK  GVAVHDGWKPYNVYDCDHALCNAHLQRELTGIEENYKQQWAKEMNELLTEMKKYTDECKDQVKELDFEQI  KALEERFDAIIIKGIEENPQSLNPEKKGKRGKNPKTKARNLLDRFIEHKENILRFLTDLKVPFENNQAER  DIRMMKLQQKISGTFRTIQGAEAFCRIRAYISTIRKNGLPVLEGIIAALKRAPLTIP |
|  |  | AE008384.3940.seq:>gi|33635662|emb|CAE21986.1| TPR repeat:HAT (Half-A-TPR) repeat [Prochlorococcus marinus str. MIT 9313]  AE008384.3940.seq:>gi|17132907|dbj|BAB75472.1| serine/threonine kinase [Nostoc sp. PCC 7120]  AE008384.3940.seq:>gi|33640682|emb|CAE20471.1| TPR repeat [Prochlorococcus marinus str. MIT 9313]  AE008384.3940.seq:>gi|2621106|gb|AAB84576.1| O-linked GlcNAc transferase [Methanothermobacter thermautotrophicus] | >ORF 3940 and organism AE008384.seq  LSMEMDAVDKLVFHVIEKVSEDECDLDKATESVISFSHENLLSPETLLKLSFIFGNDKMFREEYVVSRAS  ASLFSGKMREEAHMVAGKTASLLGLMESAAREFKEILEENPGNIEALCGYGSMLAGAGULDGARIQYEKA  LEFNPDHVETLCSYGCVLYRLSQLDKAEEVYRRALLLDPDHVESHCGYGILLNRRGQKTEAGLHYARALE  IDPEHVESNFRYARLLEEKGEPIEAETYYIVALKADPESSKLHLYYARLLAQHGLIHGARVHFRYALKIS  PEDVEAHCEYARLLARFGHRHEAEVQYKKALELNPGHFGSLSGYGDLLKEKGQYTEAEKIYRQAECFRQD  AW |
| AE010299 | *Methanosarcina acetivorans* | >gi|20907148|gb|AAM32242.1| Conserved hypothetical protein [Methanosarcina mazei Goe1]  >gi|22776199|dbj|BAC12476.1| transcriptional regulator (TetR/AcrR family) [Oceanobacillus iheyensis HTE831] | >ORF 1771 and organism AE010299.seq  VIEVSMKEIREQEKEQRRNYILDAAEKLFFSRGYDGVSMDDIANEVEFNKATLYLYFKNKESLFFTVVLR  GKRILNSMVEEGIKNCKTSIEVLDTIGKEYFUFIGEYPDYSRMYSYFHSGRFNLEDSEDMDEVAKEILKL  DHDIIAITRNAIKSGIDEGLIRSDVDPVEMTVFLNLIAKGLTEMSPRFKKVLEKRGITQHQFFADAADFM  HHMLMNPDRWIKTKSDVSD |
|  |  | >gi|26109888|gb|AAN82093.1|AE016766\_181 Unknown protein encoded by ISEc8 within prophage [Escherichia coli CFT073] | >ORF 1904 and organism AE010299.seq  MLTREEILEIYEAGPEAVIAVIQRLEYIIEKQASQIAELEERVRILEARLNQNSQNSSKPPSTDVFCNEK  PKPKSLRKSSGKKAGGQKGHPGKTLKLVENPDUIKYHSPEYCDHCGHHLEDTEVQDYERRQEAEIPPAQI  IFTEHRCEIKKCPHCGKVNKGSFPESIKFPIQYGPRLLASILYLRNYQFIPYERICDLVEDFYGVRISPA  TIKRAEIECFQNLQPFEEAAMKHLLASHTAHCDETGMRVLGTKWWLHVVSNNLWTYYFPHPKRGTEAMDA  LGFLPQYNGVAVHDGFASYNKYECEHALCNAHLKRELTGIEENFEQQWAKEINELLSEMKKYTDECREME  IPIDPEKVRELEGIYDAIMQGGIEENPPPDPLKEQVKKRGRKAQTKAKNLLDRFILHKEQILRFLNNLRV  SFDNNQAERDIRMMKLQQKISGTFRSIEGAVAFCRIRAYISSIKKNELNVMDAILAALNGAPLLA |
|  |  | >gi|56381040|dbj|BAD76948.1| transposase [Geobacillus kaustophilus HTA426] | >ORF 6333 and organism AE010299.seq  MPTREEILVLCASNPEVITYIVSLESQIKELTERLIALDSRLNQNSRNSSRPPSTDYFVKEKPNPKSLRK  KSGKKPGGQDGHPGTTLEMVDEPEUEIEHSLSCCKECGHTLENVEVEAYEKRQVFDIPPVNLIVTEHKSQ  IKTCPHCGRLNKAAFPESVKYPVQYGPNILASAVYCKNHHFIPYERISEFFEDIMGIKICPATIIRAEKE  CFQNLEKFESIIRERLLASPVIHCDETGMKIEGKRHWLHVASNDKYTCYFPHSKRGSEAINAMGILPEFK  GVAVHDGWKPYNTYDCDHALCNAHLQRELTGIEENYKQQWAKEMNELLTEMKKYTDECKEQLKELDFEQI  KALEERFDAAVMKGIEENPLALNPEKQGKRGKKPKTKARNLLDRFIEHKEKILRFLTDLKVPFENNQAER  DIRMMKLQQKISGTFRTIQGAEAFCRIRAYISTIRKNGLSVLEGIIAVLKGAPLTIP |
|  |  | >gi|15622302|dbj|BAB66294.1| 106aa long conserved hypothetical protein [Sulfolobus tokodaii str. 7]  >gi|3256403|dbj|BAA29086.1| 173aa long hypothetical protein [Pyrococcus horikoshii OT3] | >ORF 2185 and organism AE010299.seq  VPPRSRMVTALGLSQPVTKVMLSPPTFFSSTSLAKPRSSGVISSRLVMSLPPVALASFSMSLFLTLRIAK  MPAFARUCWARSSTPFWQKTTFAPLLITFWTIFLSMASSWSRKDWSWSGEVIEISASTSVSLSSIALFRR  AIFASLTFSGMFV |
|  |  | >gi|26109888|gb|AAN82093.1|AE016766\_181 Unknown protein encoded by ISEc8 within prophage [Escherichia coli CFT073] | >ORF 3550 and organism AE010299.seq  MLTREEILEIYEAGPEAVIAVIQRLEYIIEKQASQIAELEERVRILEARLNQNSQNSSKPPSTDVFCNEK  PKPKSLRKSSGKKAGGQKGHPGKTLKLVENPDUIKYHSPEYCDHCGHHLEDTEVQDYERRQEAEIPPAQI  IFTEHRCEIKKCPHCGKVNKGSFPESIKFPIQYGPRLLASILYLRNYQFIPYERICDLVEDFYGVRISPA  TIKRAEIECFQNLQPFEEAAMKHLLASHTAHCDETGMRVLGTKWWLHVVSNNLWTYYFPHPKRGTEAMDA  LGFLPQYNGVAVHDGFASYNKYECEHALCNAHLKRELTGIEENFEQQWAKEINELLSEMKKYTDECREME  IPIDPEKVRELEGIYDAIMQGGIEENPPPDPLKEQVKKRGRKAQTKAKNLLDRFILHKEQILRFLNNLRV  SFDNNQAERDIRMMKLQQKISGTFRSIEGAVAFCRIRAYISSIKKNELNVMDAILAALNGAPLLA |
|  |  | >gi|20904923|gb|AAM30236.1| conserved protein [Methanosarcina mazei Goe1]  >gi|29896157|gb|AAP09437.1| phosphoesterase [Bacillus cereus ATCC 14579] | >ORF 3594 and organism AE010299.seq  LSSNSYFGKYQLKISEIFQGLIELCHNPYFQKENWSRQNYGNKNVKFPAIIKYFIIVFAFLTTCSVYSFI  EPYLIEEQTTIISDSDVPQNFVGKKIIFISDIHHGUFFERERVAALVRKVNELDPDNIVLGGVAGNRRR |
|  |  | >gi|20906100|gb|AAM31298.1| Cobalamin biosynthesis protein CobN [Methanosarcina mazei Goe1]  >gi|20906098|gb|AAM31297.1| Cobalamin biosynthesis protein [Methanosarcina mazei Goe1]  >gi|2622026|gb|AAB85426.1| cobalamin biosynthesis protein N [Methanothermobacter thermautotrophicus]  >gi|2621801|gb|AAB85219.1| magnesium chelatase subunit [Methanothermobacter thermautotrophicus] | >ORF 4474 and organism AE010299.seq  MATYEWLVKNGYNESAASEYSLIRIFSASEGSYGPSISVPIGASGSWEDDSVIGNYFIDGWGYAYGENLW  GEQLQDIFRQNLNGVEVVTHSISSNNYGVLYGDGYFSDLGGLALAVRTVSGQTPEIYLSNLRDPNNAVVE  TLSQFLVREIRTRNLNPEWIKGMMEHEYYGASILSSGLENLWGWEVTTPDLITDETWTEMYDVYIQDKYD  LGMEEFFDANNPWARQSMEARMLEAIRKGYWDADUETIDALTREYVESVVESGVTCCHHTCGNPLLDDYI  TGVVSAPNANVVDTDVMEDYERLMAEARGETLDTDSPEGSKHSSSTGAKAEVVSKEEYYSENANSTLEID  SGVGTDPARSPTGEADVKNEYIEGYELTKEKIKDDVESDSMPFSASDLVGMLFLLLFMGAVFVGYRRKKN |
|  |  | >gi|3257096|dbj|BAA29779.1| 146aa long hypothetical protein [Pyrococcus horikoshii OT3] | >ORF 5101 and organism AE010299.seq  LDIISGPLMEMKUASDSLATAFASIVLPVPGGPCSSTPFGASIPSLSKSSGCFRGSSIISLTLLMASLSP  PRSSYITVGIFCSEIISIASGRSSTCVISVILTIPAGAVDTTCSLISPSPKDGPKPCPKKS |
|  |  | gi|56381040|dbj|BAD76948.1| transposase [Geobacillus kaustophilus HTA426] | >ORF 5992 and organism AE010299.seq  MLTREEILEIYEAGPEAVIAVIQRLEYIIEKQASQIAELEERVRILEARLNQNSQNSSKPPSTDVFCNEK  PKPKSLRKSSGKKAGGQKGHPGKTLKLVENPDUIKYHSPEYCDHCGHHLEDTEVQDYERRQEAEIPPAQI  IFTEHRCEIKKCPHCGKVNKGSFPESIKFPIQYGPRLLASILYLRNYQFIPYERICDLVEDFYGVRISPA  TIKRAEIECFQNLQPFEEAAMKHLLASHTAHCDETGMRVLGTKWWLHVVSNNLWTYYFPHPKRGTEAMDA  LGFLLQYNGVAVHDGFASYNKYECEHALCNAHLKRELTGIEENFEQQWAKEINELLSEMKKYTDECREME  IPIDPEKVRELEGIYDAIMQGGIEENPPPDPLKEQVKKRGRKAQTKAKNLLDRFILHKEQILRFLNNLRV  SFDNNQAERDIRMMKLQQKISGTFRSIEGAVAFCRIRAYISSIKKNELNVMDAILAALNGAPLLA |
